# The Effects of Naringenin on miRNA-mRNA Profiles in HepaRG Cells

**DOI:** 10.3390/ijms22052292

**Published:** 2021-02-25

**Authors:** Weiyang Fan, Rui Shi, Minyi Guan, Pan Chen, Hao Wu, Weiwei Su, Yonggang Wang, Peibo Li

**Affiliations:** Guangdong Engineering and Technology Research Center for Quality and Efficacy Re-Evaluation of Post-Marketed TCM, Guangdong Key Laboratory of Plant Resources, School of Life Sciences, Sun Yat-sen University, Guangzhou 510275, China; fanwy5@mail2.sysu.edu.cn (W.F.); ruishi900930@gmail.com (R.S.); mandyguan723@163.com (M.G.); chenpan989@126.com (P.C.); wuhao_cpu@126.com (H.W.); lsssww@mail.sysu.edu.cn (W.S.); wangyg@mail.sysu.edu.cn (Y.W.)

**Keywords:** naringenin, mRNA-seq, miRNA-seq, HepaRG cells

## Abstract

Naringenin, a natural flavonoid widely found in citrus fruits, has been reported to possess anti-oxidant, anti-inflammatory, and hepatoprotective properties as a natural dietary supplement. However, the regulatory mechanism of naringenin in human liver remains unclear. In the present study, messenger RNA sequencing (mRNA-seq), microRNA sequencing (miRNA-seq), and real-time qPCR were used to distinguish the expression differences between control and naringenin-treated HepaRG cells. We obtained 1037 differentially expressed mRNAs and 234 miRNAs. According to the target prediction and integration analysis in silico, we found 20 potential miRNA-mRNA pairs involved in liver metabolism. This study is the first to provide a perspective of miRNA–mRNA interactions in the regulation of naringenin via an integrated analysis of mRNA-seq and miRNA-seq in HepaRG cells, which further characterizes the nutraceutical value of naringenin as a food additive.

## 1. Introduction

**Figure 1:**
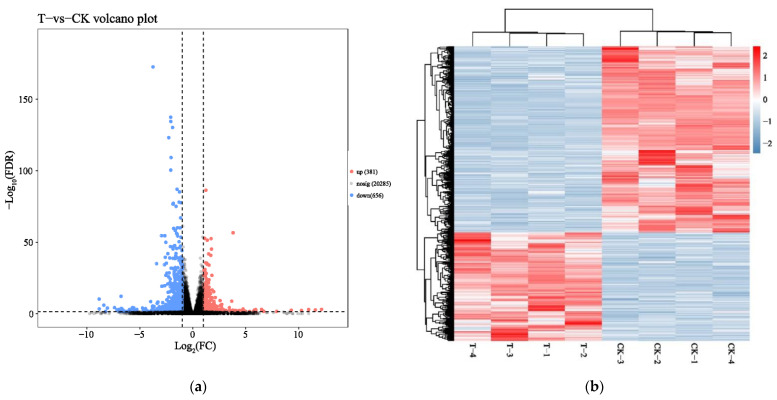
Identification of differentially expressed messenger RNAs (mRNAs) in response to naringenin. (**a**) Volcano plot showed that all non-redundant unigenes were identified in the control group and the naringenin group. The 20,285 gray dots represent non-significantly differentially expressed mRNAs, the 381 red dots represent significantly differentially up-regulated mRNAs, and the 656 blue dots represent significantly differentially down-regulated mRNAs; FC (fold change) = the naringenin group/the control group; FDR (false discovery rate), the expected percent of false predictions in the set of predictions; (**b**) heat map showing 1037 differentially expressed genes (DEGs), comparing the control group with the naringenin group. Each row represents one mRNA, and each column represents a sample. Red, upregulation; blue, downregulation; CK-1, CK-2, CK-3, and CK-4, the control group; T-1, T-2, T-3, and T-4, the naringenin group.

Naringenin, a natural flavonoid, is found abundantly in citrus fruits and other edible fruits, like grapefruit, oranges, bergamot, tomatoes, and figs [1]. After oral administration, naringenin is widely distributed in the gastrointestinal tract and liver [2,3] and exhibits a direct antioxidant property by free radical scavenging activity on account of its molecular structure, and induces the endogenous antioxidant system in the liver [4]. Growing evidence from both in vitro and in vivo studies has identified various protective capacities of naringenin, such as anti-inflammatory [5], antioxidant [6], anti-fibrosis [7], and hepatoprotective [4,8] activities. These capacities suggest that dietary naringenin could be applied to prevent metabolic syndrome and malignant diseases, including fulminant hepatitis, fatty liver disease, fibrosis, etc. [9,10]. However, there are few detailed studies about the regulatory effects of naringenin on the overall genes of liver [4].

MicroRNAs (miRNAs) are a class of non-coding, single-stranded RNA molecules with a length of 18–26 nucleotides encoded by endogenous genes, which exhibit a broad range of biological regulatory functions in phylogeny, differentiation, proliferation, and apoptosis [11]. miRNA, binding messenger RNAs (mRNAs) by sequence-specific recognition, negatively regulates gene expression at the post-transcriptional level through degradation of target mRNAs [12]. Under exogenous stimulation, miRNA expression is altered, and then target mRNA expression is regulated. Eventually, extensive physiological functions are changed to cope with the challenges caused by exogenous stimulation [13]. A more reliable method for predicting miRNA–mRNA target relations is to simultaneously integrate mRNA-seq analysis with miRNA-seq analysis using a particular processing context in silico [14].

Therefore, our objective was to study the effects of naringenin on global genes and highlight the possible regulatory mechanism of miRNA-mRNA pairs in the liver. In present study, mRNA expression changes and miRNA expression profiles were investigated in HepaRG cells by performing mRNA-seq, miRNA-seq, bioinformatic analyses, and real-time qPCR to provide evidence for the potential of naringenin as a natural dietary supplement.

## 2. Results

### 2.1. Analysis of Transcriptome Sequencing in the Response to Naringenin

To identify mRNA expression changes of HepaRG cells in response to naringenin, eight complementary DNA (cDNA) libraries in the control group (CK-1, CK-2, CK-3, and CK-4) and the experimental group (T-1, T-2, T-3, and T-4) were constructed with total RNA and subjected to Illumina HiSeq2500 sequencing(Genedenovo Biotechnology Co., Ltd, Guangzhou, China). Overviews of the sequencing and assembly results for the control group and experimental group are shown in Table 1. Gene expression changes were analyzed by comparing the treated and control groups. As shown in Figure 1a, the naringenin-exposed group expressed 1037 differentially expressed genes (DEGs) compared with the control group. A heat map of 1037 DEGs showed the cluster analysis of the control group and the naringenin group (Figure 1b; 381 up- and 656 down-regulated genes; Table S1, Supplementary Materials).

**Figure 2:**
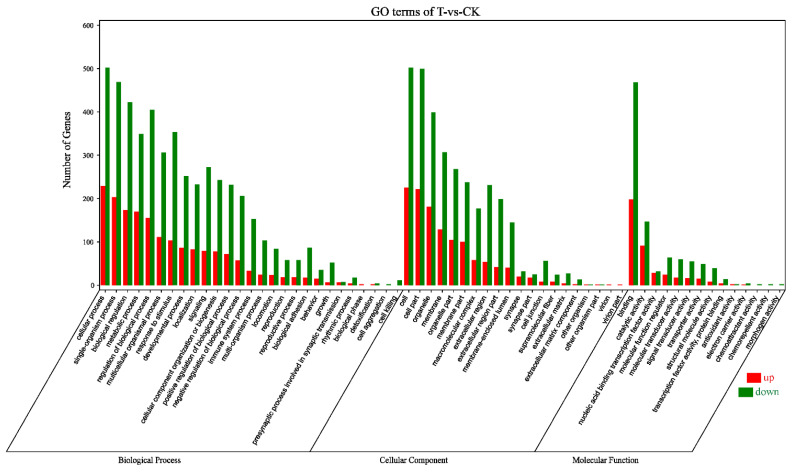
Gene Ontology (GO) terms categorization of 1037 DEGs. Number of genes: number of target genes in a term. Red, upregulation; green, downregulation; CK, the control group; T, the naringenin group.

According to the Gene Ontology (GO) classification system, 1037 DEGs were classified into three major functional categories (biological process, cellular component, and molecular function) and 61 subcategories (Figure 2). Among the biological process, cellular process (731) was the most commonly represented, followed by single-organism process (672) and biological regulation (595). In the category of cellular component, a significant proportion of clusters was assigned to cell (727), cell part (721), and organelle (580). Genes involved in binding (666) and catalytic activity (238) groups were notably represented in the molecular function category.

**Figure 3:**
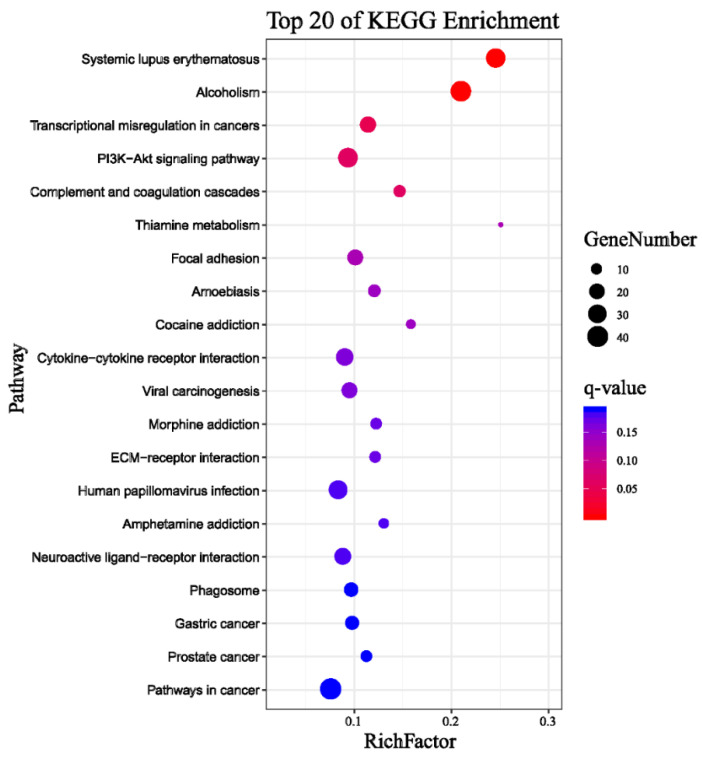
Top 20 pathways of Kyoto Encyclopedia of Genes and Genomes (KEGG) terms for 1037 DEGs. GeneNumber: number of target genes in a pathway. RichFactor: ratio of number of target genes divided by number of all the genes in a term or pathway.

The Kyoto Encyclopedia of Genes and Genomes (KEGG) classification was found for 1037 DEGs that were further classified into the top twenty biochemical pathways according to the smallest q-value and the largest GeneNumber in pathway annotation (Figure 3).The GeneNumber and ratio of annotated genes of the top five pathways are systemic lupus erythematosus (33, 8.4%), alcoholism (38, 9.67%), transcriptional misregulation in cancers (22, 5.6%), PI3K–Akt signaling pathway (34, 8.65%) and complement and coagulation cascades (12, 3.05%). There were more than 10 enriched genes among the five pathways. Overall, undergoing naringenin treatment had a significant impact on the global gene expression profile of HepaRG cells. These results implied that the genes involved in these pathways may play crucial roles in naringenin regulation.

### 2.2. Analysis of miRNA Transcript Levels in Response to Naringenin

**Figure 4:**
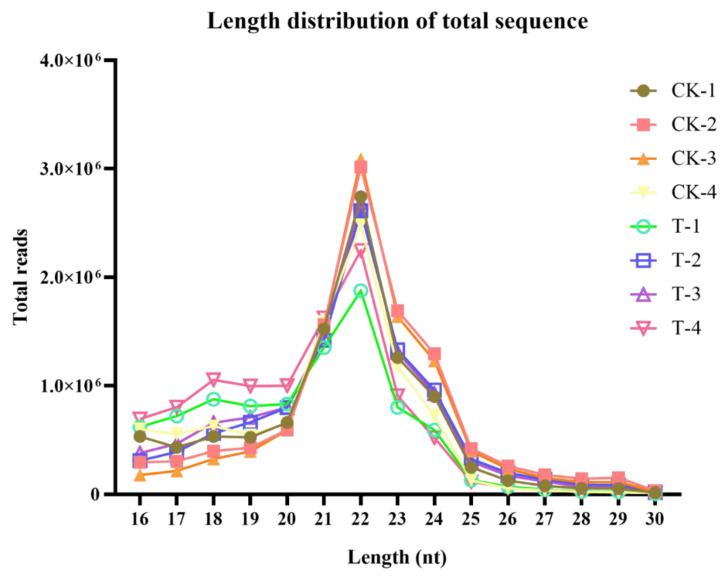
The length distribution of the small RNA sequence. CK-1, CK-2, CK-3, and CK-4, the control group; T-1, T-2, T-3, and T-4, the naringenin group.

In this study, we aimed to determine whether naringenin exposure alters the expression levels of miRNAs in HepaRG cells. After exposure, we collected small RNAs and measured their relative abundance using Illumina HiSeq2500 (Genedenovo Biotechnology Co., Ltd, Guangzhou, China). As shown in Table 2, clean reads of eight samples were generated, respectively, after removing contaminant reads. An overview of reads for small RNA sequencing from raw data to high quality and with quality filtering is provided in Table 2. The length distributions of small RNAs were similar among libraries in that 21–23 nt RNAs were the most abundant (Figure 4).

**Figure 5:**
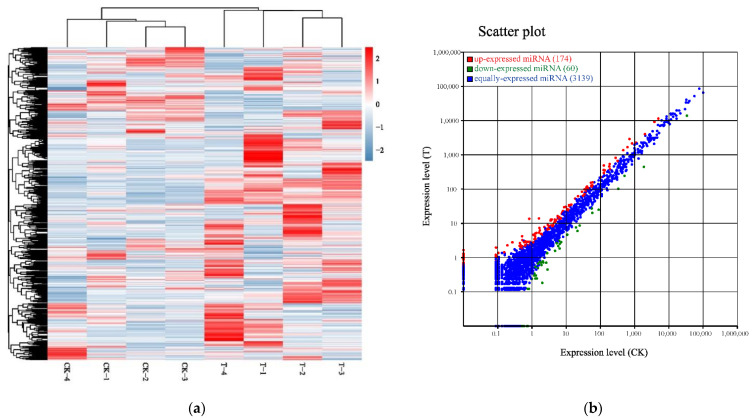
Identification of differentially expressed microRNA (miRNAs) in response to naringenin. (**a**) Heat map of 3373 expressed miRNAs in response to naringenin. Each row represents one miRNA, and each column represents a sample. Red, upregulation; blue, downregulation; CK-1, CK-2, CK-3, and CK-4, the control group; T-1, T-2, T-3, and T-4, the naringenin group; (**b**) scatter plot showing 234 DEMs (174 up- and 60 down-regulated) comparing the control group with the naringenin group. Each dot represents one miRNA. Red, upregulation; green, downregulation; blue, non-significance. CK, the control group; T, the naringenin group.

All the small RNAs were aligned in the GeneBank database (Release 209.0) and the Rfam database (11.0) to identify and remove ribosomal RNA (rRNA), small conditional RNA (scRNA), small nucleolar (snoRNA), small nuclear (snRNA), and transfer RNA (tRNA). In accordance with reference genome, these small RNAs, mapped to exons, introns, and repeat sequences, were also removed. The filtering small RNAs were searched against miRBase database (Release 21) to identify miRNAs. The heat map of 3373 miRNAs shows the cluster analysis of the control group and the naringenin group in Figure 5a. Illumina HiSeq2500 profiling of the 3373 miRNAs analyzed in naringenin exposure vs. control samples showed that a total of 234 differentially expressed miRNAs (DEMs, 174 up- and 60 down-regulated; Table S2, Supplementary Materials) were detectable in HepaRG cells (Figure 5b).

### 2.3. Target Prediction and Integration Analysis of mRNA and miRNA Expression Profiles in Response to Naringenin

**Figure 6:**
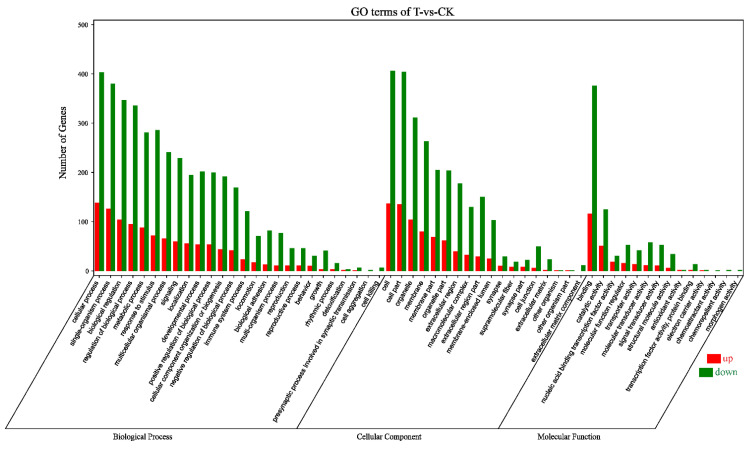
Gene Ontology terms categorization of 681 DEGs. Number of genes: number of target genes in a term. Red, upregulation; green, downregulation; CK, the control group; T, the naringenin group.

Acting at the post-transcriptional level, miRNAs silence and/or down-regulate cellular mRNA gene expression by target RNA cleavage. To predict the target genes of 234 DEMs, we performed computational analyses using the RNAhybrid (v2.1.2) + svmlight (v6.01), Miranda (v3.3a), and TargetScan (Version: 7.0). The simultaneous profiling of 234 DEMs and 1037 DEGs levels in silico can identify the presumptive target mRNAs of miRNAs. We selected the intersection of DEGs and target genes of DEMs, and then performed bioinformatics analysis on these intersection genes. A total of 5607 negative miRNA-mRNA pairs for naringenin treatment were obtained, with the involvement of 216 DEMs and 681 DEGs (Table S3, Supplementary Materials). In line with the GO classification system, 681 DEGs were classified into 58 subcategories (Figure 6). The first three subcategories had not changed in three major functional categories compared with transcriptome sequencing analysis (Figure 2 and Figure 6).

**Figure 7:**
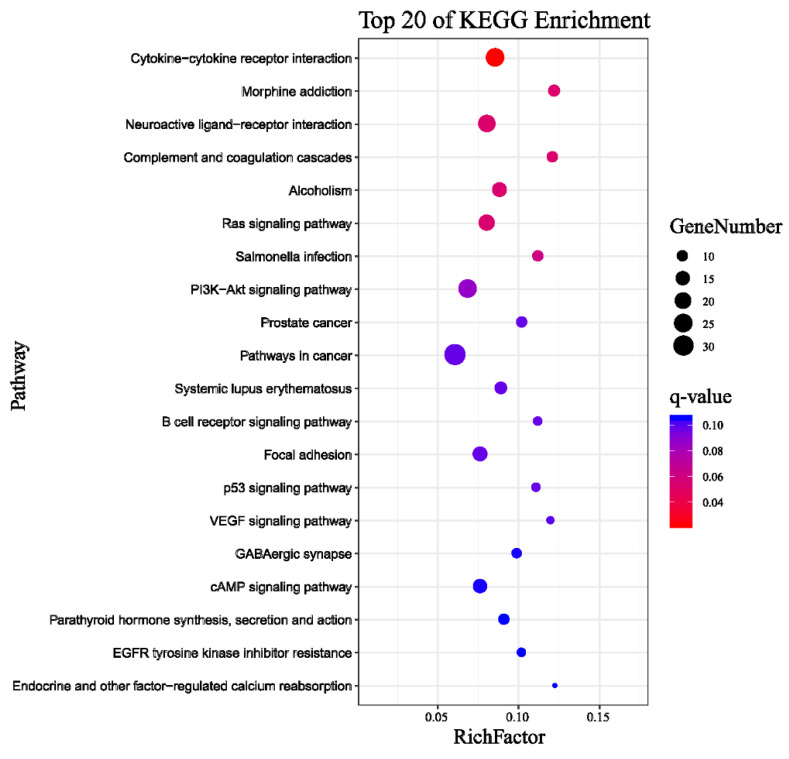
Top 20 pathways of KEGG terms for 681 DEGs. GeneNumber: number of target genes in a pathway. RichFactor: ratio of number of target genes divided by number of all the genes in a term or pathway.

Pathway enrichment analysis for 681 DEGs of 5607 negative miRNA-mRNA pairs identified the top twenty pathways according to the smallest q-value and the largest GeneNumber in pathway annotation after naringenin exposure (Figure 7): with PI3K–Akt signaling pathway (25, 8.96%) being the eighth pathway and the second-most abundant.

### 2.4. Real-Time qPCR Validation of Naringenin Regulation in Liver Metabolism and Potential Regulatory miRNA-mRNA Pairs

According to global gene function annotations, literature review, and their potential relationship with naringenin-responsive miRNAs, 19 DEGs (ALOX15, CA9, TH, HKDC1, NDUFA4L2, RRM2, ACSL5, PLA2G4C, LIPT2, UGDH, FTCD, ABAT, AZIN2, HS6ST3, B4GALT6, GUSB, DCT, ALAS2, and MAT1A) were manually selected as representatives for their potential roles in liver metabolism. In addition, the PI3K–Akt signaling pathway had been significantly enriched (fourth in the KEGG of RNA-seq analysis, Figure 3; eighth in the KEGG of miRNA-RNA-seq analysis, Figure 7); therefore, 11 DEGs (PDGFRB, CSF1R, FGFR2, IL2RG, IL7R, ITGB4, GNG4, PCK1, CREB3L3, CREB3L1, and NFκB1) among the PI3K–Akt signaling pathway were screened out.

**Figure 8:**
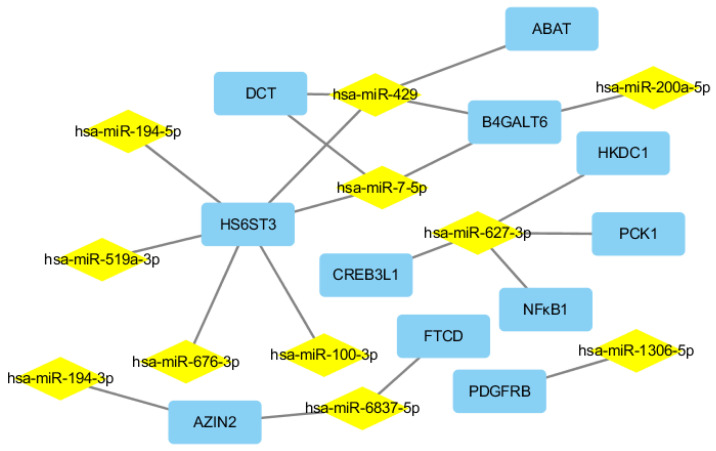
Putative miRNA–mRNA negative correlation network in response to naringenin. Rectangular nodes, mRNAs; diamond nodes, miRNAs.

**Figure 9:**
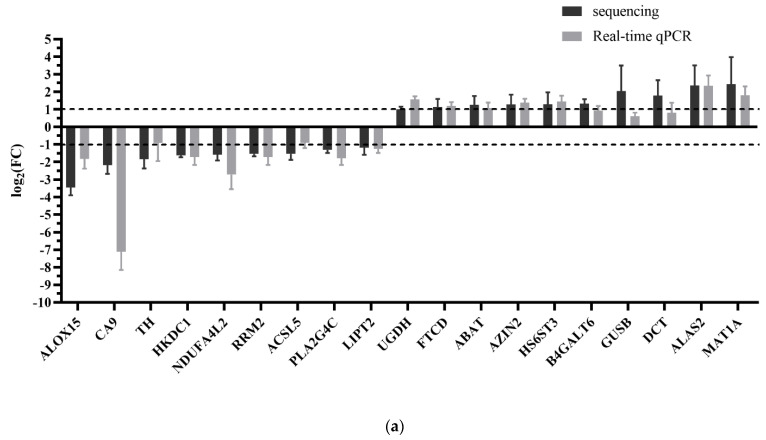

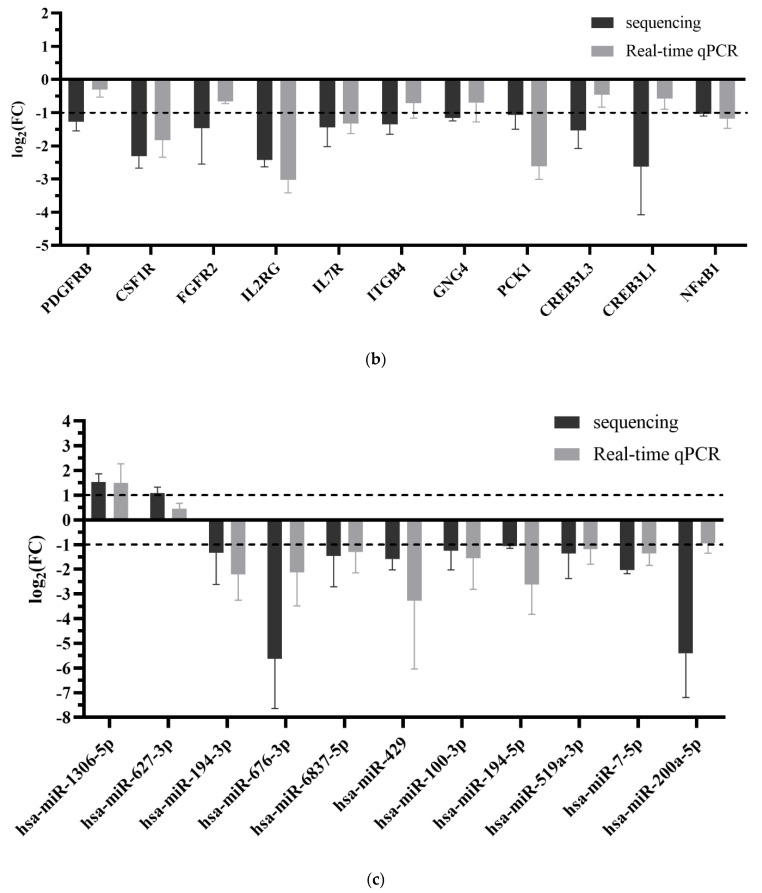
Relative mRNA and miRNA expression of the control group and the naringenin group, in respect to RNA-seq and real-time qPCR. (**a**) The 19 genes involved in liver metabolism; (**b**) 11 genes involved in the PI3K–Akt signaling pathway; (**c**) 11 putative regulatory miRNAs. Y-axis represents log_2_ (FC); FC (fold change) = the naringenin group/the control group. The dashed line indicated fold change data of 2.0. Values are the mean ± SD (*n* = 4).

We here described the interaction between the 30 DEGs and 11 human miRNAs (hsa-miR-1306-5p, hsa-miR-627-3p, hsa-miR-194-3p, hsa-miR-676-3p, hsa-miR-6837-5p, hsa-miR-429, hsa-miR-100-3p, hsa-miR-194-5p, hsa-miR-519a-3p, hsa-miR-7-5p, and hsa-miR-200a-5p), including 20 negative miRNA–mRNA interactions (Figure 8). As shown in Figure 8, a single miRNA can regulate multiple target mRNAs and vice versa (e.g., ABAT, HS6ST3, B4GALT6, and DCT could possibly be simultaneously regulated by hsa-miR-429; HS6ST3 may be simultaneously regulated by hsa-miR-429, hsa-miR-100-3p, hsa-miR-519a-3p, hsa-miR-676-3p, hsa-miR-7-5p, and hsa-miR-194-5p; some DEGs had no paired DEMs). The expression profiles of 19 DEGs related to liver metabolism and 11 DEGs among the PI3K–Akt signaling pathway were further validated using real-time qPCR (Figure 9a,b). The expression level of 11 human miRNAs (two up- and nine down-regulated) was further assessed for naringenin-induced changes by real-time qPCR consistent with sequencing results (Figure 9c). Although there were some quantitative differences between the two analytical platforms, the similarities between the RNA-seq data and the real-time qPCR suggested that the RNA-seq data were reproducible and reliable.

## 3. Discussion and Conclusions

The findings discussed here reveal the first detailed information regarding parallel mRNA and miRNA expression changes in HepaRG cells in response to naringenin. We performed an integrative analysis of these data including 234 DEMs and 1037 DEGs induced by naringenin, which provide global insight into the miRNA–mRNA interactions of naringenin in the regulatory mechanism. According to the gene function annotations and literature review, 19 DEGs related to metabolism were screened out. In particular, the PI3K–Akt signaling pathway was significantly enriched both in analysis of transcriptome sequencing (the third-most abundant, and ranked fourth) and integration analysis of miRNA-mRNA expression profiles (the second-most abundant, and ranked eighth) in responses to naringenin. In addition, 11 DEGs in the PI3K–Akt signaling pathway were further validated using real-time qPCR analysis. In this work, we constructed a miRNA-mRNA regulatory network according to the DEMs and DEGs datasets and miRNA-targeting information. Some studies have demonstrated that the miRNA–mRNA regulatory network responds to liver damage, including hepatocellular carcinoma and oxidative stress [15]. Although several miRNA-induced RNA activation phenomena were identified [16], under most circumstances, the negative correlation between miRNAs and their target mRNAs is often considered support for miRNA targeting [17]. Ultimately, 20 miRNA-mRNA negative correlation pairs were identified with the involvement of liver metabolism and the PI3K–Akt signaling pathway.

With regard to global genes, we addressed our particular research question using pathway analysis to highlight 19 DEGs related to the functional clusters: metabolism, including Lipid metabolism (ALOX15, ACSL5, PLA2G4C, and B4GALT6), Energy metabolism (CA9 and NDUFA4L2), Metabolism of cofactors and vitamins (TH, LIPT2, and FTCD), Amino acid metabolism (RRM2, AZIN2, DCT, ALAS2, and MAT1A), Glycan biosynthesis and metabolism (HS6ST3 and GUSB), and Carbohydrate metabolism” (HKDC1, PCK1, UGDH, and ABAT). These 19 DEGs enriched to metabolism are primarily involved in insulin sensitivity, lipid accumulation, glycogen storage, and energy expenditure. Previous studies reported that the down-regulation of ALOX15 in alcohol-induced mice liver damage [18], CA9 in BALB/c mice [19], TH in nonalcoholic fatty liver disease (NAFLD) [20], HKDC1 [21], and the up-regulation of UGDH [22] can significantly relieve oxidative stress, lipid accumulation, and liver damage. Knockdown of NDUFA4L2 suppressed ROS accumulation and apoptosis in hepatocellular carcinoma (HCC) cells [23]. RRM2 silencing inhibited NCI-H929 cell proliferation [24], and FTCD overexpression suppressed cell proliferation by promoting DNA damage and inducing cell apoptosis in HCC cells [25]. Ablation of ACSL5 improved insulin sensitivity, increased energy expenditure, and delayed triglyceride absorption in mice [26]. DCT supplementation improved age-associated liver steatosis and inflammation [27]. Lipid droplet formation upon fatty acid and hepatitis C virus stimulation in PLA2G4C knockdown cells was impaired [28]. The down-regulation of LIPT2 inhibited fatty acid synthesis [29]. The up-regulation of ABAT [30], AZIN2 [31], B4GALT6 [32], HS6ST3 [33], and GUSB [34] could promote fatty acid and glycogen decomposition. ALAS2 overexpression could improve liver hematopoietic capacity [35], and MAT1A expressed in hepatocytes maintained the differentiated state of these cells [36]. Consistent with the findings described above, the regulation of these metabolic genes in our results may be favorable to improve metabolism in response to naringenin.

The PI3K–Akt signaling pathway may offer clues for the molecular mechanism involved in metabolism, inflammation, and oxidative stress [37], which plays a pivotal role in the response to naringenin. The PI3K–Akt signaling pathway has diverse downstream effects on cellular metabolism through either direct regulation of nutrient transporters and metabolic enzymes or the control of transcription factors that regulate the expression of key components of metabolic pathways [38,39], including glucose metabolism, biosynthesis of macromolecules, and maintenance of redox balance. It was reported that PDGFRB silencing inhibited the activation and proliferation of hepatic stellate cells and ameliorated liver fibrosis [40]. The collaborative inhibition of CSF1R and FGFR2 is expected to enhance the antitumor effects by targeting immune evasion and angiogenesis in the tumor microenvironment [41]. Knocking down ITGB4 suppressed glycolysis in cancer-associated fibroblasts [42]. Genetic knockdown of PCK1 prevented fatty-acid-induced rise in oxidative flux, oxidative stress, and inflammation [43], which was also correlated with signaling pathways governed by insulin [44]. It was shown that overexpression of nuclear CREB3L3 induced systemic lipolysis, hepatic ketogenesis, and insulin sensitivity with increased energy expenditure [45]. Inhibition of CREB3L1 reportedly blocked cancer invasion and metastasis [46]. NFκB is a key regulator of immune development, immune responses, inflammation, and cancer [47]. It has been well-established that suppressing NFκB transduces anti-inflammatory signals and reduces inflammation [48]. In the PI3K–Akt signaling pathway, our results showed that naringenin significantly down-regulated the mRNA expressions of PDGFRB, PCK1, CREB3L1, and NFκB1 with related miRNAs (has-miR-1306-5p and hsa-miR-627-3p) being significantly down-regulated. Therefore, our data suggested that naringenin may play a salutary role in anti-inflammatory, anti-oxidative stress, and ameliorative metabolism via the inhibition of the PI3K–Akt signaling pathway.

This study was the first to integrated the analysis of mRNA-seq and miRNA-seq in the liver in response to naringenin, and provide a perspective of metabolism in naringenin regulation. In terms of metabolism and the PI3K–Akt signaling pathway, the 11 DEMs, 30 DEGs, and 20 miRNA-mRNA pairs need more research for the activities of naringenin. There are some limitations of this research; for example, the dose-dependent and time-dependent effects of naringenin in miRNA-mRNA interactions were still unclear. Although given miRNAs analyzed in silico suggested regulatory capacities, their functions need to be further certified in a specific context within a living system. In summary, we provided preliminary research analyzing mRNA and miRNA expression and profiling of metabolism. The regulatory mechanism of miRNA-mRNA pairs could be additional possible evidence for annotating the nutraceutical value of naringenin.

## 4. Materials and Methods

### 4.1. Chemicals and Reagents

The HepaRG cell line was originally purchased from Biopredic International (Rennes, France). RPMI-1640 medium and penicillin-streptomycin-glutamine solution were obtained from Gibco (Gaithersburg, MD, USA). Fetal bovine serum (FBS) was purchased from Corning (Auckland, New Zealand). Naringenin and dimethyl sulfoxide (DMSO) were from Sigma-Aldrich (St. Louis, MO, USA). TRIzol™ reagent was supplied by Thermo Fisher (Carlsbad, CA, USA). Ultrapure water was purified by a Milli-Q academic water purification system (Millipore, Bedford, MA, USA). All other reagents were commercialized products of the highest analytical grade available.

### 4.2. HepaRG Cells Culture

The HepaRG cells were seeded at 5 × 10^4^ cells/cm^2^ in six-well plates and grown in RPMI-1640 medium, cultured with or without 100 μM naringenin for 48 h, and supplemented with 10% FBS and 1% antibiotics (100 U/mL penicillin and 100 μg/mL streptomycin) at 37 °C in a humidified 5% CO_2_ incubator. CK-1, CK-2, CK-3, and CK-4 refer to the control check group; T-1, T-2, T-3, and T-4 refer to the 100 μM naringenin treatment group. The numbers 1, 2, 3, and 4 represent samples from four independent repeated experiments.

### 4.3. Total RNA Extraction

HepaRG cells were washed twice with ice-cold phosphate-buffered saline (PBS) and harvested with TRIzol™ reagent as recommended by the manufacturer. Thermo Scientific NanoDrop™ 2000 c Spectrophotometers (Wilmington, DE, USA) were used to measure the RNA quality and quantity of each sample according to the manufacturers’ protocol.

### 4.4. RNA Sequencing

The mRNA was enriched by Oligo(dT) beads, then the enriched mRNA was fragmented and reverse-transcripted into cDNA with random primers by QiaQuick PCR extraction kit (Qiagen, Venlo, The Netherlands). RNA molecules in a size range of 18–30 nt were enriched by polyacrylamide gel electrophoresis. The 3′ and 5′ adapters were added, then enriched RNAs were reverse-transcripted by the QiaQuick PCR extraction kit, according to the manufacturer’s instructions (Qiagen, Venlo, The Netherlands). The ligation products were size-selected by agarose gel electrophoresis. There were four samples in the naringenin group and four samples in the control group. Each sample generated two cDNA libraries: one for mRNA-seq and the other for miRNA-seq. PCR amplified products were enriched to respectively generate 16 cDNA libraries and sequenced using Illumina HiSeq2500 by Genedenovo Biotechnology Co. (Guangzhou, China). The RNA and small RNA sequencing data were deposited in the NCBI Sequence Read Archive (accession numbers from SRR13675952 to SRR13675963).

### 4.5. Real-Time qPCR

Transcription of mRNA into cDNA was conducted with the GoScript™ Reverse Transcription System (Promega, Madison, WI, USA) from 3 μg of total RNA, according to the manufacturer’s instructions. For miRNA analysis, cDNA-synthesis was performed with the miRNA First Strand cDNA Synthesis (Tailing Reaction, Sangon Biotech, Shanghai, China) from 2 μg of total RNA.

The real-time qPCR was carried out with GoTaq^®^ qPCR Master Mix (Promega, Madison, WI, USA) on a LightCycler 480 (Roche, Mannheim, Germany), as recommended by the manufacturer. The thermal cycling procedure started with an initial denaturation at 95 °C for 10 min. This was followed by 45 cycles of denaturation for 10 s at 95 °C, primer binding for 20 s at 60 °C, and elongation for 20 s at 72 °C. The procedure ended with a final amplification at 95 °C for 5 s, 65 °C for 1 min, the addition of a dissociation curve step, and a cooling step. Primers were purchased from Sangon Biotech (Shanghai, China). The primer pairs’ sequences used for the validation of the signature are described in Table 3 and Table 4. *C*t-values were calculated in reference to β-actin or U6.

### 4.6. Bioinformatic Analysis and SStatistics

DEGs and DEMs were identified using an R-based software package. The threshold value for selection of DEGs and DEMs was *q*-value (adjusted *p*-value) ≤ 0.05 and fold change (FC) ≥2 or ≤0.5. KEGG and GO classification including molecular functions, biological processes, and cellular components were used to analyze DEGs and DEMs. The results of biological assay are presented in the form of mean ± SD based on four independent experiments in GraphPad Prism 8.

## Figures and Tables

**Table 1:** Summary of sequence data generated for HepaRG cells transcriptome and quality filtering.

Sample	Raw Data	Clean Data (%)	Raw Data (bp)	Clean Data (bp)	After Filtering Q20 (%)	After Filtering Q30 (%)	After Filtering N (%)	After Filtering GC (%)
CK-1	47,663,932	47,577,906 (99.82%)	7,149,589,800	7,114,281,738	6,992,811,543 (98.29%)	6,756,607,154 (94.97%)	19,427 (0.00%)	3,804,122,670 (53.47%)
CK-2	42,079,942	42,026,040 (99.87%)	6,311,991,300	6,283,554,811	6,189,856,925 (98.51%)	6,000,864,261 (95.50%)	8392 (0.00%)	3,360,455,066 (53.48%)
CK-3	41,188,030	41,131,120 (99.86%)	6,178,204,500	6,147,638,474	6,050,714,205 (98.42%)	5,857,735,081 (95.28%)	8775 (0.00%)	3,273,834,153 (53.25%)
CK-4	43,096,530	43,039,450 (99.87%)	6,464,479,500	6,433,433,599	6,333,636,124 (98.45%)	6,134,820,735 (95.36%)	8660 (0.00%)	3,439,680,095 (53.47%)
T-1	51,658,770	51,539,258 (99.77%)	7,748,815,500	7,706,270,162	7,562,514,956 (98.13%)	7,292,630,398 (94.63%)	26,068 (0.00%)	4,098,720,423 (53.19%)
T-2	48,606,134	48,501,812 (99.79%)	7,290,920,100	7,250,461,713	7,113,064,471 (98.10%)	6,85,1340,567 (94.50%)	23,956 (0.00%)	3,887,698,839 (53.62%)
T-3	62,602,866	62,433,856 (99.73%)	9,390,429,900	9,333,102,578	9,155,158,477 (98.09%)	8,825,370,611 (94.56%)	45,571 (0.00%)	4,878,494,560 (52.27%)
T-4	41,346,744	41,292,738 (99.87%)	6,202,011,600	6,166,582,431	6,070,483,986 (98.44%)	5,876,108,215 (95.29%)	8644 (0.00%)	3,277,271,342 (53.15%)

CK-1, CK-2, CK-3, and CK-4, the control group; T-1, T-2, T-3, and T-4, the naringenin group.

**Table 2:** Summary of sequence data generated for HepaRG cells’ small RNA and quality filtering.

Sample	Clean Reads	High Quality	Smaller than 18 Nt	Polya	Low Cutoff	Clean Tags
CK-1	13,123,917	13,076,410	2,570,571	540	328,987	10,051,993
	(100%)	(99.6380%)	(19.6581%)	(0.0041%)	(2.5159%)	(76.8712%)
CK-2	13,744,858	13,687,348	1,981,919	807	443,665	11,100,847
	(100%)	(99.5816%)	(14.4799%)	(0.0059%)	(3.2414%)	(81.1030%)
CK-3	12,609,501	12,563,626	1,535,545	766	306,124	10,571,146
	(100%)	(99.6362%)	(12.2221%)	(0.0061%)	(2.4366%)	(84.1409%)
CK-4	13,923,071	13,865,587	3,070,687	377	386,734	10,259,540
	(100%)	(99.5871%)	(22.1461%)	(0.0027%)	(2.7892%)	(73.9928%)
T-1	12,156,840	12,100,462	2,354,606	464	465,005	9,161,791
	(100%)	(99.5362%)	(19.4588%)	(0.0038%)	(3.8429%)	(75.7144%)
T-2	12,620,973	12,574,364	1,472,821	741	337,320	10,592,975
		(99.6307%)	(11.7129%)	(0.0059%)	(2.6826%)	(84.2426%)
T-3	12,806,071	12,758,280	1,603,461	1012	307,185	10,700,322
	(100%)	(99.6268%)	(12.5680%)	(0.0079%)	(2.4077%)	(83.8696%)
T-4	14,891,138	14,831,250	2,514,654	556	317,307	11,831,747
	(100%)	(99.5978%)	(16.9551%)	(0.0037%)	(2.1394%)	(79.7758%)

CK-1, CK-2, CK-3, and CK-4, the control group; T-1, T-2, T-3, and T-4, the naringenin group.

**Table 3:** List of 32 mRNA primers for real-time qPCR.

Gene	Forward Sequence (5′→3′)	Reverse Sequence (5′→3′)
PDGFRB	TGCAGACATCGAGTCCTCCAAC	GCTTAGCACTGGAGACTCGTTG
CSF1R	GCTGCCTTACAACGAGAAGTGG	CATCCTCCTTGCCCAGACCAAA
FGFR2	GTGCCGAATGAAGAACACGACC	GGCGTGTTGTTATCCTCACCAG
IL2RG	CACTCTGTGGAAGTGCTCAGCA	GAGCCAACAGAGATAACCACGG
IL7R	ATCGCAGCACTCACTGACCTGT	TCAGGCACTTTACCTCCACGAG
ITGB4	AGGATGACGACGAGAAGCAGCT	ACCGAGAACTCAGGCTGCTCAA
GNG4	CTCCAGATTCAGCCTCCGTTTTG	TGCCATAGGTCTGGAAGAGGTG
PCK1	CATTGCCTGGATGAAGTTTGACG	GGGTTGGTCTTCACTGAAGTCC
CREB3L3	GAAGCCTCTGTGACCATAGACC	GGAGGTCTTTCACGGTGAGATTG
CREB3L1	GCCTTGTGCTTTGTTCTGGTGC	CCGTCATCGTAGAATAGGAGGC
NFκB1	GCAGCACTACTTCTTGACCACC	TCTGCTCCTGAGCATTGACGTC
ALOX15	ACCTTCCTGCTCGCCTAGTGTT	GGCTACAGAGAATGACGTTGGC
CA9	GTGCCTATGAGCAGTTGCTGTC	AAGTAGCGGCTGAAGTCAGAGG
TH	GCTGGACAAGTGTCATCACCTG	CCTGTACTGGAAGGCGATCTCA
HKDC1	ATCGCCGACTTCCTGGACTACA	GCCTTGAAACCTTTGGTCCACC
NDUFA4L2	CTGGGACAGAAAGAACAACCCG	CAGCCTGGCTTAGAAGTCTGGC
RRM2	CTGGCTCAAGAAACGAGGACTG	CTCTCCTCCGATGGTTTGTGTAC
ACSL5	GCTTATGAGCCCACTCCTGATG	GGAAGAATCCAACTCTGGCTCC
PLA2G4C	GGAAGACTGGTCAGAACTCACC	GCATTAGCAACAGCCCTTCTCC
LIPT2	GTCTGGCTAGACGATCGCAAGA	GCACGATGTGCTCAAACCACGT
UGDH	TGTGATGGTGCCCATGCTGTTG	GTCCATCGAAGATAAAGGCTGGC
FTCD	GGAGAACCTCTTCATCCTGGAG	ATGATCCGCTCCTTAGGGCTGA
ABAT	GCCTCTGATGAAGACGGAAGTC	CATTCGGTTGCCGTCCACATCA
AZIN2	CTTCACTGTGGCAGTCAGCATC	TCCCATACACGCCCTCATCAAG
HS6ST3	ACTGGACGGAGCTCACCAACTG	TCGCTCAGGTAACGTGACACTG
B4GALT6	CTCATTCCTTTCCGTAATCGCCA	GCCCACATTGAAAAGCATCGCAC
GUSB	CTGTCACCAAGAGCCAGTTCCT	GGTTGAAGTCCTTCACCAGCAG
DCT	CTCAGACCAACTTGGCTACAGC	CAACCAAAGCCACCAGTGTTCC
ALAS2	GCCTCAAAGGATGTGTCCGTCT	TACTGGTGCCTGAGATGTTGCG
MAT1A	GCCAAGTCTCTGGTGAAAGCAG	CTGTCTTCTGAGAGGTTCCGTAG
β-Actin	TGAATGATGAGCCTTCGTGC	CTGGTCTCAAGTCAGTGTAC
U6	CTCGCTTCGGCAGCACA	AACGCTTCACGAATTTGCGT

**Table 4:** List of 11 miRNA primers for real-time qPCR.

miRNA Name	miRNA Sequence (5′→3′)	Forward Sequence (5′→3′)
hsa-miR-1306-5p	CCACCTCCCCTGCAAACGTCCA	GCCACCTCCCCTGC
hsa-miR-627-3p	TCTTTTCTTTGAGACTCACT	CGCAGTCTTTTCTTTGAGACTC
hsa-miR-194-3p	CCAGTGGGGCTGCTGTTATCTG	CAGTGGGGCTGCTGT
hsa-miR-676-3p	CTGTCCTAAGGTTGTTGAGTT	CAGCTGTCCTAAGGTTGTTG
hsa-miR-6837-5p	ACCAGGGCCAGCAGGGAATGT	ACCAGGGCCAGCAG
hsa-miR-429	TAATACTGTCTGGTAAAACCGT	CGCAGTAATACTGTCTGGT
hsa-miR-100-3p	CAAGCTTGTATCTATAGGTATG	CGCAGCAAGCTTGTATC
hsa-miR-194-5p	CGGGTAGAGAGGGCAGTGGGAGG	CGGGTAGAGAGGGCAGT
hsa-miR-519a-3p	AAAGTGCATCCTTTTAGAGTGT	GCAGAAAGTGCATCCTTTTAGAG
hsa-miR-7-5p	TGGAAGACTAGTGATTTTGTTGTT	CGCAGTGGAAGACTAGTGA
hsa-miR-200a-5p	CATCTTACCGGACAGTGCTGGA	AGCATCTTACCGGACAGT

## Data Availability

The RNA and small RNA sequencing data were deposited in the NCBI Sequence Read Archive (accession numbers from SRR13675952 to SRR13675963).

## Supplementary Materials

The following are available online at https://www.mdpi.com/1422-0067/22/5/2292/s1: Table S1, Identification of 1037 differentially expressed mRNAs in response to naringenin; Table S2, Identification of 234 differentially expressed miRNAs in response to naringenin; Table S3, Identification of 5607 negative miRNA-mRNA pairs in response to naringenin, with the involvement of 216 DEMs and 681 DEGs in total.
