# Supplementary material for: The Effects of Naringenin on miRNA-mRNA Profiles in HepaRG Cells
Source: Int J Mol Sci. 2021 Feb 25;22(5):2292. doi: 10.3390/ijms22052292 (PMC7956767; doi:10.3390/ijms22052292)
Supplement: Supplementary file 1 [file ijms-22-02292-s001.pdf]

Supplementary Materials

Table S1. Identification of 1037 differentially expressed mRNAs in response to naringenin.

ID	Symbol	CK mean	T mean	Log2(FC)	P-value	FDR
ncbi_10628	TXNIP	127.8225	9.22	-3.79323	1.90E-177	3.20E-173
ncbi_9022	CLIC3	70.6475	16.1825	-2.1262	6.06E-142	5.09E-138
ncbi_91947	ARRDC4	15.9075	3.6575	-2.12078	8.72E-139	4.89E-135
ncbi_3005	H1F0	266.9375	69.04	-1.951	1.90E-134	7.98E-131
ncbi_250	ALPP	111.9975	22.575	-2.31067	2.86E-127	9.63E-124
ncbi_7134	TNNC1	39.985	9.3525	-2.09603	3.05E-113	8.56E-110
ncbi_112464	CAVIN3	77.4125	17.8	-2.12069	2.16E-104	5.19E-101
ncbi_85236	HIST1H2BK	445.075	153.9325	-1.53175	5.71E-91	1.20E-87
ncbi_7296	TXNRD1	97.99	224.5	1.196009	2.91E-90	5.44E-87
ncbi_716	C1S	45.9475	18.6475	-1.301	5.24E-89	8.82E-86
MSTRG.7959	GVQW1	20.3425	8.0325	-1.34058	8.93E-82	1.37E-78
ncbi_3875	KRT18	537.56	236.1275	-1.18686	1.67E-81	2.34E-78
ncbi_3006	HIST1H1C	50.585	13.765	-1.87771	6.36E-81	8.22E-78
ncbi_728113	ANXA8L1	38	10.085	-1.91379	2.76E-80	3.32E-77
ncbi_80201	HKDC1	42.6075	13.83	-1.62331	9.68E-79	1.09E-75
ncbi_2810	SFN	145.505	63.4	-1.19851	1.01E-70	1.06E-67
ncbi_4582	MUC1	129.055	46.0725	-1.48601	1.00E-63	9.39E-61
ncbi_6275	S100A4	456.0725	193.72	-1.23529	1.43E-63	1.27E-60
ncbi_8370	HIST2H4A	232.26	65.005	-1.83712	3.60E-63	3.03E-60
ncbi_554313	HIST2H4B	235.0325	65.12	-1.85169	3.94E-63	3.16E-60
ncbi_112268238	LOC112268238	18.435	5.6775	-1.69912	1.08E-61	8.23E-59
MSTRG.17054	ASMTL	0.8225	11.1725	3.763793	4.35E-60	3.18E-57
ncbi_23105	FSTL4	22.47	7.5175	-1.57968	1.13E-59	7.89E-57
ncbi_6241	RRM2	40.92	14.195	-1.52742	3.27E-59	2.20E-56
ncbi_1958	EGR1	2.845	0.3575	-2.99241	5.18E-58	3.35E-55
MSTRG.15726	GVQW1	1.67	0.26	-2.68326	5.96E-58	3.72E-55
ncbi_3725	JUN	14.555	5.2125	-1.48147	9.46E-58	5.69E-55
ncbi_30851	TAX1BP3	56.6025	25.31	-1.16116	7.71E-57	4.32E-54
ncbi_22861	NLRP1	8.6675	18.29	1.077367	5.12E-56	2.78E-53
ncbi_2244	FGB	13.65	43.9625	1.687372	8.05E-56	4.23E-53
MSTRG.8039	OR1F12	5.3025	13.34	1.331014	9.82E-55	5.01E-52
ncbi_27076	LYPD3	67.4275	26.6425	-1.33961	1.31E-53	6.49E-51
ncbi_11309	SLCO2B1	4.635	0.77	-2.58964	3.38E-53	1.62E-50
ncbi_5959	RDH5	14.49	4.305	-1.75097	5.98E-51	2.80E-48
ncbi_3856	KRT8	779.465	337.585	-1.20723	1.49E-50	6.76E-48
ncbi_251	ALPG	11.575	2.61	-2.14889	2.92E-50	1.29E-47
ncbi_653145	ANXA8	39.9675	10.9625	-1.86625	3.71E-50	1.60E-47
ncbi_415	ARSE	10.8925	4.5	-1.27534	2.24E-49	9.18E-47
ncbi_5971	RELB	11.2125	3.8075	-1.55819	7.11E-49	2.85E-46
ncbi_79919	MAB21L4	7.22	1.9225	-1.90902	1.65E-48	6.45E-46
MSTRG.3484	HERVK_113	2.325	7.6925	1.726222	2.09E-48	8.00E-46
ncbi_3914	LAMB3	105.435	50.4975	-1.06207	2.50E-47	9.34E-45
ncbi_117166	WFIKKN1	12.4225	4.015	-1.62948	2.77E-45	1.01E-42
ncbi_8351	HIST1H3D	17.355	3.31	-2.39045	3.72E-45	1.33E-42
ncbi_1844	DUSP2	12.4375	34.0725	1.453911	4.96E-45	1.74E-42
MSTRG.11707	C16orf89	2.6475	8.3525	1.657577	5.18E-44	1.78E-41
ncbi_2170	FABP3	18.75	5.98	-1.64867	1.38E-43	4.65E-41
ncbi_2521	FUS	184.075	82.66	-1.15503	1.01E-42	3.32E-40
ncbi_4648	MYO7B	0.9825	0.1575	-2.64111	1.39E-42	4.49E-40
ncbi_55742	PARVA	9.98	3.3675	-1.56736	1.67E-42	5.29E-40
ncbi_3484	IGFBP1	25.16	5.4025	-2.21943	4.26E-42	1.30E-39
ncbi_51435	SCARA3	45.295	17.57	-1.36624	6.00E-42	1.74E-39

ID	Symbol	CK mean	T mean	Log2(FC)	P-value	FDR
ncbi_83729	INHBE	3.93	1.0475	-1.90758	7.81E-42	2.23E-39
ncbi_3172	HNF4A	6.2225	1.82	-1.77356	9.25E-42	2.59E-39
ncbi_4640	MYO1A	4.53	1.415	-1.67871	1.01E-41	2.79E-39
ncbi_1890	TYMP	38.5825	17.13	-1.17142	3.46E-41	9.39E-39
ncbi_56834	GPR137	25.61	12.39	-1.04753	6.49E-41	1.71E-38
ncbi_138050	HGSNAT	16.7175	7.1625	-1.22282	1.09E-40	2.78E-38
ncbi_55765	INAVA	10.0025	3.94	-1.34409	2.34E-40	5.88E-38
ncbi_51330	TNFRSF12A	155.365	68.915	-1.17277	3.67E-40	9.07E-38
ncbi_26471	NUPR1	42.36	18.6325	-1.18488	7.06E-39	1.70E-36
MSTRG.3789	C16orf89	3.7	8.365	1.17684	1.37E-38	3.25E-36
MSTRG.12049	--	1.955	0.3375	-2.53421	2.55E-38	5.96E-36
ncbi_55062	WIPI1	5.565	13.635	1.292861	2.79E-38	6.44E-36
ncbi_246	ALOX15	1.9525	0.1775	-3.45943	5.48E-38	1.23E-35
ncbi_79817	MOB3B	4.7675	2.1225	-1.16747	5.70E-38	1.26E-35
ncbi_726	CAPN5	3.54	8.795	1.312934	1.63E-37	3.51E-35
ncbi_8839	CCN5	353.655	172.3275	-1.03719	2.05E-37	4.37E-35
ncbi_1464	CSPG4	47.4725	23.4675	-1.01643	2.81E-37	5.84E-35
MSTRG.5794	--	3.01	0.46	-2.71006	3.30E-37	6.69E-35
MSTRG.388	C16orf89	5.0275	14.1725	1.495181	6.29E-37	1.26E-34
ncbi_1490	CCN2	20.0175	9.3425	-1.09938	1.41E-36	2.76E-34
ncbi_5054	SERPINE1	60.685	18.715	-1.69714	3.66E-35	6.77E-33
ncbi_80737	VWA7	5.96	2.18	-1.45098	4.68E-35	8.56E-33
ncbi_4790	NFKB1	12.5175	6.115	-1.03352	5.07E-35	9.18E-33
ncbi_119395	CALHM3	4.31	1.0125	-2.08977	1.07E-34	1.88E-32
ncbi_105375355	UPK3B	4.78	1.275	-1.90651	1.74E-34	2.98E-32
ncbi_976	ADGRE5	55.785	27.37	-1.02728	3.68E-34	6.25E-32
ncbi_136647	MPLKIP	4.185	9.065	1.115079	9.57E-34	1.59E-31
ncbi_10381	TUBB3	90.4225	41.4375	-1.12574	1.52E-33	2.51E-31
ncbi_2707	GJB3	15.52	4.87	-1.67213	2.23E-33	3.64E-31
ncbi_7358	UGDH	37.4775	75.4375	1.009257	3.46E-33	5.59E-31
ncbi_79574	EPS8L3	25.775	11.11	-1.21411	5.90E-33	9.36E-31
ncbi_727897	MUC5B	1.0325	0.405	-1.35015	3.62E-32	5.58E-30
ncbi_100134444	KCNJ18	9.3525	2.675	-1.80581	4.25E-32	6.50E-30
ncbi_8971	H1FX	181.385	82.32	-1.13974	1.41E-31	2.14E-29
MSTRG.17131	CDC27	2.075	4.62	1.154782	1.55E-31	2.33E-29
ncbi_91156	IGFN1	0.8325	0.1725	-2.27085	2.68E-31	3.85E-29
ncbi_64005	MYO1G	4.9875	0.8925	-2.48239	3.99E-31	5.68E-29
ncbi_54625	PARP14	8.79	4.0225	-1.12777	5.38E-31	7.54E-29
ncbi_27242	TNFRSF21	6.29	14.74	1.228605	5.77E-31	8.02E-29
ncbi_4584	MUC3A	1.4225	0.5125	-1.4728	1.62E-30	2.22E-28
ncbi_1800	DPEP1	4.78	1.3725	-1.8002	1.63E-30	2.22E-28
ncbi_5540	NPY4R	7.4	2.6125	-1.50209	1.67E-30	2.26E-28
ncbi_7025	NR2F1	17.7175	35.4825	1.001933	1.14E-29	1.48E-27
MSTRG.8802	C10orf91	5.04	0.8425	-2.58068	1.22E-29	1.56E-27
MSTRG.12033	--	3.395	1.12	-1.59991	1.43E-29	1.82E-27
ncbi_143282	FGFBP3	1.735	5.8925	1.763944	1.50E-29	1.90E-27
ncbi_4502	MT2A	321.7725	148.64	-1.11422	1.60E-29	2.01E-27
ncbi_8764	TNFRSF14	3.3575	0.9775	-1.78022	2.32E-29	2.85E-27
ncbi_8635	RNASET2	5.765	12.8125	1.152159	7.10E-29	8.59E-27
ncbi_57101	ANO2	6.22	2.3675	-1.39355	1.02E-28	1.22E-26
ncbi_401934	RNF223	5.18	1.55	-1.74068	1.69E-28	1.99E-26
ncbi_8970	HIST1H2BJ	22.225	4.43	-2.3268	1.84E-28	2.15E-26
ncbi_10148	EBI3	10.9375	4.2025	-1.37996	1.93E-28	2.24E-26
ncbi_3014	H2AFX	73.9725	35.9775	-1.03989	4.32E-28	4.87E-26

ID	Symbol	CK mean	T mean	Log2(FC)	P-value	FDR
ncbi_1634	DCN	8.1	3.1275	-1.37291	4.90E-28	5.46E-26
ncbi_128209	KLF17	3.285	0.985	-1.7377	5.49E-28	6.07E-26
ncbi_6578	SLCO2A1	7.7225	2.9075	-1.40929	6.26E-28	6.88E-26
ncbi_1396	CRIP1	334.8025	147.5075	-1.18252	8.95E-28	9.59E-26
ncbi_64699	TMPRSS3	21.5025	7.8475	-1.4542	9.39E-28	1.00E-25
ncbi_10158	PDZK1IP1	31.9	13.6075	-1.22915	2.03E-27	2.10E-25
ncbi_8337	HIST2H2AA3	58.9675	22.8775	-1.36599	2.04E-27	2.10E-25
ncbi_9415	FADS2	17.0025	7.4675	-1.18705	2.74E-27	2.75E-25
ncbi_79674	VEPH1	3.5025	1.345	-1.38078	2.79E-27	2.77E-25
ncbi_25956	SEC31B	1.94	4.535	1.225046	3.95E-27	3.91E-25
ncbi_723790	HIST2H2AA4	59.0825	22.9675	-1.36314	4.77E-27	4.69E-25
ncbi_10893	MMP24	5.105	1.1275	-2.17878	7.42E-27	7.26E-25
MSTRG.5538	C6orf223	22.295	10.905	-1.03173	8.59E-27	8.35E-25
ncbi_2200	FBN1	68.465	32.055	-1.09482	1.24E-26	1.19E-24
ncbi_6536	SLC6A9	46.3925	17.67	-1.39259	1.60E-26	1.52E-24
ncbi_80032	ZNF556	1.1925	3.36	1.494472	2.78E-26	2.64E-24
ncbi_8479	HIRIP3	26.925	13.2425	-1.02377	3.73E-26	3.51E-24
ncbi_1307	COL16A1	2.8275	0.87	-1.70044	4.00E-26	3.74E-24
ncbi_112694756	LOC112694756	54.95	22.645	-1.27893	4.43E-26	4.12E-24
ncbi_5159	PDGFRB	3.8775	1.6175	-1.26136	7.53E-26	6.88E-24
ncbi_55179	FAIM	14.355	6.955	-1.04543	7.74E-26	7.04E-24
ncbi_171425	CLYBL	4.8525	12.4475	1.359056	3.49E-25	3.04E-23
ncbi_55244	SLC47A1	2.9425	6.8625	1.221692	7.00E-25	6.01E-23
ncbi_8334	HIST1H2AC	84.2	36.0775	-1.22272	8.00E-25	6.76E-23
ncbi_84962	AJUBA	42.395	19.2	-1.14279	1.60E-24	1.32E-22
MSTRG.12023	Pol	1.4925	0.33	-2.17719	1.89E-24	1.56E-22
ncbi_4261	CIITA	2.42	0.7225	-1.74394	3.34E-24	2.70E-22
ncbi_3017	HIST1H2BD	41.3275	17.2625	-1.25946	3.59E-24	2.88E-22
MSTRG.4471	Pol	4.1475	1.9075	-1.12056	4.20E-24	3.35E-22
ncbi_147463	ANKRD29	0.85	2.3175	1.447035	4.41E-24	3.50E-22
ncbi_2247	FGF2	8.4675	3.985	-1.08736	7.56E-24	5.76E-22
ncbi_55577	NAGK	13.09	6.0975	-1.10218	9.31E-24	7.02E-22
ncbi_144132	DNHD1	5.43	12.4025	1.191607	1.04E-23	7.80E-22
ncbi_1893	ECM1	18.4575	8.7575	-1.07562	1.10E-23	8.16E-22
ncbi_8076	MFAP5	5.7575	1.855	-1.63402	1.16E-23	8.49E-22
ncbi_8329	HIST1H2AI	13.8175	4.2675	-1.69503	1.17E-23	8.58E-22
ncbi_1436	CSF1R	1.8225	0.38	-2.26185	1.38E-23	1.00E-21
ncbi_5328	PLAU	3.8275	1.065	-1.84555	1.40E-23	1.01E-21
MSTRG.5151	--	4.9975	1.33	-1.90978	2.53E-23	1.80E-21
ncbi_3561	IL2RG	3.195	0.6	-2.41278	2.83E-23	2.01E-21
ncbi_9770	RASSF2	2.7025	1.085	-1.3166	3.37E-23	2.36E-21
ncbi_23371	TNS2	12.045	5.5975	-1.10558	3.47E-23	2.42E-21
ncbi_8969	HIST1H2AG	32.9525	12.44	-1.4054	3.78E-23	2.63E-21
ncbi_56704	JPH1	6.145	12.3475	1.006734	6.41E-23	4.38E-21
ncbi_10274	STAG1	9.495	4.6525	-1.02916	6.75E-23	4.60E-21
ncbi_53841	CDHR5	5.5775	2.52	-1.14619	6.96E-23	4.72E-21
ncbi_51626	DYNC2LI1	2.91	6.9925	1.264789	7.28E-23	4.92E-21
ncbi_7837	PXDN	146.7925	72.7975	-1.01182	1.18E-22	7.82E-21
ncbi_26040	SETBP1	0.975	0.36	-1.43741	1.40E-22	9.25E-21
ncbi_101928841	LOC101928841	4.1025	1.87	-1.13347	1.53E-22	9.93E-21
ncbi_55561	CDC42BPG	4.98	2.4675	-1.0131	1.90E-22	1.23E-20
ncbi_343990	KIAA1211L	3.725	1.845	-1.01362	2.52E-22	1.62E-20
ncbi_7185	TRAF1	1.795	0.51	-1.81541	3.16E-22	2.01E-20
ncbi_5271	SERPINB8	2.38	0.735	-1.69515	3.84E-22	2.42E-20

ID	Symbol	CK mean	T mean	Log2(FC)	P-value	FDR
ncbi_136306	SVOPL	0.8725	0.135	-2.6922	5.42E-22	3.39E-20
MSTRG.5945	--	2.8125	5.8675	1.060893	6.58E-22	4.10E-20
ncbi_1188	CLCNKB	1.8375	0.435	-2.07866	6.91E-22	4.29E-20
MSTRG.7199	ASCC1	3.4075	1.2425	-1.45547	1.07E-21	6.56E-20
ncbi_440603	BCL2L15	0.8425	0.11	-2.93717	1.17E-21	7.09E-20
ncbi_1410	CRYAB	7.77	0.995	-2.96515	1.26E-21	7.57E-20
MSTRG.8615	NEK4	3.5	7.4875	1.097129	1.47E-21	8.76E-20
MSTRG.12028	BEND2	1.3075	2.955	1.176347	1.73E-21	1.03E-19
ncbi_6273	S100A2	9.6225	3.295	-1.54613	1.82E-21	1.08E-19
MSTRG.819	GVQW1	3.5175	8.43	1.260982	2.32E-21	1.36E-19
ncbi_7433	VIPR1	1.7325	0.39	-2.15131	2.49E-21	1.45E-19
MSTRG.3471	C16orf89	0.7425	2.085	1.489584	5.33E-21	2.98E-19
ncbi_100996758	NPY4R2	4.2325	1.4925	-1.50378	6.30E-21	3.47E-19
ncbi_8581	LY6D	15.5725	6.2175	-1.32459	6.43E-21	3.53E-19
ncbi_4188	MDFI	2.7	0.71	-1.92707	6.45E-21	3.54E-19
ncbi_3691	ITGB4	43.685	17.2725	-1.33866	6.69E-21	3.66E-19
ncbi_79987	SVEP1	1.3425	0.57	-1.23589	6.75E-21	3.67E-19
ncbi_7430	EZR	96.5275	46.73	-1.04659	6.79E-21	3.68E-19
ncbi_9980	DOP1B	0.695	1.7225	1.309419	7.05E-21	3.81E-19
ncbi_100506127	GVQW3	4.245	8.785	1.049278	8.77E-21	4.70E-19
ncbi_4222	MEOX1	3.7975	1.2625	-1.58877	8.99E-21	4.80E-19
ncbi_126961	HIST2H3C	7.99	1.9925	-2.00362	1.06E-20	5.61E-19
ncbi_81888	HYI	9.0675	3.88	-1.22465	1.16E-20	6.12E-19
MSTRG.16145	--	1.4	3.715	1.407935	1.29E-20	6.73E-19
ncbi_3773	KCNJ16	4.26	1.28	-1.73471	2.51E-20	1.26E-18
ncbi_728392	LOC728392	3.595	9.5675	1.41215	2.62E-20	1.31E-18
ncbi_333932	HIST2H3A	7.7775	1.96	-1.98845	3.50E-20	1.73E-18
ncbi_7087	ICAM5	12.17	24.8075	1.027447	4.68E-20	2.30E-18
ncbi_55057	CRYBG2	2.3275	0.7025	-1.72821	8.78E-20	4.15E-18
ncbi_126868	MAB21L3	2.63	0.7	-1.90964	1.42E-19	6.62E-18
MSTRG.1618	Pol	3.37	8.3325	1.306001	1.92E-19	8.84E-18
ncbi_6531	SLC6A3	1.38	0.395	-1.80474	2.08E-19	9.57E-18
ncbi_8372	HYAL3	25.6975	12.8	-1.00548	2.82E-19	1.29E-17
ncbi_7161	TP73	1.7725	0.69	-1.36112	3.67E-19	1.66E-17
ncbi_56901	NDUFA4L2	36.48	12.5025	-1.54489	4.10E-19	1.83E-17
MSTRG.1860	GVQW1	3.485	1.6125	-1.11186	7.50E-19	3.27E-17
ncbi_6517	SLC2A4	0.5625	1.9375	1.784271	7.88E-19	3.41E-17
ncbi_340061	TMEM173	3.3475	0.88	-1.92751	8.50E-19	3.65E-17
ncbi_55876	GSDMB	2.1375	5.2	1.282587	9.24E-19	3.94E-17
ncbi_5672	PSG4	1.805	0.4775	-1.91843	9.79E-19	4.15E-17
MSTRG.5604	BEND2	1.18	3.4425	1.54467	1.29E-18	5.38E-17
MSTRG.8641	C16orf89	1.3125	2.9875	1.186621	1.32E-18	5.47E-17
ncbi_114897	C1QTNF1	2.8125	1.0025	-1.48825	1.37E-18	5.66E-17
ncbi_81493	SYNC	8.3225	4.02	-1.04982	1.97E-18	8.08E-17
ncbi_58476	TP53INP2	4.605	1.9875	-1.21225	2.22E-18	9.07E-17
ncbi_4739	NEDD9	6.2825	2.435	-1.36742	2.55E-18	1.03E-16
ncbi_6585	SLIT1	0.8925	0.21	-2.08746	3.28E-18	1.32E-16
MSTRG.14179	--	3.91	9.8	1.325613	3.56E-18	1.42E-16
ncbi_54344	DPM3	34.58	75.3125	1.122951	5.63E-18	2.20E-16
ncbi_1308	COL17A1	1.095	0.2875	-1.9293	6.93E-18	2.67E-16
ncbi_7473	WNT3	3.4425	1.645	-1.06537	7.26E-18	2.79E-16
ncbi_8857	FCGBP	0.1075	0.4125	1.940057	1.33E-17	5.05E-16
ncbi_161753	ODF3L1	2.7825	0.7475	-1.89624	1.43E-17	5.36E-16
ncbi_195814	SDR16C5	2.085	0.7675	-1.44181	1.71E-17	6.37E-16

ID	Symbol	CK mean	T mean	Log2(FC)	P-value	FDR
ncbi_8638	OASL	2.6075	0.56	-2.21917	1.80E-17	6.71E-16
MSTRG.17600	CDR2	3.125	7.7525	1.310805	1.81E-17	6.73E-16
ncbi_4773	NFATC2	1.66	0.705	-1.23549	2.75E-17	1.01E-15
ncbi_84449	ZNF333	0.6325	1.51	1.255411	2.83E-17	1.03E-15
ncbi_8341	HIST1H2BN	22.16	10.675	-1.05372	3.46E-17	1.25E-15
ncbi_389813	AJM1	9.56	4.3925	-1.12197	3.50E-17	1.26E-15
ncbi_10071	MUC12	0.285	0.055	-2.37346	3.97E-17	1.42E-15
MSTRG.5107	--	3.295	1.5625	-1.07642	5.64E-17	2.01E-15
ncbi_127294	MYOM3	0.7575	0.2175	-1.80023	6.08E-17	2.15E-15
ncbi_79628	SH3TC2	0.1525	0.045	-1.76081	7.89E-17	2.75E-15
ncbi_2044	EPHA5	0.555	0.1625	-1.77205	9.96E-17	3.43E-15
ncbi_196394	AMN1	1.1125	2.685	1.271117	1.00E-16	3.44E-15
ncbi_8347	HIST1H2BC	13.3775	3.995	-1.74354	1.27E-16	4.32E-15
ncbi_57535	KIAA1324	1.67	0.6025	-1.47081	1.35E-16	4.59E-15
ncbi_5029	P2RY2	3.6125	1.495	-1.27285	1.39E-16	4.70E-15
MSTRG.2243	--	0.4425	1.255	1.503938	1.49E-16	5.03E-15
MSTRG.8072	--	4.5475	9.4075	1.048738	1.83E-16	6.12E-15
MSTRG.1715	UTY	3.255	7.4525	1.195067	1.94E-16	6.46E-15
ncbi_322	APBB1	3.07	1.15	-1.4166	2.95E-16	9.59E-15
MSTRG.16447	--	1.565	0.48	-1.70506	3.05E-16	9.87E-15
MSTRG.8690	ZNF91	2.4275	7.555	1.63796	3.06E-16	9.87E-15
MSTRG.17348	CDC27	1.0075	2.17	1.106915	4.45E-16	1.41E-14
MSTRG.10892	--	1.8325	4.27	1.220423	7.39E-16	2.27E-14
MSTRG.16424	UTY	2.6675	5.8025	1.121186	8.68E-16	2.65E-14
ncbi_5967	REG1A	7.0175	2.105	-1.73714	1.30E-15	3.93E-14
ncbi_3689	ITGB2	3.6375	1.465	-1.31205	1.55E-15	4.62E-14
ncbi_9134	CCNE2	6.925	3.3525	-1.04658	1.79E-15	5.31E-14
ncbi_2859	GPR35	2.2975	0.74	-1.63447	2.03E-15	5.99E-14
MSTRG.17267	ZBTB12	2.5225	0.985	-1.35666	2.90E-15	8.36E-14
ncbi_478	ATP1A3	1.9675	4.48	1.187135	3.59E-15	1.02E-13
ncbi_3486	IGFBP3	84.8475	31.675	-1.42153	4.62E-15	1.29E-13
ncbi_4815	NINJ2	10.945	5.465	-1.00198	4.70E-15	1.31E-13
MSTRG.16	C16orf89	1.575	4.4	1.482152	4.93E-15	1.37E-13
ncbi_102724398	CH507-42P11.6	2.1275	1.055	-1.01192	5.90E-15	1.63E-13
ncbi_25789	TMEM59L	3.5225	8.7525	1.313095	6.12E-15	1.69E-13
ncbi_9542	NRG2	2.3525	0.9925	-1.24506	6.29E-15	1.72E-13
ncbi_183	AGT	7.37	3.39	-1.12038	7.14E-15	1.95E-13
ncbi_493861	EID3	4.0675	1.475	-1.46343	9.14E-15	2.46E-13
ncbi_4917	NTN3	3.295	1.1425	-1.52808	2.18E-14	5.69E-13
ncbi_84253	GARNL3	1.275	3.19	1.323059	2.25E-14	5.84E-13
ncbi_7448	VTN	7.73	3.3425	-1.20954	3.02E-14	7.79E-13
ncbi_140453	MUC17	0.1125	0.001	-6.81378	3.53E-14	9.04E-13
MSTRG.14022	GVQW1	4.825	2.055	-1.23139	4.06E-14	1.04E-12
ncbi_27289	RND1	5.8675	2.765	-1.08547	4.40E-14	1.12E-12
ncbi_53615	MBD3	9.2475	3.6225	-1.35208	4.52E-14	1.14E-12
ncbi_3242	HPD	29.545	14.5925	-1.01769	5.21E-14	1.32E-12
ncbi_23213	SULF1	0.9175	1.96	1.095074	6.05E-14	1.51E-12
ncbi_60676	PAPPA2	7.9725	3.03	-1.39571	7.28E-14	1.82E-12
ncbi_9915	ARNT2	0.63	0.19	-1.72935	7.35E-14	1.83E-12
ncbi_25858	CATSPERZ	3.2325	0.88	-1.87707	7.36E-14	1.83E-12
ncbi_7071	KLF10	46.9575	22.4325	-1.06577	7.40E-14	1.84E-12
ncbi_51421	AMOTL2	23.455	11.1225	-1.07641	8.14E-14	2.00E-12
MSTRG.9524	GVQW1	10.295	21.17	1.040077	8.55E-14	2.10E-12
ncbi_388650	DIPK1A	0.8175	2.4125	1.561238	1.03E-13	2.52E-12

ID	Symbol	CK mean	T mean	Log2(FC)	P-value	FDR
ncbi_6518	SLC2A5	0.5325	0.1075	-2.30844	1.10E-13	2.68E-12
ncbi_10060	ABCC9	0.53	0.1875	-1.4991	1.17E-13	2.82E-12
ncbi_339488	TFAP2E	0.6525	1.6075	1.300769	1.34E-13	3.21E-12
ncbi_401265	KLHL31	0.5975	1.4275	1.25648	1.35E-13	3.23E-12
ncbi_3669	ISG20	3.7325	1.3825	-1.43286	1.41E-13	3.36E-12
ncbi_4778	NFE2	7.03	3.38	-1.0565	1.91E-13	4.45E-12
ncbi_10659	CELF2	3.26	1.4	-1.21945	2.11E-13	4.88E-12
MSTRG.14771	C16orf89	0.4175	1.77	2.083901	2.70E-13	6.15E-12
ncbi_1907	EDN2	9.4875	31.385	1.725975	2.97E-13	6.72E-12
MSTRG.16105	--	2.68	9.1725	1.775082	3.12E-13	7.04E-12
ncbi_10752	CHL1	2.6825	1.32	-1.02304	3.13E-13	7.06E-12
MSTRG.293	AGBL3	1.1175	2.625	1.232043	3.72E-13	8.33E-12
ncbi_9658	ZNF516	0.775	1.5675	1.016197	4.06E-13	9.03E-12
ncbi_25903	OLFML2B	2.505	1.16	-1.11069	4.14E-13	9.18E-12
ncbi_57530	CGN	1.755	0.875	-1.00412	4.30E-13	9.54E-12
ncbi_7051	TGM1	2.2175	0.9	-1.30094	5.37E-13	1.18E-11
ncbi_285489	DOK7	4.465	1.3625	-1.7124	5.86E-13	1.28E-11
MSTRG.7307	--	5.14	11.3575	1.143805	6.65E-13	1.45E-11
ncbi_65266	WNK4	0.82	1.8525	1.175778	7.56E-13	1.63E-11
MSTRG.8052	C16orf89	1.335	2.835	1.086509	8.02E-13	1.72E-11
ncbi_3866	KRT15	1.2375	0.305	-2.02055	1.40E-12	2.91E-11
ncbi_5349	FXYD3	5.5025	2.1425	-1.36079	1.40E-12	2.91E-11
ncbi_8456	FOXN1	10.385	4.5275	-1.19771	1.81E-12	3.71E-11
ncbi_110116772	ZNF765-ZNF761	0.3625	1.225	1.756729	1.89E-12	3.86E-11
ncbi_2709	GJB5	3.39	1.24	-1.45095	2.63E-12	5.27E-11
ncbi_83547	RILP	9.5875	4.745	-1.01475	2.71E-12	5.42E-11
ncbi_54587	MXRA8	3.12	1.485	-1.07108	3.16E-12	6.26E-11
ncbi_5655	KLK10	0.4725	0.001	-8.88417	3.54E-12	6.99E-11
MSTRG.6100	--	11.74	24.3575	1.052934	3.68E-12	7.26E-11
MSTRG.1023	GNRHR2	2.3175	4.9925	1.107193	3.89E-12	7.66E-11
ncbi_8339	HIST1H2BG	12.64	4.725	-1.41961	4.25E-12	8.31E-11
ncbi_91752	ZNF804A	0.965	0.3875	-1.31633	4.61E-12	8.96E-11
ncbi_80274	SCUBE1	2.41	1.1175	-1.10876	5.53E-12	1.07E-10
ncbi_102724488	LOC102724488	0.59	1.2175	1.045135	5.65E-12	1.09E-10
MSTRG.6662	GVQW2	1.035	2.1725	1.069725	5.72E-12	1.10E-10
ncbi_51599	LSR	2.295	1.0375	-1.14538	5.85E-12	1.12E-10
ncbi_83690	CRISPLD1	2.5175	1.24	-1.02165	7.43E-12	1.41E-10
ncbi_7471	WNT1	1.15	0.325	-1.82312	9.09E-12	1.71E-10
ncbi_23237	ARC	2.655	1.305	-1.02466	9.10E-12	1.71E-10
ncbi_50617	ATP6V0A4	1.23	0.3525	-1.80296	9.18E-12	1.72E-10
MSTRG.1859	GVQW1	0.6875	0.1225	-2.48858	1.32E-11	2.45E-10
MSTRG.5663	--	1.78	0.8525	-1.06211	1.49E-11	2.75E-10
ncbi_4015	LOX	17.72	8.3425	-1.08683	1.62E-11	2.98E-10
ncbi_127707	KLHDC7A	0.7525	0.195	-1.94822	1.74E-11	3.18E-10
MSTRG.294	NEK4	0.4375	1.34	1.614878	1.76E-11	3.22E-10
MSTRG.9455	--	1.59	5.21	1.712257	1.78E-11	3.24E-10
ncbi_257019	FRMD3	1.0075	2.265	1.168731	1.89E-11	3.42E-10
MSTRG.1636	--	1.0975	2.64	1.266317	2.14E-11	3.86E-10
ncbi_3352	HTR1D	0.83	0.21	-1.98272	2.20E-11	3.95E-10
ncbi_342897	NCCRP1	5.385	2.45	-1.13616	2.40E-11	4.30E-10
ncbi_79098	C1orf116	0.6525	0.2275	-1.52011	2.47E-11	4.42E-10
MSTRG.13914	C16orf89	1.84	3.975	1.111249	2.63E-11	4.69E-10
ncbi_6932	TCF7	1.2075	0.3375	-1.83906	2.98E-11	5.26E-10
ncbi_256158	HMCN2	0.56	0.275	-1.026	3.31E-11	5.81E-10

ID	Symbol	CK mean	T mean	Log2(FC)	P-value	FDR
MSTRG.11160	--	2.275	1.035	-1.13624	3.64E-11	6.38E-10
MSTRG.14247	--	2.6075	5.9275	1.184757	4.13E-11	7.16E-10
MSTRG.17484	PPP2R3B	0.415	2.405	2.534854	4.22E-11	7.30E-10
ncbi_80345	ZSCAN16	2.7875	1.265	-1.13983	4.64E-11	7.95E-10
MSTRG.13971	GVQW1	1.9425	4.7625	1.293804	6.18E-11	1.05E-09
ncbi_5919	RARRES2	6.5525	2.7575	-1.24868	6.36E-11	1.07E-09
ncbi_285966	TCAF2	2.1375	0.8775	-1.28445	6.43E-11	1.08E-09
MSTRG.5177	--	4.235	1.4975	-1.49981	7.66E-11	1.28E-09
ncbi_285755	PPIL6	0.6975	1.87	1.422773	1.16E-10	1.90E-09
ncbi_375791	CYSRT1	16.0925	6.965	-1.20819	1.27E-10	2.07E-09
ncbi_284422	SMIM24	1.4375	0.28	-2.36006	1.28E-10	2.08E-09
ncbi_55586	MIOX	0.13	1.6225	3.641635	1.50E-10	2.40E-09
ncbi_3037	HAS2	0.63	0.18	-1.80735	1.61E-10	2.57E-09
MSTRG.10700	ERVK-21	1.6325	3.5525	1.121752	1.73E-10	2.75E-09
MSTRG.9406	C16orf89	1.475	2.9925	1.020636	1.92E-10	3.05E-09
ncbi_5239	PGM5	1.9475	0.95	-1.03562	1.95E-10	3.09E-09
MSTRG.5539	C6orf223	16.55	8.1875	-1.01534	2.00E-10	3.16E-09
MSTRG.10470	ZNF91	2.1725	4.7	1.113305	2.41E-10	3.78E-09
ncbi_23349	PHF24	0.125	0.4875	1.963474	2.59E-10	4.03E-09
ncbi_146547	PRSS36	1.6775	0.7875	-1.09096	2.62E-10	4.06E-09
ncbi_102800317	TPTEP2-CSNK1E	6.725	0.415	-4.01835	2.63E-10	4.07E-09
ncbi_107984590	LOC107984590	0.9675	0.3025	-1.67733	3.04E-10	4.65E-09
MSTRG.4349	C16orf89	1.45	2.9725	1.035624	3.04E-10	4.65E-09
ncbi_2562	GABRB3	0.525	0.175	-1.58496	3.24E-10	4.93E-09
ncbi_90226	UCN2	2.3425	0.52	-2.17147	3.51E-10	5.32E-09
ncbi_3371	TNC	0.3725	0.1025	-1.86162	3.73E-10	5.61E-09
ncbi_285346	ZNF852	1.2175	2.6825	1.139656	5.92E-10	8.68E-09
ncbi_88	ACTN2	0.2525	0.025	-3.33628	6.31E-10	9.22E-09
ncbi_3108	HLA-DMA	3.9425	1.595	-1.30555	6.56E-10	9.55E-09
ncbi_6690	SPINK1	4.2175	1.48	-1.51079	6.69E-10	9.71E-09
ncbi_64284	RAB17	2.3575	0.8875	-1.40944	6.75E-10	9.79E-09
ncbi_8605	PLA2G4C	1.1025	0.4425	-1.31703	7.28E-10	1.05E-08
ncbi_63901	FAM111A	2.09	1.02	-1.03493	7.55E-10	1.08E-08
ncbi_64856	VWA1	0.755	0.26	-1.53797	9.40E-10	1.33E-08
ncbi_130888	FBXO36	0.68	1.53	1.169925	1.17E-09	1.63E-08
ncbi_376267	RAB15	0.9525	0.3275	-1.54022	1.20E-09	1.67E-08
ncbi_8355	HIST1H3G	12.3425	5.735	-1.10577	1.48E-09	2.04E-08
ncbi_57462	MYORG	2.47	1.2175	-1.02059	1.53E-09	2.10E-08
ncbi_1298	COL9A2	0.515	0.07	-2.87915	1.65E-09	2.24E-08
MSTRG.5254	NEK4	0.5725	1.6325	1.511735	1.69E-09	2.30E-08
ncbi_10129	FRY	0.38	0.135	-1.49304	1.71E-09	2.32E-08
ncbi_8835	SOCS2	2.2225	1.0075	-1.1414	1.84E-09	2.49E-08
ncbi_492311	IGIP	0.8775	1.78	1.020406	2.24E-09	2.99E-08
MSTRG.16618	--	3.245	6.8125	1.069966	2.27E-09	3.02E-08
ncbi_834	CASP1	2.5725	1.0125	-1.34525	2.63E-09	3.48E-08
MSTRG.13706	--	0.87	3.36	1.949374	2.66E-09	3.51E-08
ncbi_768	CA9	31.3875	7.47	-2.07101	2.96E-09	3.87E-08
ncbi_5137	PDE1C	0.8775	0.4275	-1.03747	3.02E-09	3.94E-08
MSTRG.17384	CDHR5	2.2	0.8425	-1.38475	3.54E-09	4.55E-08
ncbi_275	AMT	1.06	2.4625	1.216059	3.64E-09	4.67E-08
ncbi_401397	SMIM30	17.2075	36.6825	1.092054	3.90E-09	4.95E-08
MSTRG.14037	--	1.2325	2.78	1.173497	4.05E-09	5.13E-08
MSTRG.15714	GVQW2	4.725	10.215	1.112303	4.06E-09	5.14E-08
ncbi_55840	EAF2	1.45	3.0925	1.092721	4.25E-09	5.37E-08

ID	Symbol	CK mean	T mean	Log2(FC)	P-value	FDR
ncbi_171024	SYNPO2	0.285	0.0875	-1.70361	4.26E-09	5.37E-08
ncbi_7066	THPO	2.455	1.1875	-1.0478	4.33E-09	5.45E-08
ncbi_57571	CARNS1	1.4975	0.5475	-1.45163	4.42E-09	5.55E-08
ncbi_1435	CSF1	3.5875	1.69	-1.08596	4.43E-09	5.57E-08
ncbi_8365	HIST1H4H	10.7975	4.2825	-1.33417	4.54E-09	5.68E-08
ncbi_8291	DYSF	0.8425	0.4	-1.07468	4.71E-09	5.87E-08
ncbi_283298	OLFML1	0.975	0.0925	-3.39788	5.02E-09	6.21E-08
ncbi_339210	C17orf67	0.8325	1.975	1.24633	5.03E-09	6.22E-08
ncbi_283229	CRACR2B	2.62	1.1225	-1.22285	5.09E-09	6.28E-08
ncbi_79173	C19orf57	1.73	0.8375	-1.04661	5.28E-09	6.50E-08
ncbi_89870	TRIM15	2.4175	1.1475	-1.07502	5.36E-09	6.58E-08
ncbi_9050	PSTPIP2	0.855	2.21	1.37005	5.41E-09	6.64E-08
MSTRG.11524	HERC2	1.37	2.7675	1.014407	5.59E-09	6.84E-08
ncbi_4868	NPHS1	0.7575	0.32	-1.24317	5.85E-09	7.12E-08
ncbi_221336	BEND6	0.865	1.9275	1.155959	6.39E-09	7.72E-08
MSTRG.2028	--	3.4625	1.4725	-1.23355	7.84E-09	9.37E-08
ncbi_8343	HIST1H2BF	5.64	1.6875	-1.74081	8.42E-09	1.00E-07
ncbi_1087	CEACAM7	1.4975	0.505	-1.5682	9.81E-09	1.16E-07
MSTRG.15881	CDC27	3.095	6.4025	1.048696	1.01E-08	1.18E-07
ncbi_2920	CXCL2	1.855	0.6275	-1.56373	1.13E-08	1.31E-07
ncbi_105371409	LOC105371409	3.6275	1.7075	-1.08709	1.19E-08	1.38E-07
ncbi_7462	LAT2	0.9875	0.2925	-1.75534	1.24E-08	1.43E-07
MSTRG.5936	UTY	3.33	0.97	-1.77947	1.29E-08	1.49E-07
ncbi_1755	DMBT1	0.3825	0.1075	-1.83112	1.51E-08	1.72E-07
MSTRG.13027	ERVK-25	0.76	1.7	1.161463	1.54E-08	1.76E-07
ncbi_6947	TCN1	1.1725	0.2475	-2.24409	1.74E-08	1.97E-07
ncbi_8942	KYNU	0.41	0.1725	-1.24903	1.93E-08	2.17E-07
ncbi_5047	PAEP	2.235	0.755	-1.56573	2.23E-08	2.47E-07
ncbi_84142	ABRAXAS1	0.7725	1.64	1.086089	2.39E-08	2.65E-07
ncbi_100129484	LOC100129484	1.58	0.73	-1.11396	2.47E-08	2.73E-07
ncbi_58189	WFDC1	23.255	11.6025	-1.00311	2.64E-08	2.89E-07
ncbi_284297	SSC5D	0.6375	0.2225	-1.51862	2.88E-08	3.14E-07
MSTRG.13745	C16orf89	1.0425	3.02	1.534501	3.16E-08	3.43E-07
ncbi_84561	SLC12A8	0.2925	0.795	1.442518	3.30E-08	3.56E-07
MSTRG.16377	SERHL2	0.2425	1.57	2.694708	3.33E-08	3.59E-07
ncbi_388595	TMEM82	0.7475	0.1275	-2.55158	3.49E-08	3.75E-07
MSTRG.10628	REL	0.7	2.77	1.984459	3.53E-08	3.78E-07
ncbi_25759	SHC2	1.8475	0.7	-1.40015	3.65E-08	3.91E-07
ncbi_6274	S100A3	5.7525	2.3225	-1.30851	3.88E-08	4.15E-07
MSTRG.17058	--	1.4275	3.45	1.273106	4.04E-08	4.31E-07
ncbi_30811	HUNK	0.4	0.1425	-1.48904	4.20E-08	4.47E-07
ncbi_91749	MFSD4B	1.035	2.095	1.017319	4.40E-08	4.66E-07
MSTRG.15	Pol	0.3475	1.1925	1.778904	4.53E-08	4.79E-07
ncbi_26261	FBXO24	0.65	1.5325	1.237375	5.23E-08	5.44E-07
ncbi_120939	TMEM52B	1.535	0.7525	-1.02848	5.30E-08	5.51E-07
ncbi_6556	SLC11A1	0.8525	0.39	-1.12823	5.36E-08	5.57E-07
MSTRG.11431	--	1.26	2.96	1.232173	5.45E-08	5.65E-07
MSTRG.16493	--	2.26	4.565	1.014292	5.47E-08	5.67E-07
ncbi_90427	BMF	1.3225	0.6	-1.14023	5.74E-08	5.90E-07
MSTRG.6270	C16orf89	0.94	2.25	1.259192	6.23E-08	6.38E-07
ncbi_9507	ADAMTS4	0.2125	0.035	-2.60204	6.32E-08	6.47E-07
ncbi_27293	SMPDL3B	2.06	0.945	-1.12426	6.99E-08	7.10E-07
MSTRG.13939	UTY	1.8575	0.9075	-1.03339	7.12E-08	7.22E-07
MSTRG.3860	--	2.615	5.51	1.075241	8.19E-08	8.19E-07

ID	Symbol	CK mean	T mean	Log2(FC)	P-value	FDR
ncbi_5652	PRSS8	0.655	0.17	-1.94596	8.45E-08	8.44E-07
ncbi_2634	GBP2	0.21	0.585	1.478047	8.56E-08	8.52E-07
MSTRG.11145	--	2.5325	0.8325	-1.60504	8.78E-08	8.72E-07
MSTRG.1701	C16orf89	0.805	2.2025	1.452081	9.90E-08	9.72E-07
ncbi_342667	STAC2	0.9125	0.325	-1.48938	9.92E-08	9.74E-07
ncbi_5570	PKIB	5.51	2.2225	-1.30987	1.02E-07	9.99E-07
ncbi_5327	PLAT	1.3625	0.5425	-1.32856	1.02E-07	9.99E-07
ncbi_51062	ATL1	1.2625	0.5225	-1.27278	1.03E-07	1.01E-06
MSTRG.4344	--	2.2175	0.8575	-1.37073	1.19E-07	1.15E-06
ncbi_202	CRYBG1	0.3225	0.1175	-1.45664	1.21E-07	1.17E-06
ncbi_387787	LIPT2	1.48	0.6525	-1.18155	1.30E-07	1.25E-06
ncbi_1645	AKR1C1	0.62	1.2775	1.042983	1.30E-07	1.25E-06
ncbi_8357	HIST1H3H	12.7775	5.625	-1.18368	1.44E-07	1.38E-06
ncbi_4622	MYH4	0.175	0.035	-2.32193	1.55E-07	1.47E-06
ncbi_92270	ATP6AP1L	0.48	1.0775	1.166582	1.56E-07	1.48E-06
ncbi_7139	TNNT2	0.3475	0.001	-8.44087	1.58E-07	1.50E-06
ncbi_56649	TMPRSS4	0.43	0.1025	-2.06871	1.91E-07	1.78E-06
MSTRG.9452	UTY	1.74	4.185	1.26614	1.95E-07	1.82E-06
ncbi_283710	LOC283710	1.4725	3.2175	1.127673	2.15E-07	1.99E-06
MSTRG.7956	RABGEF1	4.59	2.0525	-1.16111	2.18E-07	2.01E-06
ncbi_2006	ELN	0.605	0.245	-1.30415	2.20E-07	2.02E-06
MSTRG.15515	--	1.175	3.64	1.631278	2.28E-07	2.10E-06
ncbi_81558	FAM117A	0.065	0.3825	2.556948	2.45E-07	2.24E-06
ncbi_6565	SLC15A2	0.0925	0.3625	1.970456	2.59E-07	2.35E-06
ncbi_3589	IL11	0.9175	0.29	-1.66166	2.73E-07	2.46E-06
ncbi_286204	CRB2	0.815	0.355	-1.19898	2.81E-07	2.53E-06
MSTRG.14798	C16orf89	3.8375	1.5775	-1.28253	2.98E-07	2.67E-06
ncbi_84814	PLPP7	1.0425	0.285	-1.87101	3.41E-07	3.02E-06
MSTRG.1223	ZNF283	1.5625	3.285	1.072037	3.45E-07	3.06E-06
MSTRG.7633	KIF27	1.805	0.84	-1.10354	3.51E-07	3.11E-06
MSTRG.5537	--	1.34	0.3525	-1.92654	3.54E-07	3.13E-06
ncbi_333926	PPM1J	0.7625	1.9875	1.382146	3.59E-07	3.18E-06
ncbi_151556	GPR155	0.31	0.6925	1.159546	3.72E-07	3.27E-06
ncbi_83882	TSPAN10	2.0625	1.025	-1.00877	3.90E-07	3.43E-06
ncbi_2786	GNG4	0.645	0.2875	-1.16574	3.99E-07	3.49E-06
ncbi_6094	ROM1	1.8525	0.8	-1.2114	4.05E-07	3.54E-06
ncbi_4207	BORCS8-MEF2B	2.085	4.3475	1.060139	4.24E-07	3.69E-06
ncbi_9214	FCMR	0.4125	0.08	-2.36632	4.39E-07	3.81E-06
ncbi_395	ARHGAP6	0.3	0.08	-1.90689	4.56E-07	3.95E-06
ncbi_7318	UBA7	0.185	0.6075	1.715359	4.67E-07	4.04E-06
ncbi_9095	TBX19	0.6775	1.435	1.082758	4.75E-07	4.10E-06
ncbi_8519	IFITM1	3.6925	1.595	-1.21104	4.90E-07	4.21E-06
MSTRG.2209	--	1.255	0.3175	-1.98286	5.21E-07	4.46E-06
MSTRG.13799	ZNF91	1.1125	2.605	1.227478	5.26E-07	4.49E-06
ncbi_81606	LBH	7.6025	3.4225	-1.15142	5.28E-07	4.50E-06
ncbi_440689	HIST2H2BF	3.8575	1.4225	-1.43924	5.61E-07	4.76E-06
ncbi_259308	FAM205A	0.0925	0.405	2.130397	5.98E-07	5.05E-06
ncbi_221527	ZBTB12	1.205	0.5425	-1.15134	6.07E-07	5.12E-06
MSTRG.2721	--	2.12	0.4025	-2.397	6.64E-07	5.56E-06
MSTRG.1990	--	0.6725	1.515	1.171712	6.67E-07	5.59E-06
ncbi_51702	PADI3	0.215	0.015	-3.8413	6.84E-07	5.71E-06
ncbi_3641	INSL4	2.7975	1.165	-1.26381	6.95E-07	5.79E-06
ncbi_9901	SRGAP3	0.24	0.54	1.169925	7.04E-07	5.86E-06
ncbi_56241	SUSD2	1.6525	0.685	-1.27047	7.39E-07	6.13E-06

ID	Symbol	CK mean	T mean	Log2(FC)	P-value	FDR
ncbi_112399	EGLN3	2.67	1.3	-1.03833	7.81E-07	6.45E-06
ncbi_1212	CLTB	109.9375	44.68	-1.29898	7.86E-07	6.48E-06
ncbi_117144	CATSPER1	0.435	0.115	-1.91938	7.96E-07	6.55E-06
ncbi_135	ADORA2A	0.6325	1.285	1.022631	8.22E-07	6.75E-06
MSTRG.10543	--	0.855	1.9625	1.198696	8.34E-07	6.84E-06
ncbi_23418	CRB1	0.5525	1.275	1.206451	8.57E-07	7.01E-06
ncbi_643866	CBLN3	1.11	0.43	-1.36815	8.85E-07	7.22E-06
ncbi_5268	SERPINB5	1.21	0.47	-1.36427	9.08E-07	7.39E-06
ncbi_131578	LRRC15	0.33	0.0825	-2	9.47E-07	7.68E-06
ncbi_2902	GRIN1	0.7425	0.37	-1.00487	9.63E-07	7.79E-06
ncbi_388323	GLTPD2	4.9925	2.47	-1.01525	1.04E-06	8.33E-06
ncbi_80852	GRIP2	0.3025	0.685	1.179169	1.04E-06	8.37E-06
ncbi_8862	APLN	0.6725	0.23	-1.5479	1.06E-06	8.48E-06
ncbi_3134	HLA-F	1.06	0.325	-1.70555	1.10E-06	8.78E-06
ncbi_5345	SERPINF2	0.9775	0.365	-1.4212	1.12E-06	8.93E-06
ncbi_128312	HIST3H2BB	7.23	2.32	-1.63987	1.13E-06	8.97E-06
MSTRG.9569	GVQW1	0.2975	0.9325	1.648214	1.15E-06	9.15E-06
MSTRG.14492	C16orf89	1.8775	4.3425	1.209713	1.21E-06	9.57E-06
ncbi_54866	PPP1R14D	1.1275	0.155	-2.86279	1.26E-06	9.90E-06
MSTRG.292	--	1.695	0.13	-3.7047	1.41E-06	1.10E-05
ncbi_29881	NPC1L1	0.0825	0.3875	2.23173	1.43E-06	1.11E-05
ncbi_374739	TEPP	0.2225	0.825	1.890589	1.48E-06	1.15E-05
ncbi_51703	ACSL5	0.515	0.18	-1.51658	1.50E-06	1.16E-05
ncbi_4320	MMP11	0.9975	0.42	-1.24793	1.85E-06	1.42E-05
ncbi_553158	PRR5-ARHGAP8	0.8925	1.8975	1.088176	1.94E-06	1.47E-05
MSTRG.14518	--	1.0475	2.585	1.303214	2.04E-06	1.55E-05
ncbi_128414	NKAIN4	1.025	0.435	-1.23654	2.14E-06	1.62E-05
ncbi_114769	CARD16	0.6175	0.0675	-3.19348	2.16E-06	1.63E-05
ncbi_1187	CLCNKA	1.405	0.6875	-1.03114	2.21E-06	1.66E-05
ncbi_1015	CDH17	0.55	0.2225	-1.30563	2.27E-06	1.70E-05
MSTRG.4642	Pol	0.9975	0.1975	-2.33646	2.39E-06	1.79E-05
ncbi_25780	RASGRP3	0.915	0.4525	-1.01585	2.58E-06	1.91E-05
ncbi_100271849	MEF2B	1.7875	3.925	1.134749	2.62E-06	1.94E-05
ncbi_643382	TMEM253	1.4875	0.6925	-1.103	2.75E-06	2.03E-05
ncbi_1768	DNAH6	0.07	0.1825	1.38247	2.79E-06	2.05E-05
ncbi_27063	ANKRD1	0.2875	0.02	-3.84549	2.89E-06	2.12E-05
MSTRG.16364	C16orf89	0.5175	1.2975	1.326104	3.29E-06	2.40E-05
ncbi_51351	ZNF117	0.535	0.235	-1.18688	3.47E-06	2.52E-05
ncbi_51764	GNG13	1.57	0.6275	-1.32308	3.48E-06	2.52E-05
ncbi_79816	TLE6	0.9075	1.9225	1.083014	3.65E-06	2.63E-05
ncbi_9379	NRXN2	0.1725	0.0425	-2.02106	3.73E-06	2.68E-05
ncbi_55124	PIWIL2	0.695	0.2525	-1.46073	3.85E-06	2.76E-05
MSTRG.2703	--	1.0075	3.46	1.779992	3.92E-06	2.80E-05
ncbi_116535	MRGPRF	0.75	0.2475	-1.59946	3.98E-06	2.84E-05
MSTRG.17427	NPIPA8	1.4125	3.7825	1.421089	4.04E-06	2.88E-05
MSTRG.4525	GVQW1	1.0425	0.2825	-1.88372	4.21E-06	2.99E-05
ncbi_8626	TP63	0.4475	0.16	-1.48382	4.22E-06	2.99E-05
MSTRG.6225	--	1.97	5.3025	1.428477	4.40E-06	3.10E-05
ncbi_2635	GBP3	0.4725	0.1175	-2.00765	4.44E-06	3.13E-05
MSTRG.524	--	1.315	3.0125	1.195898	4.47E-06	3.15E-05
ncbi_3965	LGALS9	1.7225	0.835	-1.04466	4.49E-06	3.16E-05
ncbi_4148	MATN3	0.69	0.215	-1.68226	4.55E-06	3.20E-05
ncbi_6925	TCF4	0.115	0.0225	-2.35364	4.59E-06	3.22E-05
MSTRG.6073	UTY	0.8275	2.0375	1.299969	4.62E-06	3.24E-05

ID	Symbol	CK mean	T mean	Log2(FC)	P-value	FDR
MSTRG.9251	--	0.45	1.58	1.811928	4.87E-06	3.40E-05
ncbi_24	ABCA4	0.3	0.0925	-1.69744	4.95E-06	3.45E-05
ncbi_1101	CHAD	0.555	1.1975	1.109466	4.99E-06	3.47E-05
ncbi_59350	RXFP1	0.4325	0.1625	-1.41226	5.16E-06	3.59E-05
MSTRG.13502	--	0.46	1.285	1.482063	5.30E-06	3.68E-05
ncbi_647024	C6orf132	0.1975	0.06	-1.71882	5.34E-06	3.70E-05
ncbi_149478	BTBD19	1.2475	0.6075	-1.03808	5.35E-06	3.70E-05
ncbi_5973	RENBP	0.79	0.2325	-1.76462	5.46E-06	3.77E-05
ncbi_284194	LGALS9B	0.355	0.9725	1.453879	5.59E-06	3.86E-05
MSTRG.12248	--	1.2325	0.37	-1.73599	5.69E-06	3.91E-05
ncbi_26291	FGF21	0.58	0.115	-2.33442	5.87E-06	4.03E-05
ncbi_8436	CAVIN2	0.4025	0.0975	-2.04551	5.95E-06	4.08E-05
ncbi_284434	NWD1	0.125	0.02	-2.64386	6.83E-06	4.63E-05
ncbi_339761	CYP27C1	0.4225	0.2025	-1.06103	6.92E-06	4.68E-05
ncbi_57834	CYP4F11	0.04	0.255	2.672425	7.01E-06	4.73E-05
ncbi_23452	ANGPTL2	0.3225	0.105	-1.61891	7.11E-06	4.79E-05
MSTRG.564	OR1F12	1.94	4.47	1.204218	7.14E-06	4.81E-05
ncbi_60529	ALX4	0.205	0.45	1.134301	8.14E-06	5.42E-05
ncbi_3008	HIST1H1E	1.99	0.4525	-2.13678	8.34E-06	5.54E-05
ncbi_353322	ANKRD37	6.355	3.01	-1.07813	9.45E-06	6.20E-05
ncbi_9568	GABBR2	0.0525	0.001	-5.71425	1.02E-05	6.64E-05
MSTRG.220	GVQW1	0.4675	1.22	1.383843	1.03E-05	6.68E-05
ncbi_27071	DAPP1	0.5075	0.17	-1.57787	1.06E-05	6.86E-05
ncbi_3604	TNFRSF9	0.4325	0.1925	-1.16784	1.06E-05	6.89E-05
ncbi_93082	NEURL3	0.1725	0.001	-7.43045	1.10E-05	7.05E-05
ncbi_653604	HIST2H3D	3.5025	1.32	-1.40785	1.12E-05	7.17E-05
ncbi_9056	SLC7A7	0.95	0.3975	-1.25697	1.15E-05	7.34E-05
MSTRG.10841	--	0.86	1.97	1.195787	1.17E-05	7.51E-05
ncbi_79690	GAL3ST4	0.1025	0.3975	1.955331	1.18E-05	7.56E-05
MSTRG.4544	--	1.0925	0.2675	-2.03002	1.20E-05	7.65E-05
ncbi_9724	UTP14C	0.17	1.8075	3.410389	1.30E-05	8.28E-05
MSTRG.3541	--	0.59	1.735	1.556149	1.46E-05	9.13E-05
ncbi_8542	APOL1	0.715	0.35	-1.03059	1.49E-05	9.29E-05
ncbi_554	AVPR2	0.245	0.005	-5.61471	1.57E-05	9.78E-05
ncbi_105371921	LOC105371921	2.3775	1.0975	-1.11522	1.58E-05	9.81E-05
ncbi_339541	ARMH1	0.5175	1.205	1.219402	1.59E-05	9.86E-05
ncbi_169270	ZNF596	0.3125	0.07	-2.15843	1.62E-05	0.0001
ncbi_5105	PCK1	1.33	0.66	-1.01089	1.70E-05	0.000105
ncbi_150372	NFAM1	0.125	0.02	-2.64386	1.72E-05	0.000106
ncbi_5346	PLIN1	0.0625	0.26	2.056584	1.72E-05	0.000106
ncbi_107985729	LOC107985729	0.9775	0.2875	-1.76553	1.79E-05	0.00011
MSTRG.9339	ITCH	0.8525	2.4775	1.539113	1.80E-05	0.000111
ncbi_646851	FAM227A	1.555	3.115	1.002318	2.04E-05	0.000124
ncbi_266722	HS6ST3	0.215	0.495	1.203092	2.04E-05	0.000124
ncbi_2318	FLNC	0.0775	0.0125	-2.63227	2.26E-05	0.000136
MSTRG.208	Rpl12	2.655	1.155	-1.20082	2.28E-05	0.000137
ncbi_100130827	SBK3	1.87	0.895	-1.06308	2.30E-05	0.000138
ncbi_56648	EIF5A2	0.415	0.85	1.034352	2.31E-05	0.000138
ncbi_27111	SDCBP2	0.8025	1.7625	1.13505	2.32E-05	0.000138
ncbi_3316	HSPB2	0.275	0.001	-8.10329	2.34E-05	0.00014
ncbi_199223	TTC21A	0.435	0.895	1.040872	2.34E-05	0.00014
ncbi_2979	GUCA1B	0.15	0.4775	1.670538	2.35E-05	0.00014
ncbi_100507003	GFY	0.275	0.01	-4.78136	2.36E-05	0.00014
ncbi_1141	CHRNB2	0.155	0.03	-2.36923	2.40E-05	0.000143

ID	Symbol	CK mean	T mean	Log2(FC)	P-value	FDR
MSTRG.13469	--	2.9475	1.41	-1.0638	2.41E-05	0.000144
ncbi_220004	PPP1R32	0.835	1.7525	1.069566	2.45E-05	0.000146
ncbi_55084	SOBP	0.3875	0.1775	-1.12638	2.51E-05	0.000149
ncbi_7700	ZNF141	0.42	0.86	1.033947	2.52E-05	0.000149
ncbi_196500	PIANP	0.3675	0.845	1.201207	2.74E-05	0.000161
ncbi_100129924	TMEM269	0.3025	0.6175	1.029504	2.83E-05	0.000166
ncbi_124976	SPNS2	1.0425	0.51	-1.03148	3.02E-05	0.000176
ncbi_999	CDH1	0.455	0.1375	-1.72643	3.14E-05	0.000183
MSTRG.17592	--	0.73	2.0425	1.484368	3.53E-05	0.000203
ncbi_286	ANK1	0.365	0.17	-1.10236	3.82E-05	0.000219
ncbi_2570	GABRR2	0.285	0.095	-1.58496	3.84E-05	0.000219
MSTRG.8073	OR1F12	1.2475	0.435	-1.51995	3.89E-05	0.000222
ncbi_79413	ZBED2	0.11	0.001	-6.78136	3.90E-05	0.000222
ncbi_2494	NR5A2	0.2075	0.0575	-1.85148	3.92E-05	0.000223
ncbi_2323	FLT3LG	1.72	0.7975	-1.10885	4.00E-05	0.000227
ncbi_84699	CREB3L3	0.535	0.195	-1.45606	4.18E-05	0.000237
ncbi_729857	RGPD2	0.055	0.001	-5.78136	4.21E-05	0.000239
ncbi_348013	TMEM255B	0.825	0.3225	-1.35509	4.27E-05	0.000241
MSTRG.8273	--	0.585	1.585	1.437974	4.42E-05	0.000249
ncbi_1109	AKR1C4	0.5975	0.0675	-3.14598	4.52E-05	0.000253
ncbi_3575	IL7R	0.2825	0.1025	-1.46263	4.71E-05	0.000263
MSTRG.1611	pro-pol	2.4125	1.1125	-1.11672	4.86E-05	0.00027
ncbi_79846	CFAP69	0.435	0.8775	1.012384	4.89E-05	0.000271
ncbi_2563	GABRD	0.62	0.295	-1.07155	4.92E-05	0.000273
ncbi_90993	CREB3L1	0.265	0.0425	-2.64046	4.93E-05	0.000273
ncbi_141	ADPRH	0.3975	0.125	-1.66903	5.05E-05	0.00028
ncbi_645121	CCNI2	0.735	0.3125	-1.23389	5.29E-05	0.000291
ncbi_1520	CTSS	0.35	0.09	-1.95936	5.44E-05	0.000299
ncbi_9033	PKD2L1	0.375	0.1475	-1.34618	6.21E-05	0.000337
MSTRG.10950	--	1.62	3.84	1.245112	6.39E-05	0.000346
MSTRG.5976	PPP5D1	0.85	2.4675	1.537515	6.46E-05	0.00035
ncbi_130733	TMEM178A	0.345	0.8625	1.321928	6.61E-05	0.000357
ncbi_138715	ARID3C	0.495	1	1.0145	6.62E-05	0.000357
ncbi_3576	CXCL8	0.34	0.015	-4.5025	6.65E-05	0.000358
ncbi_340351	AGBL3	0.35	0.8775	1.326044	6.89E-05	0.00037
ncbi_27122	DKK3	0.3625	0.1025	-1.82236	6.96E-05	0.000373
ncbi_733	C8G	1.305	0.48	-1.44294	7.39E-05	0.000394
ncbi_113451	AZIN2	0.38	0.8675	1.190864	7.67E-05	0.000407
ncbi_84632	AFAP1L2	0.3775	0.0675	-2.48352	7.74E-05	0.00041
ncbi_2901	GRIK5	0.545	0.245	-1.15347	8.04E-05	0.000423
ncbi_728310	GOLGA6L7	0.2375	0.0225	-3.39993	8.26E-05	0.000434
ncbi_5745	PTH1R	0.39	0.1375	-1.50404	8.96E-05	0.000467
ncbi_1734	DIO2	0.6475	0.2975	-1.12199	9.40E-05	0.000487
ncbi_8784	TNFRSF18	0.86	0.275	-1.64491	9.59E-05	0.000496
ncbi_8681	JMJD7-PLA2G4B	2.345	0.005	-8.87344	9.61E-05	0.000497
MSTRG.17316	UTY	0.265	1	1.915936	9.81E-05	0.000506
ncbi_140893	RBBP8NL	0.155	0.0175	-3.14684	9.82E-05	0.000506
ncbi_286223	C9orf47	0.2575	0.5875	1.190016	9.89E-05	0.00051
ncbi_316	AOX1	0.215	0.055	-1.96683	0.000103	0.000527
MSTRG.4387	GUSB	0.42	1.3425	1.676461	0.000104	0.000531
ncbi_3479	IGF1	0.0675	0.01	-2.75489	0.000107	0.000548
ncbi_84467	FBN3	0.1825	0.0675	-1.43494	0.000109	0.000555
ncbi_10991	SLC38A3	0.565	0.2275	-1.31238	0.000109	0.000558
ncbi_4606	MYBPC2	0.225	0.0525	-2.09954	0.000116	0.000586

ID	Symbol	CK mean	T mean	Log2(FC)	P-value	FDR
MSTRG.17601	POLR3E	0.0975	2.545	4.70612	0.000121	0.000612
ncbi_4323	MMP14	0.05	0.2425	2.277985	0.000123	0.000622
ncbi_5896	RAG1	0.0875	0.2075	1.245756	0.000129	0.000646
MSTRG.17261	VARS	6.6125	14.59	1.141712	0.000144	0.000713
MSTRG.5941	ZNF91	2.7925	1.37	-1.02738	0.000145	0.00072
ncbi_25850	ZNF345	0.2625	0.6225	1.245756	0.000147	0.000728
MSTRG.1093	--	0.3275	1.115	1.767477	0.000148	0.000729
MSTRG.7176	--	3.5375	7.1775	1.020751	0.000149	0.000736
ncbi_8710	SERPINB7	0.105	0.001	-6.71425	0.000154	0.00076
MSTRG.17248	HLA-B	18.8325	3.4325	-2.45589	0.000159	0.000781
ncbi_147138	TMC8	1.015	0.4575	-1.14964	0.000167	0.000814
ncbi_4599	MX1	0.61	0.2675	-1.18927	0.000173	0.000838
ncbi_4583	MUC2	0.0225	0.001	-4.49185	0.000175	0.000848
MSTRG.5536	--	0.425	1.185	1.479352	0.000177	0.000859
MSTRG.10378	RPGRIP1L	1.0725	2.8425	1.406183	0.000189	0.000909
ncbi_8332	HIST1H2AL	1.3325	0.3375	-1.98118	0.000192	0.000922
ncbi_10107	TRIM10	0.5675	0.28	-1.01919	0.000202	0.000967
ncbi_257062	CATSPERD	0.19	0.0225	-3.078	0.00021	0.001003
MSTRG.15736	RPL12	4.4375	9.5575	1.106886	0.000212	0.001009
ncbi_6752	SSTR2	0.0725	0.1725	1.250543	0.000217	0.00103
MSTRG.4088	ZNF91	1.0625	2.635	1.31034	0.000218	0.001032
ncbi_94	ACVRL1	0.5575	0.23	-1.27734	0.000233	0.001099
ncbi_3860	KRT13	0.13	0.001	-7.02237	0.000236	0.00111
MSTRG.17119	RPS18	103.3725	266.8225	1.368028	0.000239	0.001123
ncbi_2520	GAST	1.2	0.2175	-2.46395	0.000245	0.001146
ncbi_51554	ACKR4	0.1525	0.425	1.478654	0.000246	0.001149
ncbi_6512	SLC1A7	0.2325	0.0475	-2.29123	0.000251	0.00117
ncbi_84439	HHIPL1	0.1275	0.3125	1.293359	0.000261	0.001212
ncbi_3560	IL2RB	0.625	0.29	-1.1078	0.000261	0.001215
MSTRG.17112	EHMT2	0.001	4.4325	12.1139	0.000263	0.001221
ncbi_140564	APOBEC3D	0.001	0.0875	6.451211	0.000265	0.001228
ncbi_4776	NFATC4	0.275	0.13	-1.08092	0.000267	0.001236
ncbi_857	CAV1	24.9975	12.2675	-1.02694	0.000279	0.001288
MSTRG.10347	--	2.535	1.0725	-1.24101	0.000287	0.001318
ncbi_56606	SLC2A9	0.6625	0.28	-1.24249	0.000291	0.001335
ncbi_55107	ANO1	0.04	0.001	-5.32193	0.000301	0.001374
ncbi_4688	NCF2	0.5	0.1725	-1.53533	0.000306	0.001393
ncbi_63924	CIDEC	0.315	0.03	-3.39232	0.000311	0.001413
MSTRG.2887	--	0.66	1.615	1.290996	0.000312	0.001417
ncbi_94115	CGB8	0.4075	0.03	-3.76377	0.000316	0.001437
ncbi_5143	PDE4C	0.9375	0.4625	-1.01937	0.000322	0.00146
ncbi_53905	DUOX1	0.195	0.395	1.018379	0.000328	0.001483
ncbi_8968	HIST1H3F	1.7525	0.63	-1.47599	0.000331	0.001494
ncbi_390937	LOC390937	0.0975	0.33	1.758992	0.000333	0.001505
ncbi_79442	LRRC2	0.31	0.1125	-1.46234	0.000334	0.001508
ncbi_3122	HLA-DRA	0.37	0.0475	-2.96153	0.000337	0.001519
ncbi_10216	PRG4	0.185	0.405	1.130397	0.000345	0.001549
MSTRG.17552	LENG9	0.001	1.9075	10.89747	0.000347	0.001559
ncbi_266743	NPAS4	0.001	0.06	5.906891	0.000365	0.00163
ncbi_5579	PRKCB	0.1425	0.035	-2.02554	0.000383	0.0017
ncbi_153478	PLEKHG4B	0.0525	0.0075	-2.80735	0.000396	0.001749
ncbi_196743	PAOX	0.3475	0.0575	-2.59538	0.000399	0.00176
ncbi_100131378	C11orf91	0.4375	0.1175	-1.89662	0.000403	0.001778
ncbi_11148	HHLA2	0.065	0.001	-6.02237	0.000404	0.001781

ID	Symbol	CK mean	T mean	Log2(FC)	P-value	FDR
ncbi_122402	TDRD9	0.115	0.015	-2.9386	0.000407	0.00179
ncbi_768239	PSAPL1	0.08	0.01	-3	0.000407	0.001792
ncbi_7060	THBS4	0.1575	0.3975	1.335603	0.00041	0.001802
ncbi_84071	ARMC2	0.355	0.725	1.030162	0.000422	0.001851
ncbi_9037	SEMA5A	0.1425	0.0575	-1.30933	0.000423	0.001853
ncbi_80008	TMEM156	1.025	0.505	-1.02127	0.000428	0.001871
ncbi_152189	CMTM8	0.72	1.83	1.345775	0.000433	0.001891
MSTRG.10927	--	0.3075	1.7075	2.473227	0.000433	0.001891
ncbi_56099	PCDHGB7	0.2875	0.09	-1.67557	0.000437	0.001907
ncbi_26287	ANKRD2	0.8175	0.3375	-1.27633	0.000451	0.001957
ncbi_55106	SLFN12	0.1025	0.015	-2.77259	0.000455	0.001969
ncbi_10870	HCST	0.63	0.0775	-3.02308	0.000463	0.002003
ncbi_54437	SEMA5B	0.335	0.1325	-1.33817	0.000467	0.002016
ncbi_3702	ITK	0.145	0.04	-1.85798	0.000471	0.002031
ncbi_157855	KCNU1	0.5025	0.175	-1.52177	0.000472	0.002032
ncbi_149461	CLDN19	0.12	0.0125	-3.26303	0.000494	0.002116
ncbi_4620	MYH2	0.0725	0.01	-2.85798	0.000504	0.002153
ncbi_100996379	LOC100996379	0.0525	0.3875	2.883807	0.000505	0.002159
ncbi_2155	F7	0.3	0.1125	-1.41504	0.000506	0.002162
ncbi_9938	ARHGAP25	0.1375	0.0175	-2.974	0.000517	0.002203
ncbi_100130933	SMIM6	0.805	0.2775	-1.5365	0.000521	0.002219
ncbi_6469	SHH	0.48	0.1925	-1.31818	0.000534	0.002267
MSTRG.11755	UTY	0.4725	1.0025	1.085216	0.000544	0.002303
MSTRG.7649	--	3.37	6.7525	1.002673	0.000548	0.00232
MSTRG.17050	PPP2R3B	0.2	1.2625	2.658211	0.000563	0.002374
ncbi_166336	PRICKLE2	0.0675	0.02	-1.75489	0.000597	0.002502
ncbi_84517	ACTRT3	1.13	0.545	-1.05199	0.000607	0.002539
MSTRG.1858	PPP5D1	2.0125	0.92	-1.12928	0.00066	0.002733
MSTRG.12080	--	0.8875	1.9125	1.107641	0.000678	0.002798
MSTRG.17415	MARF1	0.001	2.965	11.53382	0.000679	0.002803
ncbi_100141515	C17orf99	0.1475	0.001	-7.20457	0.000696	0.002863
ncbi_107985678	LOC107985678	0.445	0.9275	1.059542	0.000709	0.002912
MSTRG.15320	TIGD1	1.57	0.7575	-1.05145	0.000711	0.002916
ncbi_400629	TEX19	0.455	1.025	1.171685	0.000749	0.003058
ncbi_84519	ACRBP	0.3675	0.905	1.300174	0.000776	0.003159
ncbi_1638	DCT	0.05	0.17	1.765535	0.000787	0.0032
ncbi_57718	PPP4R4	0.001	0.055	5.78136	0.000792	0.003218
ncbi_387763	C11orf96	1.4	0.6575	-1.09036	0.000801	0.003249
ncbi_100289279	CNTNAP3C	0.435	0.8775	1.012384	0.000804	0.003262
ncbi_51032	CELA2B	0.32	0.85	1.409391	0.000809	0.003278
ncbi_84941	HSH2D	0.645	0.1875	-1.78241	0.000831	0.003362
ncbi_1470	CST2	1.5825	0.6775	-1.22391	0.00089	0.003565
ncbi_55186	SLC25A36	8.4125	21.3275	1.342108	0.000921	0.003677
ncbi_6263	RYR3	0.0325	0.01	-1.70044	0.000927	0.003694
ncbi_254228	CALHM5	0.015	0.001	-3.90689	0.000929	0.003703
ncbi_5577	PRKAR2B	0.16	0.36	1.169925	0.000953	0.003788
ncbi_321	APBA2	0.3975	0.195	-1.02748	0.000968	0.003841
ncbi_85480	TSLP	0.115	0.3875	1.752562	0.000971	0.003852
ncbi_100131539	ZNF705E	0.0925	0.255	1.462972	0.000976	0.003868
ncbi_389860	PAGE2B	0.215	0.9175	2.093371	0.000992	0.003926
ncbi_55231	CCDC87	0.285	0.08	-1.83289	0.000996	0.003939
ncbi_58529	MYOZ1	0.11	0.001	-6.78136	0.000997	0.003941
ncbi_283726	SAXO2	0.1975	0.5	1.340075	0.000998	0.003944
ncbi_56171	DNAH7	0.0875	0.19	1.118644	0.001001	0.003954

ID	Symbol	CK mean	T mean	Log2(FC)	P-value	FDR
MSTRG.6576	RNF181	0.7475	2.2425	1.584963	0.001008	0.003977
ncbi_27156	RSPH14	0.0675	0.3775	2.483517	0.001022	0.004026
ncbi_1261	CNGA3	0.1675	0.025	-2.74416	0.001033	0.004064
ncbi_29767	TMOD2	0.0775	0.17	1.133267	0.001042	0.004092
ncbi_53347	UBASH3A	0.0575	0.001	-5.84549	0.001048	0.004112
ncbi_445577	C9orf129	0.48	0.22	-1.12553	0.001051	0.004123
ncbi_494513	PJVK	0.7475	0.325	-1.20163	0.001065	0.004171
MSTRG.1468	RPS10	0.1725	0.4725	1.453718	0.001065	0.004171
ncbi_338382	RAB7B	0.545	0.2625	-1.05394	0.001068	0.004181
ncbi_164395	TTLL9	0.21	0.0725	-1.53434	0.001071	0.004189
ncbi_56103	PCDHGB2	0.23	0.0725	-1.66558	0.001102	0.004299
ncbi_23415	KCNH4	0.2675	0.61	1.18927	0.001112	0.004329
ncbi_105374013	LOC105374013	0.685	1.3825	1.013104	0.001115	0.004336
ncbi_212	ALAS2	0.035	0.1925	2.459432	0.001116	0.00434
ncbi_79057	PRRG3	0.2975	0.035	-3.08746	0.001124	0.004365
ncbi_25823	TPSG1	0.205	0.0275	-2.89812	0.001194	0.004589
MSTRG.17067	VAMP7	0.5	6.6875	3.741467	0.001222	0.004682
MSTRG.10918	--	0.5775	1.7175	1.572417	0.001224	0.004688
MSTRG.17389	GOLGA6L10	0.001	0.6125	9.258566	0.001275	0.004857
ncbi_7479	WNT8B	0.0075	0.12	4	0.001307	0.004963
ncbi_9283	GPR37L1	0.16	0.065	-1.29956	0.001308	0.004964
ncbi_197	AHSG	0.115	0.435	1.919382	0.001376	0.005201
ncbi_56605	ERO1B	0.0075	0.08	3.415037	0.001387	0.005238
ncbi_171483	FAM9B	0.0975	0.001	-6.60733	0.001406	0.005301
ncbi_144321	GLIPR1L2	0.125	0.3525	1.495695	0.00141	0.005313
ncbi_6866	TAC3	0.565	0.095	-2.57225	0.00148	0.005531
MSTRG.7036	C16orf89	1.2275	2.475	1.011706	0.001499	0.005588
ncbi_158248	TTC16	0.42	0.1975	-1.08854	0.001536	0.005707
MSTRG.14041	--	1.045	2.22	1.087057	0.001549	0.005751
ncbi_440829	SHISA8	0.02	0.1675	3.066089	0.001551	0.005756
ncbi_220979	C10orf25	0.375	0.16	-1.22882	0.001559	0.00578
ncbi_4135	MAP6	0.115	0.01	-3.52356	0.00158	0.005843
MSTRG.9717	--	1.21	0.3675	-1.71919	0.001617	0.005966
ncbi_84249	PSD2	0.0725	0.1825	1.331844	0.001627	0.006003
ncbi_158584	FAAH2	0.655	0.3225	-1.0222	0.001689	0.006208
ncbi_118490	MSS51	0.2175	0.575	1.402547	0.001731	0.006342
ncbi_7062	TCHH	0.07	0.02	-1.80735	0.001792	0.006536
ncbi_347454	SOWAHD	0.6575	0.29	-1.18094	0.001793	0.006538
ncbi_349565	NMNAT3	0.14	0.355	1.342392	0.001794	0.006539
ncbi_140731	ANKRD60	0.065	0.0025	-4.70044	0.001797	0.006548
ncbi_6004	RGS16	0.1025	0.435	2.085391	0.001809	0.006586
MSTRG.17550	LENG8	0.1175	2.425	4.367252	0.001821	0.006622
MSTRG.14915	--	0.66	1.6025	1.279786	0.001828	0.006643
MSTRG.12543	--	0.3125	0.975	1.641546	0.001834	0.006658
MSTRG.9700	--	0.42	1.3275	1.660251	0.001846	0.006701
MSTRG.17610	METTL9	13.1475	39.375	1.582491	0.001855	0.006729
ncbi_10004	NAALADL1	0.0375	0.165	2.137504	0.001861	0.006747
ncbi_643669	CCER2	0.1175	0.3825	1.702799	0.001872	0.006782
MSTRG.17444	MRM1	0.001	1.1925	10.21977	0.001873	0.006788
ncbi_126549	ANKLE1	0.1825	0.45	1.302029	0.00193	0.006974
ncbi_5017	OVOL1	0.05	0.1825	1.867896	0.001935	0.006986
MSTRG.14021	GVQW1	1.535	0.4225	-1.86122	0.001954	0.007042
ncbi_55224	ETNK2	0.625	0.3025	-1.04692	0.001998	0.007176
ncbi_55150	C19orf73	0.395	1.21	1.615082	0.002004	0.007193

ID	Symbol	CK mean	T mean	Log2(FC)	P-value	FDR
ncbi_2914	GRM4	0.0725	0.015	-2.27302	0.002018	0.007233
ncbi_107984859	LOC107984859	0.01	0.1425	3.83289	0.002027	0.007259
ncbi_100130370	LOC100130370	3.99	9.0775	1.185906	0.002088	0.007451
ncbi_164592	CCDC116	0.0775	0.2525	1.704015	0.002126	0.007581
MSTRG.12409	LCMT1	1.09	2.21	1.019718	0.002135	0.007607
MSTRG.10325	Rpl7	0.1825	0.89	2.285909	0.002156	0.007673
MSTRG.6835	--	0.87	1.885	1.115477	0.002165	0.007697
ncbi_100130274	CCDC166	0.205	0.6	1.549339	0.002178	0.007731
ncbi_1589	CYP21A2	0.0625	0.001	-5.96578	0.002182	0.007743
ncbi_84700	MYO18B	0.0125	0.055	2.137504	0.002211	0.007831
ncbi_100506115	SMIM25	0.4975	0.215	-1.21036	0.002223	0.007864
MSTRG.12025	--	0.92	2.065	1.166436	0.00227	0.008014
ncbi_51151	SLC45A2	0.1225	0.005	-4.61471	0.002365	0.008296
ncbi_9032	TM4SF5	0.7675	0.215	-1.83583	0.002366	0.008296
ncbi_9745	ZNF536	0.001	0.025	4.643856	0.002377	0.00833
ncbi_1463	NCAN	0.0575	0.0125	-2.20163	0.002391	0.008371
ncbi_56936	CCDC177	0.09	0.0175	-2.36257	0.002394	0.008378
ncbi_23107	MRPS27	19.0675	41.0175	1.105124	0.0024	0.008397
ncbi_1780	DYNC1I1	0.8325	0.395	-1.0756	0.002426	0.008476
MSTRG.6836	UTY	1.2175	2.885	1.24465	0.002463	0.008586
ncbi_255809	C19orf38	0.1725	0.5125	1.570956	0.002463	0.008586
MSTRG.1065	--	10.5875	2.575	-2.03972	0.002477	0.008627
ncbi_84651	SPINK7	1.515	0.6225	-1.28317	0.002531	0.008782
MSTRG.1925	--	1.32	0.605	-1.12553	0.002605	0.008996
MSTRG.16775	--	0.5775	1.3625	1.238363	0.002657	0.009159
ncbi_9717	SEC14L5	0.05	0.14	1.485427	0.002669	0.009188
ncbi_8353	HIST1H3E	1.3975	0.43	-1.70044	0.002717	0.009328
ncbi_105373347	LOC105373347	0.5975	0.2475	-1.27151	0.002766	0.009472
ncbi_2825	GPR1	0.83	0.3625	-1.19513	0.002775	0.009492
ncbi_54921	CHTF8	19.31	7.3125	-1.40091	0.002808	0.009595
ncbi_100316904	SAP25	0.4975	1.01	1.021587	0.002812	0.009607
ncbi_4987	OPRL1	0.33	0.695	1.074547	0.002825	0.009646
ncbi_50632	CALY	0.465	0.215	-1.11289	0.00286	0.009754
ncbi_126147	NTN5	0.1775	0.4375	1.301464	0.002895	0.009855
MSTRG.9170	GVQW2	1.46	0.6525	-1.16192	0.002916	0.009914
ncbi_57161	PELI2	0.0675	0.1675	1.311202	0.002976	0.010091
MSTRG.17225	ATF6B	2.18	6.66	1.611194	0.003022	0.010221
MSTRG.11432	OR1F12	0.035	0.3875	3.468769	0.003093	0.010424
ncbi_140711	TLDC2	0.655	0.3	-1.12653	0.003116	0.01049
ncbi_1950	EGF	0.02	0.11	2.459432	0.003161	0.010621
MSTRG.8959	--	0.625	1.7025	1.445727	0.003268	0.010937
ncbi_624	BDKRB2	0.2725	0.0925	-1.55873	0.003298	0.01103
ncbi_5673	PSG5	0.38	0.07	-2.44057	0.003328	0.011106
ncbi_26049	FAM169A	0.1775	0.07	-1.34239	0.003335	0.011128
MSTRG.10881	GVQW1	0.5425	1.28	1.238449	0.003369	0.011233
ncbi_2984	GUCY2C	0.3125	0.1475	-1.08314	0.003417	0.011363
ncbi_26298	EHF	0.1175	0.03	-1.96963	0.003428	0.011391
ncbi_285268	ZNF621	2.7025	5.56	1.04079	0.003465	0.011498
ncbi_9058	SLC13A2	0.2175	0.075	-1.53605	0.003483	0.011553
MSTRG.4658	--	0.4875	1.1075	1.183833	0.003502	0.011599
ncbi_117157	SH2D1B	0.165	0.045	-1.87447	0.003516	0.011635
ncbi_4057	LTF	0.2925	0.1075	-1.4441	0.003519	0.011642
ncbi_51286	CEND1	0.8275	0.38	-1.12276	0.00358	0.011817
MSTRG.13479	--	0.2375	1.105	2.218047	0.003612	0.011901

ID	Symbol	CK mean	T mean	Log2(FC)	P-value	FDR
ncbi_79788	ZNF665	0.0425	0.001	-5.40939	0.003635	0.011973
ncbi_7805	LAPTM5	0.13	0.0175	-2.89308	0.003773	0.012393
ncbi_29802	VPREB3	0.24	1.03	2.101538	0.003797	0.01246
ncbi_221468	TMEM217	0.3375	0.0875	-1.94753	0.00385	0.01262
ncbi_147409	DSG4	0.025	0.001	-4.64386	0.003917	0.012814
ncbi_1735	DIO3	0.39	0.1725	-1.17688	0.004016	0.013096
MSTRG.14975	UTY	0.675	1.57	1.217805	0.004072	0.013252
ncbi_79983	POF1B	0.175	0.05	-1.80735	0.004145	0.013467
ncbi_163782	KANK4	0.165	0.07	-1.23704	0.004166	0.013515
ncbi_119467	CLRN3	0.7125	0.115	-2.63126	0.004173	0.013534
ncbi_149483	CCDC17	0.165	0.3675	1.155278	0.004187	0.013574
ncbi_434	ASIP	0.13	0.415	1.6746	0.004305	0.013909
ncbi_91703	ACY3	0.405	0.11	-1.88042	0.004345	0.01402
ncbi_388394	RPRML	0.215	0.58	1.431716	0.004431	0.014268
ncbi_728378	POTEF	0.2275	0.4925	1.114257	0.004528	0.014551
MSTRG.4252	GVQW1	0.545	1.2525	1.200482	0.004554	0.014617
ncbi_128434	VSTM2L	0.0075	0.1075	3.841302	0.00457	0.01466
ncbi_10219	KLRG1	0.43	0.935	1.12063	0.004582	0.014692
ncbi_55512	SMPD3	0.995	0.1775	-2.48688	0.004588	0.014709
ncbi_4143	MAT1A	0.005	0.0575	3.523562	0.004625	0.014808
ncbi_397	ARHGDIB	0.3725	0.13	-1.51873	0.004662	0.014919
ncbi_2981	GUCA2B	0.4	0.03	-3.73697	0.004668	0.014936
ncbi_27040	LAT	0.3425	0.735	1.10164	0.004733	0.015109
ncbi_285349	ZNF660	0.0025	0.0475	4.247928	0.004756	0.015163
ncbi_445582	POTEE	0.13	0.3125	1.265345	0.004786	0.015246
ncbi_54039	PCBP3	0.0275	0.17	2.628031	0.004809	0.015312
ncbi_7054	TH	0.18	0.045	-2	0.00493	0.015644
ncbi_10841	FTCD	0.2125	0.4525	1.090455	0.004985	0.015798
ncbi_241	ALOX5AP	0.4875	0.055	-3.1479	0.005045	0.015967
ncbi_105369535	LOC105369535	0.02	0.001	-4.32193	0.005077	0.016059
ncbi_5142	PDE4B	0.0625	0.0075	-3.05889	0.005119	0.016175
MSTRG.6072	--	0.4425	1.1725	1.405839	0.00512	0.016175
ncbi_3861	KRT14	0.26	0.075	-1.79355	0.005153	0.016265
ncbi_388325	SCIMP	0.1225	0.295	1.267933	0.005294	0.016647
ncbi_8843	HCAR3	0.63	0.2975	-1.08246	0.005305	0.016679
ncbi_112268347	LOC112268347	0.1175	0.3475	1.564352	0.005325	0.016722
ncbi_154064	RAET1L	0.7025	0.3075	-1.19191	0.005389	0.01689
ncbi_9331	B4GALT6	0.06	0.145	1.273018	0.005745	0.017848
ncbi_7047	TGM4	0.095	0.02	-2.24793	0.005797	0.017987
ncbi_25797	QPCT	0.001	0.0975	6.60733	0.005965	0.018428
ncbi_3009	HIST1H1B	0.4775	0.0875	-2.44815	0.00602	0.018582
ncbi_124590	USH1G	0.28	0.1175	-1.25277	0.006051	0.018655
ncbi_79895	ATP8B4	0.1975	0.0875	-1.1745	0.006075	0.018723
ncbi_345	APOC3	0.71	0.125	-2.50589	0.006147	0.01892
ncbi_58484	NLRC4	0.3325	0.165	-1.01089	0.006164	0.018958
MSTRG.11024	--	0.3775	1.135	1.588144	0.006343	0.019423
MSTRG.11476	--	1.4875	0.695	-1.0978	0.006508	0.019873
ncbi_1439	CSF2RB	0.025	0.001	-4.64386	0.006583	0.020065
ncbi_81491	GPR63	0.0175	0.0925	2.402098	0.006902	0.02088
ncbi_5880	RAC2	0.1725	0.0175	-3.30117	0.007042	0.02124
MSTRG.4450	--	2.9	1.4475	-1.00249	0.007128	0.021457
ncbi_5744	PTHLH	0.6275	0.28	-1.16419	0.007157	0.021535
ncbi_112268350	LOC112268350	0.06	0.18	1.584963	0.00723	0.021732
ncbi_619189	SERINC4	0.19	0.405	1.091922	0.007343	0.022029

ID	Symbol	CK mean	T mean	Log2(FC)	P-value	FDR
ncbi_11131	CAPN11	0.4625	0.2125	-1.12199	0.00749	0.022404
ncbi_90853	SPOCD1	0.0625	0.165	1.400538	0.007515	0.022461
ncbi_640	BLK	0.001	0.0525	5.714246	0.007651	0.022817
ncbi_4753	NELL2	0.001	0.03	4.906891	0.007659	0.022829
ncbi_18	ABAT	0.0975	0.215	1.140863	0.007689	0.022895
ncbi_145447	ABHD12B	0.3	0.08	-1.90689	0.007762	0.02307
ncbi_440021	KRTAP5-2	0.72	0.3325	-1.11464	0.007933	0.023518
ncbi_107987276	LOC107987276	0.001	0.0575	5.84549	0.007951	0.023563
ncbi_7429	VIL1	0.0625	0.015	-2.05889	0.008036	0.023769
ncbi_107080638	TBC1D7-LOC100130357	0.001	0.235	7.876517	0.008078	0.023882
ncbi_2766	GMPR	0.095	0.001	-6.56986	0.00809	0.023908
MSTRG.17423	NOMO2	0.035	3.545	6.662287	0.008102	0.023933
ncbi_5798	PTPRN	0.1	0.03	-1.73697	0.008112	0.023954
ncbi_4939	OAS2	0.05	0.0025	-4.32193	0.008128	0.023982
ncbi_114900	C1QTNF4	0.095	0.26	1.452512	0.008285	0.024371
ncbi_389602	LOC389602	0.045	0.001	-5.49185	0.00831	0.024435
ncbi_64063	PRSS22	0.415	0.18	-1.20511	0.008575	0.025078
ncbi_63036	CELA2A	0.0625	0.305	2.286881	0.008632	0.025218
MSTRG.649	--	1.9525	0.9575	-1.02798	0.008655	0.025282
ncbi_145781	GCOM1	0.545	0.2475	-1.13883	0.008677	0.025333
ncbi_11240	PADI2	0.09	0.0225	-2	0.008901	0.025914
ncbi_731220	RFX8	0.13	0.0225	-2.53051	0.00894	0.026018
ncbi_2099	ESR1	0.2075	0.0825	-1.33065	0.008954	0.026056
ncbi_3485	IGFBP2	0.0825	0.001	-6.36632	0.008971	0.026094
ncbi_23563	CHST5	0.1575	0.335	1.088809	0.00908	0.026347
ncbi_65268	WNK2	0.0325	0.1	1.621488	0.009203	0.026659
ncbi_145501	ISM2	0.305	0.1225	-1.31603	0.009255	0.026782
ncbi_51481	VCX3A	0.1275	0.001	-6.99435	0.009277	0.026829
ncbi_4846	NOS3	0.07	0.01	-2.80735	0.009412	0.02718
ncbi_119	ADD2	0.025	0.075	1.584963	0.009459	0.027288
ncbi_1056	CEL	0.21	0.4575	1.123382	0.009524	0.027439
ncbi_5317	PKP1	0.07	0.0225	-1.63743	0.00953	0.027452
ncbi_389840	MAP3K15	0.0875	0.0275	-1.66985	0.009583	0.027589
ncbi_342918	C19orf81	0.395	0.945	1.258462	0.00964	0.027735
ncbi_534	ATP6V1G2	0.56	0.2175	-1.36441	0.009717	0.027922
ncbi_3790	KCNS3	0.14	0.3325	1.247928	0.009953	0.028515
ncbi_100533106	ZHX1-C8orf76	0.5125	1.255	1.292063	0.010099	0.028877
MSTRG.9638	--	0.105	0.785	2.902303	0.010116	0.028913
ncbi_138065	RNF183	0.001	0.04	5.321928	0.010405	0.029628
ncbi_90249	UNC5A	0.1075	0.24	1.158698	0.010414	0.029647
ncbi_339184	CCDC144NL	0.0475	0.001	-5.56986	0.010512	0.029869
ncbi_343521	TCTEX1D4	0.47	0.19	-1.30666	0.010551	0.029967
ncbi_163175	LGI4	0.065	0.1975	1.603341	0.010572	0.030017
ncbi_27290	SPINK4	0.31	0.001	-8.27612	0.010655	0.030221
ncbi_11272	PRR4	1.755	0.72	-1.2854	0.010768	0.03049
ncbi_220108	FAM124A	0.2275	0.4625	1.023587	0.010812	0.030598
ncbi_6367	CCL22	0.1075	0.0225	-2.25634	0.010846	0.030675
MSTRG.11877	--	0.405	1.28	1.66015	0.010861	0.030712
ncbi_55815	TSNAXIP1	0.2775	0.1225	-1.17971	0.010961	0.030939
ncbi_113220	KIF12	0.3775	0.1875	-1.00959	0.011162	0.031393
ncbi_50831	TAS2R3	0.06	0.3225	2.426265	0.011209	0.031488
ncbi_90527	DUOXA1	0.06	0.2175	1.857981	0.011235	0.031552
ncbi_50940	PDE11A	0.03	0.0025	-3.58496	0.011265	0.031597

ID	Symbol	CK mean	T mean	Log2(FC)	P-value	FDR
ncbi_1558	CYP2C8	0.105	0.0175	-2.58496	0.011632	0.032492
ncbi_11076	TPPP	4.7525	12.85	1.43501	0.011673	0.032602
ncbi_27129	HSPB7	54.55	19.5875	-1.47765	0.01195	0.033306
MSTRG.16952	--	0.295	0.8175	1.470504	0.01203	0.033493
ncbi_284340	CXCL17	0.11	0.001	-6.78136	0.012134	0.033749
ncbi_939	CD27	0.335	0.7425	1.14823	0.012148	0.033769
MSTRG.17603	NEK4	0.1075	0.01	-3.42626	0.012168	0.033811
ncbi_64170	CARD9	0.385	0.1725	-1.15826	0.012183	0.033847
MSTRG.6265	--	0.25	0.67	1.422233	0.012487	0.034566
ncbi_4600	MX2	0.0575	0.005	-3.52356	0.01251	0.034618
ncbi_2263	FGFR2	0.1425	0.05	-1.51096	0.012547	0.034693
ncbi_27254	CSDC2	0.245	0.0975	-1.32931	0.012733	0.035119
ncbi_201181	ZNF385C	0.0775	0.2075	1.420843	0.012773	0.035225
ncbi_151516	ASPRV1	0.2225	0.07	-1.66838	0.012857	0.03541
MSTRG.9836	OR1F12	0.71	1.8675	1.395217	0.012877	0.035442
ncbi_1805	DPT	0.0575	0.001	-5.84549	0.012949	0.035629
ncbi_284021	MILR1	0.3825	0.1425	-1.4245	0.013037	0.035846
MSTRG.265	UTY	1.16	0.5075	-1.19265	0.013401	0.036702
ncbi_152098	ZCWPW2	0.07	0.175	1.321928	0.013426	0.036759
ncbi_729220	FLJ45513	0.1575	0.0525	-1.58496	0.013542	0.037034
ncbi_55733	HHAT	0.185	0.08	-1.20945	0.01355	0.037051
ncbi_107984345	SMIM38	0.05	0.1825	1.867896	0.013632	0.037258
ncbi_7857	SCG2	0.25	0.1125	-1.152	0.013827	0.037697
ncbi_150248	C22orf15	0.1	0.325	1.70044	0.014001	0.038089
MSTRG.8292	HSD17B7	0.755	1.765	1.22512	0.014158	0.038431
ncbi_100526664	LY75-CD302	0.0025	0.0325	3.70044	0.014315	0.038814
ncbi_1281	COL3A1	0.05	0.015	-1.73697	0.014612	0.039544
MSTRG.6747	TCAF1	0.1675	0.4975	1.570535	0.014694	0.039747
ncbi_84679	SLC9A7	0.0075	0.0375	2.321928	0.014731	0.039826
ncbi_81706	PPP1R14C	0.105	0.025	-2.07039	0.014772	0.039911
ncbi_84443	FRMPD3	0.0175	0.001	-4.12928	0.015015	0.040451
ncbi_1082	CGB3	0.12	0.001	-6.90689	0.01502	0.040459
ncbi_563	AZGP1	0.0725	0.001	-6.17991	0.01517	0.040784
MSTRG.16851	--	1.085	0.29	-1.90357	0.015315	0.041109
ncbi_390877	LOC390877	0.47	0.095	-2.30666	0.015425	0.041352
ncbi_60385	TSKS	0.1225	0.3175	1.373975	0.015576	0.041716
ncbi_23581	CASP14	0.0925	0.0125	-2.88753	0.015869	0.042353
ncbi_8740	TNFSF14	0.225	0.1075	-1.06559	0.01588	0.042369
ncbi_8821	INPP4B	0.04	0.0075	-2.41504	0.016142	0.042971
ncbi_3625	INHBB	0.2025	0.08	-1.33985	0.016156	0.043002
ncbi_57578	UNC79	0.0125	0.0425	1.765535	0.016162	0.043012
ncbi_347051	SLC10A5	0.375	0.13	-1.52838	0.016274	0.043247
MSTRG.6862	--	0.5225	1.405	1.427067	0.016526	0.043808
ncbi_57188	ADAMTSL3	0.0375	0.01	-1.90689	0.017147	0.045104
ncbi_105373289	LOC105373289	0.0325	0.1525	2.230298	0.017194	0.045216
ncbi_4359	MPZ	0.465	0.23	-1.0156	0.01758	0.046087
ncbi_5724	PTAFR	0.09	0.02	-2.16993	0.017598	0.046116
ncbi_3885	KRT34	0.3675	0.165	-1.15528	0.017758	0.046464
ncbi_337959	KRTAP13-2	0.2025	0.03	-2.75489	0.017803	0.046562
MSTRG.3595	--	0.9	2.08	1.208587	0.017998	0.046975
ncbi_2124	EVI2B	0.001	0.06	5.906891	0.018009	0.046998
ncbi_5675	PSG6	0.0675	0.001	-6.07682	0.018018	0.047014
ncbi_54429	TAS2R5	0.1525	0.385	1.336049	0.01805	0.047084
MSTRG.3493	--	0.5475	1.16	1.083194	0.018072	0.047132

ID	Symbol	CK mean	T mean	Log2(FC)	P-value	FDR
ncbi_2824	GPM6B	0.1525	0.3925	1.363883	0.018144	0.047281
ncbi_79931	TNIP3	0.04	0.001	-5.32193	0.01815	0.047281
MSTRG.17214	DDX39B	0.1675	4.635	4.790336	0.018181	0.047352
ncbi_2662	GDF10	0.04	0.001	-5.32193	0.01821	0.047404
ncbi_1815	DRD4	0.06	0.2575	2.101538	0.018252	0.047483
ncbi_134526	ACOT12	0.04	0.1525	1.930737	0.018504	0.048067
ncbi_23779	ARHGAP8	0.0725	0.3175	2.130704	0.01868	0.048444
ncbi_8633	UNC5C	0.01	0.001	-3.32193	0.01877	0.048645
ncbi_2018	EMX2	0.0125	0.08	2.678072	0.018879	0.048874
ncbi_785	CACNB4	0.0125	0.0425	1.765535	0.019053	0.049249
ncbi_23302	WSCD1	0.0275	0.075	1.447459	0.019138	0.049418
ncbi_116372	LYPD1	0.3075	0.1	-1.62059	0.019156	0.049432
ncbi_6688	SPI1	0.08	0.215	1.426265	0.019283	0.049737
MSTRG.17426	PKD1	0.5625	0.03	-4.22882	0.019382	0.049916

CK, the control group; T, the naringenin group.

Table S2. Identification of 234 differentially expressed miRNAs in response to naringenin.

ID	CK mean	T mean	Log2(FC)	P-value	FDR
hsa-miR-100-3p	2.14815	0.722075	-1.57287	0.004131	0.096094
hsa-miR-10401-3p	53.77655	124.9839	1.216693	0.000653	0.026219
hsa-miR-1180-3p	186.0532	539.9781	1.537185	1.99E-08	7.38E-06
hsa-miR-1226-3p	14.05783	35.33723	1.329815	0.000351	0.016897
hsa-miR-1249-3p	59.0069	121.7634	1.045125	0.000554	0.023067
hsa-miR-1260b	135.0817	355.3202	1.395287	0.003389	0.08793
hsa-miR-1275	0.0891	1.962475	4.461105	0.001088	0.03821
hsa-miR-128-3p	1107.069	2263.123	1.03157	0.000487	0.02135
hsa-miR-1306-5p	8.03035	22.83505	1.507715	3.29E-06	0.000483
hsa-miR-1343-3p	22.18878	62.57825	1.495831	2.82E-06	0.000433
hsa-miR-1468-5p	2.696975	6.9443	1.364487	0.008559	0.148046
hsa-miR-181b-2-3p	2.8497	0.447975	-2.66932	2.18E-05	0.002162
hsa-miR-1908-5p	28.77723	84.29015	1.550437	2.75E-06	0.000433
hsa-miR-1910-5p	9.6821	21.62113	1.15905	0.000757	0.029019
hsa-miR-1914-5p	3.65005	8.7127	1.255204	0.013482	0.191339
hsa-miR-1915-5p	0.01	1.14025	6.833206	0.000722	0.028011
hsa-miR-194-3p	2.0881	0.775375	-1.42922	0.010298	0.162306
hsa-miR-194-5p	506.8478	244.0175	-1.05457	7.64E-09	4.30E-06
hsa-miR-200a-5p	0.5378	0.01	-5.749	0.039552	0.391227
hsa-miR-2116-3p	35.73298	80.51615	1.172022	0.000288	0.014708
hsa-miR-223-5p	2.304125	7.788175	1.757066	0.013146	0.188085
hsa-miR-2276-3p	0.103975	1.371175	3.721104	0.023199	0.280471
hsa-miR-2467-3p	0.498775	0.01	-5.64032	0.045758	0.427242
hsa-miR-27a-5p	332.9562	102.0299	-1.70634	2.20E-16	7.25E-13
hsa-miR-296-3p	7.691925	20.90015	1.442097	3.92E-05	0.003391
hsa-miR-296-5p	0.0877	1.19665	3.770281	0.005323	0.104638
hsa-miR-29b-1-5p	2.752325	1.36935	-1.00716	0.009129	0.154203
hsa-miR-3064-3p	0.01	0.864425	6.433669	0.014783	0.201876
hsa-miR-3140-5p	3.08135	6.82635	1.147552	0.0194	0.245006
hsa-miR-3176	6.490275	13.29788	1.034844	0.023986	0.286136
hsa-miR-3187-3p	1.9223	4.862275	1.338798	0.009194	0.154203
hsa-miR-320b	189.6155	380.9264	1.006435	0.00118	0.040614
hsa-miR-320c	58.93628	156.3999	1.408012	3.63E-06	0.00051
hsa-miR-320d	40.62008	118.4893	1.544491	1.58E-06	0.000266
hsa-miR-32-3p	6.4568	2.535225	-1.34871	5.97E-05	0.004576
hsa-miR-328-3p	12.15293	32.51398	1.419756	6.65E-06	0.000831

ID	CK mean	T mean	Log2(FC)	P-value	FDR
hsa-miR-342-5p	8.606125	17.47228	1.021632	0.037312	0.373456
hsa-miR-3622a-3p	0.53075	1.82165	1.779141	0.023311	0.280815
hsa-miR-3651	0.443375	2.6663	2.58824	0.003098	0.082287
hsa-miR-3935	6.2897	15.35375	1.287528	0.000346	0.016897
hsa-miR-3940-3p	10.8314	25.96798	1.261514	0.000386	0.018353
hsa-miR-429	0.9867	0.238075	-2.0512	0.031986	0.340281
hsa-miR-4435	2.4511	6.684475	1.447385	0.002374	0.06904
hsa-miR-4454	704.824	2902.649	2.042035	1.82E-05	0.001914
hsa-miR-4485-3p	5.633	13.84705	1.297603	0.004582	0.098361
hsa-miR-4661-5p	1.82025	4.976725	1.45106	0.004415	0.098361
hsa-miR-4707-3p	3.99775	8.121925	1.022633	0.027837	0.311943
hsa-miR-4731-5p	3.029225	7.265125	1.262039	0.013543	0.191339
hsa-miR-4743-5p	0.01	0.901325	6.493976	0.014193	0.195398
hsa-miR-4798-5p	1.499	0.4946	-1.59967	0.01316	0.188085
hsa-miR-484	2004.028	4043.176	1.012586	0.000516	0.022042
hsa-miR-519a-3p	1.255025	0.24565	-2.35304	0.007343	0.135351
hsa-miR-521	1.311525	0.29935	-2.13134	0.012358	0.181383
hsa-miR-548f-3p	2.605625	0.70715	-1.88154	0.000933	0.033824
hsa-miR-556-3p	0.760675	2.309125	1.601994	0.024766	0.29106
hsa-miR-627-3p	11.1459	23.39618	1.06976	0.00878	0.150679
hsa-miR-628-3p	57.70935	129.0516	1.16107	0.000966	0.034647
hsa-miR-6511a-3p	1.182325	3.4624	1.550146	0.00995	0.159815
hsa-miR-6516-5p	1.3841	0.24565	-2.49427	0.001401	0.046802
hsa-miR-6732-3p	0.01	1.21215	6.921424	0.000469	0.020834
hsa-miR-6741-3p	0.4657	2.2852	2.294848	0.009187	0.154203
hsa-miR-676-3p	0.7146	0.01	-6.15906	0.004077	0.095492
hsa-miR-6786-3p	0.01	0.6101	5.930974	0.033025	0.345111
hsa-miR-6837-5p	0.7511	0.122575	-2.61534	0.035207	0.360953
hsa-miR-7-5p	1860.868	448.2273	-2.05367	4.30E-16	7.25E-13
hsa-miR-7704	0.51185	2.148375	2.069453	0.011588	0.174714
hsa-miR-7854-3p	2.7247	6.428	1.238273	0.010477	0.162855
hsa-miR-7974	57.8125	20.31288	-1.50899	5.37E-07	0.000101
hsa-miR-7976	35.86573	72.9189	1.023687	0.002802	0.075544
hsa-miR-877-5p	11.97035	24.90598	1.057027	0.005336	0.104638
hsa-miR-890	5.8411	2.1765	-1.42423	6.55E-05	0.004913
hsa-miR-93-3p	100.5085	224.3599	1.158498	0.000589	0.023949
hsa-miR-935	4.697775	11.0814	1.238091	0.004029	0.095023
miR-10240-x	0.522925	3.246375	2.634154	0.007702	0.141188
miR-10285-x	0.01	0.72495	6.17981	0.006142	0.117042
miR-10401-y	1.2546	3.561175	1.505126	0.024689	0.29106
miR-10555-x	45.17148	101.1659	1.163238	0.012368	0.181383
miR-10955-y	278.1233	817.4349	1.555379	0.000222	0.011895
miR-11202-x	38.56388	109.9068	1.510959	0.003455	0.088967
miR-1180-y	6.562375	13.98235	1.091317	0.032702	0.344704
miR-11975-x	0.01	1.069675	6.741029	0.001677	0.05257
miR-11976-y	0.01	1.069675	6.741029	0.001672	0.05257
miR-1249-y	3.5482	12.77828	1.848534	5.87E-06	0.000761
miR-1260-x	123.2804	359.4877	1.543999	1.94E-05	0.001979
miR-126-y	18.33783	53.0677	1.533011	0.029558	0.325243
miR-1275-x	1.605	13.9966	3.124431	1.11E-07	2.34E-05
miR-127-y	0.01	0.629025	5.975045	0.049907	0.435859
miR-1283-x	1.174925	0.34205	-1.78029	0.036076	0.365545
miR-1304-y	40.39605	90.53423	1.164249	0.00019	0.010474
miR-1343-y	0.191675	2.2832	3.574323	2.82E-05	0.002572

ID	CK mean	T mean	Log2(FC)	P-value	FDR
miR-1346-y	0.6543	2.3744	1.859539	0.017823	0.22858
miR-1386-x	125.6883	296.2251	1.236843	0.005752	0.111123
miR-139-x	0.9457	2.45055	1.373651	0.046771	0.431036
miR-143-y	12.0704	34.14153	1.500054	0.025457	0.295073
miR-146-x	841.6046	2159.469	1.359462	0.03562	0.362983
miR-1599-y	0.8321	13.61488	4.032283	1.77E-15	1.99E-12
miR-193-y	31.24883	64.3569	1.042293	0.001908	0.058295
miR-194-x	21.32845	5.97975	-1.83462	1.05E-11	8.82E-09
miR-195-x	2.771	1.33885	-1.04941	0.010848	0.167355
miR-197-y	7.834975	20.67135	1.399632	0.000149	0.008962
miR-2116-y	7.462975	18.86575	1.337947	0.003491	0.089214
miR-2137-y	0.579725	0.01	-5.8573	0.0088	0.150679
miR-223-x	1.699275	6.1853	1.863924	0.012759	0.184566
miR-223-y	1.34505	5.2113	1.953984	0.019663	0.246555
miR-235-y	56.21323	119.156	1.08387	0.000698	0.027393
miR-2404-x	2.579925	6.3084	1.289945	0.031422	0.337534
miR-2478-y	4932.054	11456.96	1.215964	0.000411	0.019267
miR-2779-y	181.6928	457.8107	1.33325	0.015299	0.20641
miR-2779-z	464.3346	1369.374	1.56028	0.000501	0.02167
miR-301-y	19.3394	7.5896	-1.34945	5.56E-05	0.004468
miR-310-y	1.2374	4.15385	1.747137	0.004026	0.095023
miR-3123-y	0.155975	1.432825	3.199476	0.019467	0.245006
miR-3186-y	0.562925	0.01	-5.81487	0.029315	0.325243
miR-3187-y	0.701775	2.829	2.011212	0.009709	0.1582
miR-31-y	0.53075	0.01	-5.72996	0.014627	0.200555
miR-338-y	24.76018	8.62935	-1.5207	2.37E-08	7.38E-06
miR-340-x	1.90695	0.6101	-1.64415	0.009436	0.156016
miR-3934-x	1.265075	0.537025	-1.23616	0.044392	0.426593
miR-3940-y	0.32325	1.651025	2.35264	0.032182	0.340281
miR-3960-y	2.0273	4.798725	1.243092	0.03104	0.336017
miR-3963-x	3.507525	11.96458	1.770244	2.38E-05	0.002289
miR-3968-y	3775.769	9178.515	1.28149	0.000543	0.022888
miR-4286-y	3.107025	8.46805	1.446496	0.008205	0.148046
miR-4286-z	6.149275	14.766	1.263791	0.021445	0.264961
miR-4425-y	1.1684	0.29935	-1.96463	0.0247	0.29106
miR-4443-x	2.8821	8.9895	1.64112	0.002024	0.060953
miR-4447-y	55.75503	27.27125	-1.03172	0.000172	0.009977
miR-4483-y	88.52515	24.97935	-1.82535	1.58E-09	1.07E-06
miR-4485-y	1.6544	7.369175	2.155195	0.000145	0.008897
miR-4516-x	0.648	2.9477	2.185524	0.001825	0.056473
miR-451-x	5.606025	16.30298	1.540085	0.036155	0.365545
miR-466-y	0.01	0.95595	6.578863	0.002345	0.068776
miR-5119-y	0.01	1.6604	7.375387	1.26E-05	0.001414
miR-542-y	4.7206	1.57425	-1.58431	0.00012	0.007754
miR-615-y	52.91628	154.1604	1.542649	1.58E-07	3.13E-05
miR-6240-x	8.6868	29.60528	1.768958	0.000111	0.007357
miR-6412-y	0.6331	2.25275	1.831182	0.0089	0.151616
miR-671-x	4.160475	8.9253	1.101152	0.044858	0.427242
miR-709-y	0.516325	4.203325	3.02518	8.90E-05	0.006061
miR-7550-x	615.2486	1675.787	1.445598	0.005042	0.100046
miR-7669-y	0.01	0.537025	5.746917	0.031059	0.336017
miR-7792-y	3.802225	12.97155	1.770435	0.001023	0.036314
miR-7977-x	43.3792	107.7896	1.313142	0.002429	0.070027
miR-7-x	6.398	1.84115	-1.79701	0.000298	0.014815

ID	CK mean	T mean	Log2(FC)	P-value	FDR
miR-8112-y	4.637425	12.855	1.470934	0.002812	0.075544
miR-8528-x	7.151275	2.8407	-1.33195	0.003671	0.089819
miR-873-x	0.43535	2.1977	2.335747	0.005524	0.107697
miR-8824-y	1.22785	4.996775	2.024863	0.000135	0.008439
miR-9226-y	34.2145	97.70465	1.513819	0.001111	0.038632
miR-9277-y	125.8135	491.2266	1.965102	9.50E-07	0.000169
miR-939-x	0.0891	1.10115	3.627442	0.031913	0.340281
miR-93-y	9.779	30.42418	1.637459	0.000444	0.020454
miR-96-x	10.79435	4.65505	-1.21341	8.35E-06	0.000971
miR-9993-y	315.8498	664.1963	1.072371	0.013613	0.191339
novel-m0013-5p	4.538725	10.73825	1.2424	0.004741	0.098361
novel-m0049-5p	0.268175	2.8005	3.384438	0.004872	0.098361
novel-m0050-5p	0.268175	2.8005	3.384438	0.004878	0.098361
novel-m0051-5p	0.268175	2.8005	3.384438	0.004866	0.098361
novel-m0052-5p	0.268175	2.8005	3.384438	0.004848	0.098361
novel-m0053-3p	0.0877	0.890875	3.344574	0.043786	0.424401
novel-m0067-3p	0.822025	0.122575	-2.74552	0.024509	0.29106
novel-m0069-5p	4.538725	10.73825	1.2424	0.004724	0.098361
novel-m0079-3p	0.5927	2.83735	2.25917	0.01835	0.233568
novel-m0081-5p	0.758575	0.01	-6.24522	0.003595	0.089819
novel-m0084-5p	0.268175	2.8005	3.384438	0.004804	0.098361
novel-m0086-5p	0.268175	2.8005	3.384438	0.00476	0.098361
novel-m0090-5p	0.01	1.324125	7.048896	0.000179	0.01004
novel-m0093-5p	0.268175	2.8005	3.384438	0.00473	0.098361
novel-m0095-5p	1.097675	4.638225	2.079122	8.96E-05	0.006061
novel-m0111-5p	0.01	0.659875	6.044121	0.011603	0.174714
novel-m0119-3p	34.55555	70.9688	1.038267	0.021536	0.265117
novel-m0124-5p	0.01	0.870425	6.443648	0.011069	0.16971
novel-m0126-5p	0.268175	2.8005	3.384438	0.004692	0.098361
novel-m0142-5p	0.268175	2.8005	3.384438	0.004656	0.098361
novel-m0157-5p	1.6948	0.7514	-1.17346	0.046745	0.431036
novel-m0159-5p	6.23455	3.080775	-1.01699	0.000455	0.020454
novel-m0169-5p	0.268175	2.8005	3.384438	0.004637	0.098361
novel-m0186-5p	0.38805	0.01	-5.27817	0.03315	0.345111
novel-m0209-3p	0.872575	2.707	1.633344	0.043583	0.423645
novel-m0212-5p	0.884875	2.87795	1.701496	0.01764	0.227967
novel-m0214-3p	0.95255	2.496375	1.389968	0.044861	0.427242
novel-m0224-5p	0.4657	1.99905	2.101842	0.008557	0.148046
novel-m0225-5p	0.4657	1.99905	2.101842	0.008505	0.148046
novel-m0226-5p	0.4657	1.99905	2.101842	0.008465	0.148046
novel-m0237-5p	0.4657	1.99905	2.101842	0.00845	0.148046
novel-m0238-5p	0.4657	1.99905	2.101842	0.008397	0.148046
novel-m0245-3p	6.95505	14.0438	1.013801	0.015864	0.213188
novel-m0249-5p	4.957275	12.3077	1.311942	0.000583	0.023949
novel-m0275-5p	0.01	0.9468	6.564988	0.012066	0.179957
novel-m0291-5p	0.01	1.324125	7.048896	0.000177	0.01004
novel-m0292-5p	0.01	0.9468	6.564988	0.012036	0.179957
novel-m0297-5p	0.51185	0.01	-5.67765	0.047314	0.432495
novel-m0298-3p	1.1021	2.766	1.327546	0.033762	0.350398
novel-m0328-3p	0.514475	0.01	-5.68503	0.03498	0.35972
novel-m0345-5p	0.38805	0.01	-5.27817	0.033109	0.345111
novel-m0351-5p	0.01	0.68225	6.092229	0.025697	0.29684
novel-m0367-5p	1.81985	0.7205	-1.33675	0.021656	0.265617
novel-m0370-5p	0.268175	2.8005	3.384438	0.004617	0.098361

ID	CK mean	T mean	Log2(FC)	P-value	FDR
novel-m0390-3p	0.586325	0.01	-5.87363	0.025904	0.297195
novel-m0424-5p	0.569675	0.01	-5.83207	0.024962	0.291333
novel-m0441-5p	1.606275	5.35635	1.737531	0.001918	0.058295
novel-m0452-5p	1.994775	0.18385	-3.43962	0.003167	0.08345
novel-m0476-3p	0.01	0.59505	5.894939	0.046057	0.427965
novel-m0485-5p	33645.49	13948.3	-1.27032	3.29E-08	7.39E-06
novel-m0487-3p	0.7654	2.91575	1.929581	0.048949	0.433345
novel-m0488-3p	0.7654	2.91575	1.929581	0.048759	0.432804
novel-m0494-3p	0.01	0.59505	5.894939	0.045853	0.427242
novel-m0506-3p	0.01	0.59505	5.894939	0.045775	0.427242
novel-m0516-5p	33645.49	13948.3	-1.27032	3.05E-08	7.38E-06
novel-m0517-3p	0.7654	2.91575	1.929581	0.048724	0.432804
novel-m0518-3p	0.7654	2.91575	1.929581	0.048664	0.432804
novel-m0519-3p	0.7654	2.91575	1.929581	0.048661	0.432804
novel-m0525-3p	0.01	0.59505	5.894939	0.045633	0.427242
novel-m0533-5p	33645.49	13948.3	-1.27032	2.98E-08	7.38E-06
novel-m0535-3p	0.7654	2.91575	1.929581	0.048597	0.432804
novel-m0536-3p	0.7654	2.91575	1.929581	0.048511	0.432804
novel-m0544-5p	1.506	0.66405	-1.18136	0.039486	0.391227
novel-m0558-5p	5.285525	2.638975	-1.00207	0.000923	0.033824
novel-m0583-5p	5.285525	2.638975	-1.00207	0.000919	0.033824
novel-m0609-5p	0.568125	0.01	-5.82814	0.024893	0.291333
novel-m0613-5p	0.268175	2.8005	3.384438	0.00458	0.098361
novel-m0614-5p	0.268175	2.8005	3.384438	0.004559	0.098361
novel-m0624-3p	0.01	0.59505	5.894939	0.045491	0.427242
novel-m0632-5p	33645.49	13948.3	-1.27032	2.99E-08	7.38E-06
novel-m0633-3p	0.7654	2.91575	1.929581	0.048329	0.432804
novel-m0639-3p	0.01	0.59505	5.894939	0.04535	0.427242
novel-m0647-5p	33645.49	13948.3	-1.27032	3.00E-08	7.38E-06
novel-m0672-3p	0.01	0.59505	5.894939	0.045211	0.427242
novel-m0680-5p	33645.49	13948.3	-1.27032	3.05E-08	7.38E-06
novel-m0682-3p	0.7654	2.91575	1.929581	0.048223	0.432804
novel-m0683-3p	0.7654	2.91575	1.929581	0.048119	0.432804
novel-m0690-3p	0.01	0.59505	5.894939	0.045058	0.427242
novel-m0698-5p	33645.49	13948.3	-1.27032	3.06E-08	7.38E-06
novel-m0699-5p	0.268175	2.8005	3.384438	0.004539	0.098361

CK, the control group; T, the naringenin group.

Table S3. Identification of 5607 negative miRNA-mRNA pairs in response to naringenin, with the involvement of 216 DEMs and 681 DEGs in total.

MiRNA	Target number	Target gene
hsa-miR-100-3p	18	ncbi_2824,ncbi_646851,ncbi_6752,ncbi_55186,ncbi_84439,ncbi_29767,ncbi_283710,ncbi_100506127,ncbi_340351,ncbi_7700,ncbi_8635,ncbi_9658,ncbi_56605,ncbi_134526,ncbi_136647,ncbi_266722,ncbi_130733,ncbi_10219
hsa-miR-10401-3p	14	ncbi_286,ncbi_339761,ncbi_388323,ncbi_27122,ncbi_7161,ncbi_91156,ncbi_5029,ncbi_6585,ncbi_27289,ncbi_23105,ncbi_51286,ncbi_127294,ncbi_4135,ncbi_1261
hsa-miR-1180-3p	4	ncbi_1435,ncbi_90427,ncbi_80274,ncbi_55106
hsa-miR-1226-3p	35	ncbi_1435,ncbi_90427,ncbi_4582,ncbi_220979,ncbi_54921,ncbi_55124,ncbi_5328,ncbi_440689,ncbi_7185,ncbi_8942,ncbi_54587,ncbi_83690,ncbi_140711,ncbi_150372,ncbi_8839,ncbi_6518,ncbi_2247,ncbi_138050,ncbi_3604,ncbi_6585,ncbi_9283,ncbi_3037,ncbi_7837,ncbi_30811,ncbi_23237,ncbi_56103,ncbi_56099,ncbi_10060,ncbi_79628,ncbi_147138,ncbi_4135,ncbi_1261,ncbi_256158,ncbi_2901,ncbi_107985729
hsa-miR-1249-3p	7	ncbi_3486,ncbi_64699,ncbi_389602,ncbi_5029,ncbi_9379,ncbi_64856,ncbi_145501

MiRNA	Target number	Target gene
hsa-miR-1260b	42	ncbi_2914,ncbi_5143,ncbi_389840,ncbi_51151,ncbi_6536,ncbi_4222,ncbi_26471,ncbi_141,ncbi_6932,ncbi_57571,ncbi_5137,ncbi_145447,ncbi_146547,ncbi_8638,ncbi_4261,ncbi_171024,ncbi_140731,ncbi_6518,ncbi_55124,ncbi_2247,ncbi_166336,ncbi_1805,ncbi_3014,ncbi_3371,ncbi_5652,ncbi_8740,ncbi_9507,ncbi_23581,ncbi_7462,ncbi_30811,ncbi_9770,ncbi_9379,ncbi_10060,ncbi_79442,ncbi_79628,ncbi_127294,ncbi_127707,ncbi_151516,ncbi_80274,ncbi_286204,ncbi_221527,ncbi_80008
hsa-miR-1275	74	ncbi_286,ncbi_2099,ncbi_2155,ncbi_2200,ncbi_1439,ncbi_3172,ncbi_5054,ncbi_3479,ncbi_7448,ncbi_90427,ncbi_2707,ncbi_440603,ncbi_643866,ncbi_8710,ncbi_116535,ncbi_7161,ncbi_6932,ncbi_284422,ncbi_9214,ncbi_55765,ncbi_5328,ncbi_53841,ncbi_56834,ncbi_100130827,ncbi_401934,ncbi_2494,ncbi_11148,ncbi_54587,ncbi_171024,ncbi_1436,ncbi_8764,ncbi_376267,ncbi_50632,ncbi_6518,ncbi_342667,ncbi_5159,ncbi_128414,ncbi_112694756,ncbi_8456,ncbi_166336,ncbi_3604,ncbi_2901,ncbi_5029,ncbi_6367,ncbi_6585,ncbi_1463,ncbi_9283,ncbi_7471,ncbi_4584,ncbi_11131,ncbi_30811,ncbi_51703,ncbi_8862,ncbi_55742,ncbi_56103,ncbi_56099,ncbi_10129,ncbi_114897,ncbi_84443,ncbi_90226,ncbi_80274,ncbi_348013,ncbi_4739,ncbi_5744,ncbi_286204,ncbi_55106,ncbi_5655,ncbi_339184,ncbi_4222,ncbi_9542,ncbi_9033,ncbi_23105,ncbi_1141,ncbi_25903
hsa-miR-128-3p	66	ncbi_1281,ncbi_2099,ncbi_3561,ncbi_3172,ncbi_3479,ncbi_1435,ncbi_164395,ncbi_51151,ncbi_6263,ncbi_1261,ncbi_56649,ncbi_6925,ncbi_768239,ncbi_116535,ncbi_8821,ncbi_347454,ncbi_141,ncbi_321,ncbi_4773,ncbi_9214,ncbi_55765,ncbi_8605,ncbi_221468,ncbi_7185,ncbi_5137,ncbi_2859,ncbi_26298,ncbi_145447,ncbi_834,ncbi_93082,ncbi_101928841,ncbi_112399,ncbi_10628,ncbi_2635,ncbi_84467,ncbi_58476,ncbi_55577,ncbi_55124,ncbi_79788,ncbi_138050,ncbi_1212,ncbi_445577,ncbi_283298,ncbi_3604,ncbi_2920,ncbi_8519,ncbi_8633,ncbi_8740,ncbi_9283,ncbi_4584,ncbi_55742,ncbi_57462,ncbi_81606,ncbi_81706,ncbi_84962,ncbi_117157,ncbi_119467,ncbi_79987,ncbi_4135,ncbi_55106,ncbi_23452,ncbi_3965,ncbi_9542,ncbi_105369535,ncbi_79895,ncbi_6578
hsa-miR-1306-5p	40	ncbi_3172,ncbi_1141,ncbi_2562,ncbi_2914,ncbi_5724,ncbi_26471,ncbi_768239,ncbi_124976,ncbi_4773,ncbi_8542,ncbi_731220,ncbi_221468,ncbi_647024,ncbi_2521,ncbi_124590,ncbi_389602,ncbi_3669,ncbi_101928841,ncbi_27071,ncbi_112399,ncbi_10628,ncbi_163782,ncbi_105375355,ncbi_5159,ncbi_1735,ncbi_112694756,ncbi_283298,ncbi_2901,ncbi_4939,ncbi_5579,ncbi_1463,ncbi_90226,ncbi_254228,ncbi_80274,ncbi_128209,ncbi_286204,ncbi_348013,ncbi_56606,ncbi_153478,ncbi_8740
hsa-miR-1343-3p	81	ncbi_94,ncbi_286,ncbi_554,ncbi_2263,ncbi_4261,ncbi_3172,ncbi_2006,ncbi_6556,ncbi_624,ncbi_5143,ncbi_339761,ncbi_284434,ncbi_164395,ncbi_4939,ncbi_6531,ncbi_50940,ncbi_5655,ncbi_124976,ncbi_7161,ncbi_171024,ncbi_321,ncbi_131578,ncbi_11309,ncbi_643382,ncbi_647024,ncbi_56834,ncbi_8479,ncbi_146547,ncbi_56936,ncbi_84699,ncbi_51421,ncbi_53615,ncbi_2044,ncbi_4815,ncbi_285489,ncbi_140711,ncbi_50632,ncbi_2099,ncbi_55577,ncbi_84941,ncbi_3604,ncbi_1298,ncbi_3371,ncbi_4600,ncbi_6585,ncbi_7134,ncbi_4606,ncbi_4868,ncbi_3702,ncbi_3108,ncbi_10893,ncbi_11131,ncbi_25759,ncbi_30811,ncbi_9915,ncbi_23105,ncbi_23237,ncbi_55742,ncbi_56103,ncbi_56099,ncbi_60676,ncbi_57462,ncbi_64856,ncbi_10129,ncbi_84443,ncbi_153478,ncbi_126868,ncbi_51435,ncbi_100996758,ncbi_2902,ncbi_3965,ncbi_2901,ncbi_2570,ncbi_5540,ncbi_6932,ncbi_57571,ncbi_25903,ncbi_729857,ncbi_3479,ncbi_55106,ncbi_147138
hsa-miR-1468-5p	6	ncbi_1141,ncbi_8942,ncbi_58476,ncbi_166336,ncbi_51351,ncbi_10129
hsa-miR-181b-2-3p	31	ncbi_5896,ncbi_286223,ncbi_401265,ncbi_9901,ncbi_6752,ncbi_285268,ncbi_6565,ncbi_220108,ncbi_388650,ncbi_84253,ncbi_10219,ncbi_7700,ncbi_84449,ncbi_100129924,ncbi_1950,ncbi_119,ncbi_8635,ncbi_4323,ncbi_9745,ncbi_55150,ncbi_56648,ncbi_84249,ncbi_136647,ncbi_91749,ncbi_57161,ncbi_23418,ncbi_79846,ncbi_10004,ncbi_55840,ncbi_107984859,ncbi_100506127
hsa-miR-1908-5p	52	ncbi_286,ncbi_6469,ncbi_3691,ncbi_3914,ncbi_3172,ncbi_2006,ncbi_4359,ncbi_6556,ncbi_3589,ncbi_1141,ncbi_2902,ncbi_3560,ncbi_5143,ncbi_7071,ncbi_285489,ncbi_9542,ncbi_8942,ncbi_84699,ncbi_100506115,ncbi_53615,ncbi_395,ncbi_6512,ncbi_102

MiRNA	Target number	Target gene
		800317,ncbi_8764,ncbi_150372,ncbi_50632,ncbi_27129,ncbi_100129484,ncbi_3014,ncbi_5029,ncbi_5105,ncbi_6367,ncbi_9283,ncbi_2810,ncbi_10893,ncbi_23105,ncbi_23237,ncbi_23371,ncbi_55084,ncbi_64063,ncbi_64856,ncbi_251,ncbi_64005,ncbi_117157,ncbi_286204,ncbi_55765,ncbi_56606,ncbi_153478,ncbi_3965,ncbi_343990,ncbi_64284,ncbi_5745
hsa-miR-1910-5p	30	ncbi_94,ncbi_1439,ncbi_7066,ncbi_90427,ncbi_79098,ncbi_7430,ncbi_7161,ncbi_63901,ncbi_4015,ncbi_56936,ncbi_4188,ncbi_195814,ncbi_150372,ncbi_3965,ncbi_105375355,ncbi_27129,ncbi_3625,ncbi_4606,ncbi_7429,ncbi_25759,ncbi_9915,ncbi_26040,ncbi_55107,ncbi_79817,ncbi_127707,ncbi_80274,ncbi_166336,ncbi_55084,ncbi_25903,ncbi_8942
hsa-miR-1914-5p	26	ncbi_2914,ncbi_3560,ncbi_164395,ncbi_388595,ncbi_653145,ncbi_1261,ncbi_728113,ncbi_7161,ncbi_321,ncbi_338382,ncbi_1520,ncbi_56936,ncbi_84467,ncbi_1212,ncbi_285966,ncbi_3575,ncbi_30811,ncbi_51351,ncbi_83729,ncbi_151516,ncbi_171483,ncbi_4135,ncbi_2902,ncbi_5239,ncbi_1141,ncbi_343990
hsa-miR-1915-5p	21	ncbi_5317,ncbi_1734,ncbi_7161,ncbi_6932,ncbi_729220,ncbi_10752,ncbi_53615,ncbi_150372,ncbi_58476,ncbi_27129,ncbi_5029,ncbi_3702,ncbi_54625,ncbi_57530,ncbi_64856,ncbi_83729,ncbi_171483,ncbi_2323,ncbi_84814,ncbi_4599,ncbi_105373347
hsa-miR-194-3p	14	ncbi_284194,ncbi_84439,ncbi_2244,ncbi_84679,ncbi_100130370,ncbi_65268,ncbi_1645,ncbi_9717,ncbi_55586,ncbi_56605,ncbi_285349,ncbi_113451,ncbi_339541,ncbi_105373289
hsa-miR-194-5p	18	ncbi_388650,ncbi_151556,ncbi_285755,ncbi_100526664,ncbi_25850,ncbi_619189,ncbi_144321,ncbi_23349,ncbi_9745,ncbi_340351,ncbi_107984345,ncbi_9658,ncbi_25956,ncbi_57161,ncbi_136647,ncbi_91749,ncbi_84700,ncbi_266722
hsa-miR-200a-5p	8	ncbi_2824,ncbi_84679,ncbi_9331,ncbi_7700,ncbi_51554,ncbi_56648,ncbi_84071,ncbi_100289279
hsa-miR-2116-3p	41	ncbi_5317,ncbi_7066,ncbi_5054,ncbi_4939,ncbi_6241,ncbi_645121,ncbi_169270,ncbi_6531,ncbi_79098,ncbi_55733,ncbi_141,ncbi_91156,ncbi_285489,ncbi_7433,ncbi_8835,ncbi_2494,ncbi_9415,ncbi_140731,ncbi_10628,ncbi_163782,ncbi_2247,ncbi_1735,ncbi_138050,ncbi_445577,ncbi_3575,ncbi_5579,ncbi_5971,ncbi_27289,ncbi_30811,ncbi_9770,ncbi_51435,ncbi_8862,ncbi_10060,ncbi_79442,ncbi_3371,ncbi_55106,ncbi_23452,ncbi_55084,ncbi_9542,ncbi_653145,ncbi_3479
hsa-miR-223-5p	17	ncbi_2562,ncbi_5143,ncbi_169270,ncbi_26471,ncbi_8626,ncbi_4773,ncbi_5137,ncbi_59350,ncbi_3773,ncbi_84699,ncbi_389602,ncbi_1634,ncbi_4600,ncbi_8740,ncbi_23452,ncbi_30811,ncbi_51702
hsa-miR-2276-3p	29	ncbi_5317,ncbi_2562,ncbi_5724,ncbi_4939,ncbi_26471,ncbi_50940,ncbi_116535,ncbi_149461,ncbi_141,ncbi_1780,ncbi_8542,ncbi_285489,ncbi_5137,ncbi_57535,ncbi_283229,ncbi_25903,ncbi_101928841,ncbi_1396,ncbi_150372,ncbi_55577,ncbi_1634,ncbi_30811,ncbi_9770,ncbi_23237,ncbi_55561,ncbi_23452,ncbi_4599,ncbi_1141,ncbi_57188
hsa-miR-2467-3p	23	ncbi_785,ncbi_646851,ncbi_80852,ncbi_55186,ncbi_285755,ncbi_84439,ncbi_23213,ncbi_5346,ncbi_84679,ncbi_23349,ncbi_640,ncbi_102724488,ncbi_119,ncbi_9095,ncbi_11076,ncbi_29802,ncbi_23302,ncbi_25956,ncbi_9050,ncbi_285349,ncbi_84700,ncbi_113451,ncbi_266722
hsa-miR-27a-5p	7	ncbi_9901,ncbi_6688,ncbi_29767,ncbi_84249,ncbi_128434,ncbi_285349,ncbi_90249
hsa-miR-3651	14	ncbi_1141,ncbi_1734,ncbi_2786,ncbi_8605,ncbi_4846,ncbi_146547,ncbi_2044,ncbi_140711,ncbi_166336,ncbi_10129,ncbi_79628,ncbi_84962,ncbi_23452,ncbi_114769
hsa-miR-3935	21	ncbi_3479,ncbi_5724,ncbi_2707,ncbi_5047,ncbi_8710,ncbi_338382,ncbi_171024,ncbi_5271,ncbi_4148,ncbi_5744,ncbi_10274,ncbi_7429,ncbi_55742,ncbi_57462,ncbi_79413,ncbi_79628,ncbi_254228,ncbi_23452,ncbi_8942,ncbi_51435,ncbi_57188
hsa-miR-3940-3p	23	ncbi_2155,ncbi_1141,ncbi_3172,ncbi_768239,ncbi_2786,ncbi_397,ncbi_146547,ncbi_150372,ncbi_50632,ncbi_84941,ncbi_100129484,ncbi_5159,ncbi_9507,ncbi_3005,ncbi_9568,ncbi_8971,ncbi_23452,ncbi_55561,ncbi_153478,ncbi_127707,ncbi_80274,ncbi_25759,ncbi_653145

MiRNA	Target number	Target gene
hsa-miR-429	32	ncbi_5896,ncbi_18,ncbi_401265,ncbi_283726,ncbi_646851,ncbi_9901,ncbi_285268,ncbi_728378,ncbi_55186,ncbi_23213,ncbi_1638,ncbi_259308,ncbi_81491,ncbi_6565,ncbi_130733,ncbi_84071,ncbi_9331,ncbi_9745,ncbi_340351,ncbi_102724488,ncbi_5577,ncbi_8635,ncbi_9658,ncbi_56648,ncbi_57161,ncbi_9050,ncbi_80032,ncbi_285349,ncbi_266722,ncbi_785,ncbi_55840,ncbi_100506127
hsa-miR-4707-3p	25	ncbi_286,ncbi_6556,ncbi_5143,ncbi_50940,ncbi_4648,ncbi_8542,ncbi_285489,ncbi_5798,ncbi_342667,ncbi_6585,ncbi_9283,ncbi_51702,ncbi_55561,ncbi_56103,ncbi_56099,ncbi_56901,ncbi_64856,ncbi_84814,ncbi_112464,ncbi_127294,ncbi_283229,ncbi_348013,ncbi_4739,ncbi_2902,ncbi_166336
hsa-miR-4731-5p	108	ncbi_94,ncbi_554,ncbi_5317,ncbi_6556,ncbi_7448,ncbi_2563,ncbi_2902,ncbi_2914,ncbi_3560,ncbi_440603,ncbi_6536,ncbi_4222,ncbi_26471,ncbi_79919,ncbi_149461,ncbi_124976,ncbi_6932,ncbi_131578,ncbi_4776,ncbi_8542,ncbi_55765,ncbi_387763,ncbi_11309,ncbi_9058,ncbi_647024,ncbi_79674,ncbi_5137,ncbi_4057,ncbi_1520,ncbi_100130827,ncbi_30851,ncbi_56936,ncbi_84699,ncbi_2494,ncbi_100506115,ncbi_51421,ncbi_9415,ncbi_83882,ncbi_8764,ncbi_340061,ncbi_3669,ncbi_140711,ncbi_4359,ncbi_84467,ncbi_58476,ncbi_27129,ncbi_100129484,ncbi_5159,ncbi_1735,ncbi_138050,ncbi_3625,ncbi_3725,ncbi_5105,ncbi_5880,ncbi_6585,ncbi_8581,ncbi_8633,ncbi_9037,ncbi_9283,ncbi_4584,ncbi_11131,ncbi_23581,ncbi_27076,ncbi_27254,ncbi_30811,ncbi_23105,ncbi_23237,ncbi_23371,ncbi_8862,ncbi_55561,ncbi_55084,ncbi_55107,ncbi_56103,ncbi_56099,ncbi_56241,ncbi_64063,ncbi_79442,ncbi_79628,ncbi_7473,ncbi_81606,ncbi_114897,ncbi_90226,ncbi_153478,ncbi_117157,ncbi_127707,ncbi_1435,ncbi_80274,ncbi_128209,ncbi_286204,ncbi_91947,ncbi_23452,ncbi_285489,ncbi_56606,ncbi_3134,ncbi_53841,ncbi_3965,ncbi_2901,ncbi_128414,ncbi_1439,ncbi_166336,ncbi_7433,ncbi_1141,ncbi_25903,ncbi_107985729,ncbi_343990,ncbi_5745,ncbi_8456,ncbi_80008
hsa-miR-4743-5p	16	ncbi_286,ncbi_2902,ncbi_79919,ncbi_7161,ncbi_285489,ncbi_7185,ncbi_284297,ncbi_101928841,ncbi_727897,ncbi_23237,ncbi_26040,ncbi_153478,ncbi_348013,ncbi_4261,ncbi_57571,ncbi_80008
hsa-miR-4798-5p	3	ncbi_646851,ncbi_55244,ncbi_130733
hsa-miR-484	56	ncbi_24,ncbi_7066,ncbi_1435,ncbi_768239,ncbi_2786,ncbi_285966,ncbi_6932,ncbi_6518,ncbi_4773,ncbi_149478,ncbi_4739,ncbi_9542,ncbi_7185,ncbi_5137,ncbi_8942,ncbi_5798,ncbi_9033,ncbi_3856,ncbi_57101,ncbi_9415,ncbi_2044,ncbi_124590,ncbi_11148,ncbi_4261,ncbi_83690,ncbi_8076,ncbi_27071,ncbi_150372,ncbi_84941,ncbi_3604,ncbi_4600,ncbi_4939,ncbi_5029,ncbi_5579,ncbi_9037,ncbi_1463,ncbi_10893,ncbi_9915,ncbi_26049,ncbi_51435,ncbi_8862,ncbi_55512,ncbi_56241,ncbi_57462,ncbi_64284,ncbi_153478,ncbi_127707,ncbi_147138,ncbi_64856,ncbi_80737,ncbi_136306,ncbi_84632,ncbi_23452,ncbi_285489,ncbi_343990,ncbi_158584
hsa-miR-519a-3p	32	ncbi_5896,ncbi_4987,ncbi_23779,ncbi_9901,ncbi_23213,ncbi_29767,ncbi_6565,ncbi_2018,ncbi_2244,ncbi_84679,ncbi_100506127,ncbi_147463,ncbi_57578,ncbi_84449,ncbi_100129924,ncbi_340351,ncbi_2634,ncbi_9095,ncbi_56605,ncbi_9724,ncbi_60529,ncbi_136647,ncbi_130888,ncbi_553158,ncbi_79846,ncbi_285755,ncbi_102724488,ncbi_339541,ncbi_130733,ncbi_134526,ncbi_266722,ncbi_107987276
hsa-miR-521	2	ncbi_57834,ncbi_266743
hsa-miR-548f-3p	13	ncbi_785,ncbi_4987,ncbi_9331,ncbi_7700,ncbi_84449,ncbi_102724488,ncbi_2124,ncbi_9658,ncbi_9050,ncbi_84253,ncbi_91749,ncbi_388394,ncbi_56605
hsa-miR-556-3p	16	ncbi_5675,ncbi_8626,ncbi_5673,ncbi_4776,ncbi_5137,ncbi_145447,ncbi_8835,ncbi_2044,ncbi_27071,ncbi_2247,ncbi_5579,ncbi_9037,ncbi_81706,ncbi_117157,ncbi_85236,ncbi_8942
hsa-miR-627-3p	66	ncbi_1281,ncbi_2155,ncbi_5054,ncbi_3479,ncbi_1734,ncbi_5143,ncbi_339184,ncbi_284434,ncbi_145781,ncbi_6263,ncbi_5142,ncbi_4222,ncbi_26471,ncbi_50940,ncbi_120939,ncbi_8626,ncbi_171024,ncbi_285966,ncbi_147409,ncbi_131578,ncbi_4773,ncbi_81493,ncbi_4790,ncbi_8942,ncbi_1520,ncbi_26298,ncbi_64699,ncbi_57535,ncbi_999,ncbi_128414,ncbi_166336,ncbi_3604,ncbi_1634,ncbi_3371,ncbi_3641,ncbi_3860,ncbi_9037,ncbi_2984,ncbi_3037,ncbi_10148,ncbi_9770,ncbi_9915,ncbi_26040,ncbi_51351,

MiRNA	Target number	Target gene
		ncbi_55107,ncbi_10129,ncbi_79413,ncbi_79442,ncbi_90993,ncbi_85236,ncbi_91947,ncbi_171483,ncbi_5029,ncbi_56606,ncbi_5239,ncbi_55084,ncbi_80201,ncbi_10628,ncbi_1141,ncbi_343990,ncbi_55577,ncbi_8835,ncbi_1800,ncbi_105371921,ncbi_5105,ncbi_80008
hsa-miR-628-3p	21	ncbi_94,ncbi_1734,ncbi_440603,ncbi_645121,ncbi_50940,ncbi_88,ncbi_6932,ncbi_1780,ncbi_81493,ncbi_4015,ncbi_6512,ncbi_79788,ncbi_1634,ncbi_5029,ncbi_5579,ncbi_27076,ncbi_30811,ncbi_10129,ncbi_79628,ncbi_117157,ncbi_254228
hsa-miR-6511a-3p	42	ncbi_3689,ncbi_5317,ncbi_1435,ncbi_342897,ncbi_84632,ncbi_90427,ncbi_8821,ncbi_149461,ncbi_4773,ncbi_4776,ncbi_2521,ncbi_8942,ncbi_534,ncbi_26298,ncbi_10752,ncbi_5570,ncbi_56936,ncbi_389602,ncbi_140731,ncbi_163782,ncbi_50632,ncbi_6518,ncbi_58476,ncbi_1735,ncbi_285966,ncbi_5105,ncbi_3005,ncbi_3037,ncbi_3702,ncbi_7805,ncbi_1734,ncbi_30811,ncbi_9915,ncbi_26049,ncbi_51351,ncbi_55512,ncbi_79628,ncbi_147138,ncbi_348013,ncbi_4739,ncbi_147409,ncbi_9033
hsa-miR-6516-5p	25	ncbi_4143,ncbi_785,ncbi_2824,ncbi_6752,ncbi_7296,ncbi_285268,ncbi_55186,ncbi_285755,ncbi_196394,ncbi_4753,ncbi_5346,ncbi_7358,ncbi_84071,ncbi_84253,ncbi_388325,ncbi_7700,ncbi_283726,ncbi_140564,ncbi_340351,ncbi_102724488,ncbi_56648,ncbi_79690,ncbi_80032,ncbi_91749,ncbi_23418
hsa-miR-6732-3p	54	ncbi_94,ncbi_2099,ncbi_6469,ncbi_4261,ncbi_4359,ncbi_3479,ncbi_3589,ncbi_10659,ncbi_26471,ncbi_50940,ncbi_1261,ncbi_171024,ncbi_285966,ncbi_6518,ncbi_4776,ncbi_9214,ncbi_63901,ncbi_440689,ncbi_2521,ncbi_5137,ncbi_1520,ncbi_64699,ncbi_57535,ncbi_53615,ncbi_376267,ncbi_163782,ncbi_9507,ncbi_80345,ncbi_2247,ncbi_166336,ncbi_3604,ncbi_1805,ncbi_2570,ncbi_5029,ncbi_1463,ncbi_6578,ncbi_11240,ncbi_30811,ncbi_23105,ncbi_8862,ncbi_55742,ncbi_55512,ncbi_10060,ncbi_114897,ncbi_153478,ncbi_4739,ncbi_4135,ncbi_80737,ncbi_56606,ncbi_102724398,ncbi_653145,ncbi_57571,ncbi_343990,ncbi_284021
hsa-miR-6741-3p	19	ncbi_1435,ncbi_6536,ncbi_54921,ncbi_768239,ncbi_2825,ncbi_131578,ncbi_285489,ncbi_63924,ncbi_101928841,ncbi_8355,ncbi_9770,ncbi_8862,ncbi_54625,ncbi_64856,ncbi_140893,ncbi_147138,ncbi_161753,ncbi_79173,ncbi_6578
hsa-miR-676-3p	13	ncbi_29767,ncbi_81491,ncbi_84679,ncbi_100506127,ncbi_1645,ncbi_5017,ncbi_57161,ncbi_9050,ncbi_136647,ncbi_285349,ncbi_130733,ncbi_266722,ncbi_374739
hsa-miR-6786-3p	10	ncbi_1435,ncbi_26471,ncbi_401934,ncbi_389602,ncbi_50632,ncbi_55084,ncbi_64856,ncbi_102724398,ncbi_2099,ncbi_153478
hsa-miR-6837-5p	26	ncbi_4987,ncbi_27040,ncbi_9901,ncbi_84439,ncbi_57834,ncbi_100526664,ncbi_939,ncbi_113451,ncbi_23349,ncbi_100506127,ncbi_1950,ncbi_140564,ncbi_8635,ncbi_5017,ncbi_4323,ncbi_11076,ncbi_9717,ncbi_23302,ncbi_128434,ncbi_10841,ncbi_440829,ncbi_196500,ncbi_84700,ncbi_90249,ncbi_785,ncbi_112268350
hsa-miR-7-5p	23	ncbi_785,ncbi_152098,ncbi_339210,ncbi_285268,ncbi_285755,ncbi_84439,ncbi_1638,ncbi_119,ncbi_283710,ncbi_100131539,ncbi_100506127,ncbi_9331,ncbi_7700,ncbi_6004,ncbi_10004,ncbi_84249,ncbi_136647,ncbi_138065,ncbi_91749,ncbi_220108,ncbi_1768,ncbi_266722,ncbi_374739
hsa-miR-7704	13	ncbi_1589,ncbi_6536,ncbi_149461,ncbi_321,ncbi_9542,ncbi_9415,ncbi_50632,ncbi_5579,ncbi_8740,ncbi_2810,ncbi_55742,ncbi_64856,ncbi_127294
hsa-miR-7854-3p	22	ncbi_3561,ncbi_1435,ncbi_1734,ncbi_10659,ncbi_3172,ncbi_7161,ncbi_643382,ncbi_2494,ncbi_83882,ncbi_376267,ncbi_5271,ncbi_138050,ncbi_8633,ncbi_10107,ncbi_79628,ncbi_79817,ncbi_84443,ncbi_254228,ncbi_80274,ncbi_4739,ncbi_3134,ncbi_343990
hsa-miR-7974	14	ncbi_54039,ncbi_5346,ncbi_1101,ncbi_333926,ncbi_25956,ncbi_57161,ncbi_23213,ncbi_23302,ncbi_80852,ncbi_57578,ncbi_10004,ncbi_266722,ncbi_23415,ncbi_92270
hsa-miR-7976	65	ncbi_286,ncbi_2099,ncbi_5745,ncbi_733,ncbi_3479,ncbi_90427,ncbi_9938,ncbi_27293,ncbi_4582,ncbi_6241,ncbi_643866,ncbi_26471,ncbi_79098,ncbi_79919,ncbi_116535,ncbi_8821,ncbi_7161,ncbi_171024,ncbi_131578,ncbi_5328,ncbi_397,ncbi_100134444,ncbi_30851,ncbi_57535,ncbi_54587,ncbi_389602,ncbi_285489,ncbi_58476,ncbi_8456,ncbi_3316,ncbi_3625,ncbi_3866,ncbi_5029,ncbi_8365,ncbi_9507,ncbi_7471,ncbi_23581,ncbi_9770,ncbi_23105,ncbi_51351,ncbi_51702,ncbi_51703,ncbi_54625,ncbi_55512

MiRNA	Target number	Target gene
		,ncbi_56103,ncbi_56099,ncbi_8969,ncbi_79413,ncbi_79628,ncbi_153478,ncbi_127707,ncbi_80274,ncbi_348013,ncbi_4135,ncbi_55106,ncbi_102724398,ncbi_5239,ncbi_342897,ncbi_105373347,ncbi_7433,ncbi_1141,ncbi_64284,ncbi_56606,ncbi_105369535,ncbi_158584
hsa-miR-877-5p	12	ncbi_440603,ncbi_171024,ncbi_4015,ncbi_8942,ncbi_5271,ncbi_124590,ncbi_140711,ncbi_26040,ncbi_26049,ncbi_10107,ncbi_57462,ncbi_57571
hsa-miR-890	19	ncbi_286223,ncbi_401265,ncbi_7296,ncbi_285268,ncbi_55186,ncbi_2244,ncbi_100506127,ncbi_7700,ncbi_1645,ncbi_107984345,ncbi_6004,ncbi_2124,ncbi_11076,ncbi_23302,ncbi_119,ncbi_57161,ncbi_10219,ncbi_266722,ncbi_107987276
hsa-miR-93-3p	31	ncbi_1435,ncbi_653145,ncbi_8710,ncbi_120939,ncbi_728113,ncbi_51062,ncbi_171024,ncbi_285966,ncbi_26298,ncbi_834,ncbi_9415,ncbi_283229,ncbi_6518,ncbi_138050,ncbi_1212,ncbi_445577,ncbi_166336,ncbi_1634,ncbi_1463,ncbi_10107,ncbi_23371,ncbi_57462,ncbi_80274,ncbi_348013,ncbi_136306,ncbi_7433,ncbi_55084,ncbi_1141,ncbi_25903,ncbi_105369535,ncbi_158584
hsa-miR-935	11	ncbi_3560,ncbi_440603,ncbi_169270,ncbi_8626,ncbi_5570,ncbi_1735,ncbi_26040,ncbi_10060,ncbi_79628,ncbi_4261,ncbi_80008
miR-10240-x	30	ncbi_2200,ncbi_3486,ncbi_145781,ncbi_54437,ncbi_645121,ncbi_171024,ncbi_131578,ncbi_4773,ncbi_5137,ncbi_534,ncbi_7433,ncbi_8821,ncbi_3576,ncbi_2570,ncbi_2920,ncbi_9037,ncbi_9283,ncbi_3702,ncbi_6578,ncbi_7429,ncbi_23452,ncbi_55512,ncbi_84443,ncbi_91947,ncbi_56606,ncbi_25903,ncbi_3134,ncbi_3479,ncbi_113220,ncbi_57188
miR-10285-x	32	ncbi_1435,ncbi_339184,ncbi_145781,ncbi_54437,ncbi_8942,ncbi_26471,ncbi_50940,ncbi_5655,ncbi_2786,ncbi_6518,ncbi_11309,ncbi_79674,ncbi_10752,ncbi_53615,ncbi_58476,ncbi_716,ncbi_8456,ncbi_1958,ncbi_5029,ncbi_6367,ncbi_7047,ncbi_3005,ncbi_7429,ncbi_30811,ncbi_55561,ncbi_79628,ncbi_7473,ncbi_84814,ncbi_9134,ncbi_348013,ncbi_4773,ncbi_343990
miR-10401-y	11	ncbi_50940,ncbi_6932,ncbi_647024,ncbi_401934,ncbi_57535,ncbi_56936,ncbi_55561,ncbi_79442,ncbi_158248,ncbi_105371921,ncbi_79895
miR-10555-x	35	ncbi_554,ncbi_90427,ncbi_164395,ncbi_220979,ncbi_140453,ncbi_26471,ncbi_56649,ncbi_8626,ncbi_55733,ncbi_4776,ncbi_91156,ncbi_7185,ncbi_57535,ncbi_376267,ncbi_58476,ncbi_79173,ncbi_4148,ncbi_5579,ncbi_5744,ncbi_7462,ncbi_30811,ncbi_51703,ncbi_55107,ncbi_79895,ncbi_81606,ncbi_251,ncbi_113220,ncbi_4135,ncbi_4773,ncbi_2099,ncbi_163782,ncbi_8942,ncbi_158584,ncbi_284021,ncbi_56606
miR-10955-y	3	ncbi_5054,ncbi_221527,ncbi_107984590
miR-11202-x	13	ncbi_1589,ncbi_653145,ncbi_768239,ncbi_728113,ncbi_26287,ncbi_6932,ncbi_5540,ncbi_9283,ncbi_286204,ncbi_100996758,ncbi_105373347,ncbi_59350,ncbi_51435
miR-1180-y	4	ncbi_5724,ncbi_90427,ncbi_1634,ncbi_80274
miR-11975-x	11	ncbi_2902,ncbi_6536,ncbi_8542,ncbi_53841,ncbi_221468,ncbi_83882,ncbi_3014,ncbi_23237,ncbi_64856,ncbi_102724398,ncbi_55765
miR-11976-y	11	ncbi_2902,ncbi_6536,ncbi_8542,ncbi_53841,ncbi_221468,ncbi_83882,ncbi_3014,ncbi_23237,ncbi_64856,ncbi_102724398,ncbi_55765
miR-1249-y	6	ncbi_3486,ncbi_64699,ncbi_5029,ncbi_9379,ncbi_64856,ncbi_145501
miR-126-y	1	ncbi_55765
miR-1260-x	17	ncbi_1734,ncbi_51151,ncbi_5047,ncbi_8626,ncbi_1520,ncbi_389602,ncbi_150372,ncbi_30811,ncbi_9770,ncbi_79442,ncbi_79628,ncbi_117157,ncbi_286204,ncbi_100996758,ncbi_57462,ncbi_7433,ncbi_5540
miR-127-y	3	ncbi_55742,ncbi_147138,ncbi_8835
miR-1275-x	76	ncbi_286,ncbi_2099,ncbi_2155,ncbi_2200,ncbi_1439,ncbi_3172,ncbi_6556,ncbi_5054,ncbi_3479,ncbi_7448,ncbi_1141,ncbi_90427,ncbi_2707,ncbi_440603,ncbi_10659,ncbi_4939,ncbi_220979,ncbi_643866,ncbi_8710,ncbi_26471,ncbi_116535,ncbi_7161,ncbi_6932,ncbi_9214,ncbi_55765,ncbi_5328,ncbi_221468,ncbi_285489,ncbi_79674,ncbi_53841,ncbi_401934,ncbi_9415,ncbi_11148,ncbi_54587,ncbi_171024,ncbi_1436,ncbi_8764,ncbi_376267,ncbi_50632,ncbi_6518,ncbi_342667,ncbi_5159,ncbi_128414,ncbi_112694756,ncbi_8456,ncbi_166336,ncbi_3604,ncbi_2901,ncbi_6367,ncbi_1463,ncbi_9283,

MiRNA	Target number	Target gene
		ncbi_4584,ncbi_10893,ncbi_11131,ncbi_30811,ncbi_51703,ncbi_8862,ncbi_55742,ncbi_56103,ncbi_56099,ncbi_10129,ncbi_114897,ncbi_84443,ncbi_90226,ncbi_80274,ncbi_348013,ncbi_4739,ncbi_5744,ncbi_286204,ncbi_55106,ncbi_5029,ncbi_339184,ncbi_4222,ncbi_9542,ncbi_9033,ncbi_25903
miR-1283-x	29	ncbi_286223,ncbi_492311,ncbi_283726,ncbi_118490,ncbi_55186,ncbi_23213,ncbi_29767,ncbi_81491,ncbi_113451,ncbi_221336,ncbi_266743,ncbi_4987,ncbi_57578,ncbi_57718,ncbi_110116772,ncbi_100129924,ncbi_2634,ncbi_9095,ncbi_25797,ncbi_57161,ncbi_136647,ncbi_138065,ncbi_339488,ncbi_90249,ncbi_84071,ncbi_10219,ncbi_266722,ncbi_107987276,ncbi_54039
miR-1304-y	18	ncbi_6531,ncbi_768239,ncbi_8626,ncbi_285489,ncbi_81888,ncbi_3773,ncbi_53615,ncbi_4261,ncbi_80345,ncbi_3604,ncbi_6585,ncbi_11131,ncbi_56241,ncbi_79442,ncbi_153478,ncbi_140893,ncbi_2323,ncbi_102724398
miR-1343-y	74	ncbi_286,ncbi_554,ncbi_2263,ncbi_4261,ncbi_7051,ncbi_2006,ncbi_6556,ncbi_624,ncbi_164395,ncbi_4939,ncbi_6531,ncbi_5655,ncbi_79098,ncbi_124976,ncbi_7161,ncbi_171024,ncbi_321,ncbi_131578,ncbi_11309,ncbi_643382,ncbi_647024,ncbi_56834,ncbi_8479,ncbi_146547,ncbi_56936,ncbi_84699,ncbi_51421,ncbi_53615,ncbi_2044,ncbi_4815,ncbi_285489,ncbi_140711,ncbi_27071,ncbi_2099,ncbi_84941,ncbi_3604,ncbi_1298,ncbi_3371,ncbi_6585,ncbi_7134,ncbi_4606,ncbi_4868,ncbi_3702,ncbi_3108,ncbi_10893,ncbi_11131,ncbi_11240,ncbi_25759,ncbi_9915,ncbi_23237,ncbi_56103,ncbi_56099,ncbi_64856,ncbi_10129,ncbi_84443,ncbi_113220,ncbi_126868,ncbi_80274,ncbi_128209,ncbi_51435,ncbi_2323,ncbi_100996758,ncbi_153478,ncbi_2902,ncbi_2901,ncbi_2570,ncbi_9542,ncbi_5540,ncbi_6932,ncbi_105369535,ncbi_3479,ncbi_55106,ncbi_8456,ncbi_147138
miR-1346-y	8	ncbi_100506115,ncbi_6512,ncbi_27293,ncbi_60676,ncbi_153478,ncbi_286204,ncbi_2902,ncbi_3965
miR-1386-x	46	ncbi_286,ncbi_3576,ncbi_3479,ncbi_2914,ncbi_440603,ncbi_220979,ncbi_116372,ncbi_8626,ncbi_321,ncbi_5349,ncbi_8542,ncbi_4739,ncbi_387787,ncbi_91156,ncbi_9542,ncbi_8479,ncbi_2494,ncbi_83690,ncbi_6512,ncbi_285489,ncbi_140731,ncbi_101928841,ncbi_27071,ncbi_3575,ncbi_8633,ncbi_4868,ncbi_9507,ncbi_10991,ncbi_25759,ncbi_27254,ncbi_9770,ncbi_23237,ncbi_26040,ncbi_55084,ncbi_55107,ncbi_79817,ncbi_127707,ncbi_221527,ncbi_375791,ncbi_64856,ncbi_56606,ncbi_11148,ncbi_57462,ncbi_126868,ncbi_57101,ncbi_158584
miR-139-x	26	ncbi_286,ncbi_1141,ncbi_84632,ncbi_339184,ncbi_8821,ncbi_8626,ncbi_149461,ncbi_643382,ncbi_6866,ncbi_2494,ncbi_79788,ncbi_2247,ncbi_3575,ncbi_3725,ncbi_2662,ncbi_7471,ncbi_57530,ncbi_10129,ncbi_79628,ncbi_84443,ncbi_6925,ncbi_4773,ncbi_343990,ncbi_729857,ncbi_153478,ncbi_5672
miR-143-y	37	ncbi_3172,ncbi_3486,ncbi_5054,ncbi_2562,ncbi_2786,ncbi_88,ncbi_55733,ncbi_285966,ncbi_6932,ncbi_4773,ncbi_63901,ncbi_563,ncbi_8942,ncbi_401934,ncbi_5672,ncbi_376267,ncbi_999,ncbi_9507,ncbi_5271,ncbi_1298,ncbi_1634,ncbi_1958,ncbi_9037,ncbi_6578,ncbi_10107,ncbi_56241,ncbi_10060,ncbi_57462,ncbi_57530,ncbi_81606,ncbi_136306,ncbi_100996758,ncbi_339184,ncbi_5540,ncbi_157855,ncbi_3134,ncbi_105369535
miR-146-x	47	ncbi_94,ncbi_3914,ncbi_6556,ncbi_1734,ncbi_5724,ncbi_10659,ncbi_116372,ncbi_79919,ncbi_4773,ncbi_4778,ncbi_55765,ncbi_81493,ncbi_26298,ncbi_3773,ncbi_2044,ncbi_4261,ncbi_6512,ncbi_112399,ncbi_999,ncbi_150372,ncbi_2635,ncbi_2099,ncbi_58476,ncbi_55577,ncbi_5271,ncbi_2247,ncbi_284021,ncbi_166336,ncbi_1634,ncbi_5579,ncbi_5880,ncbi_8633,ncbi_9507,ncbi_11240,ncbi_23452,ncbi_9770,ncbi_57462,ncbi_117157,ncbi_254228,ncbi_145501,ncbi_55106,ncbi_84632,ncbi_157855,ncbi_343990,ncbi_3479,ncbi_105371921,ncbi_80008
miR-1599-y	54	ncbi_286,ncbi_345,ncbi_2200,ncbi_1589,ncbi_4359,ncbi_1141,ncbi_343521,ncbi_145781,ncbi_768239,ncbi_88,ncbi_149461,ncbi_285489,ncbi_57571,ncbi_3856,ncbi_84699,ncbi_53615,ncbi_54587,ncbi_4261,ncbi_395,ncbi_1436,ncbi_102800317,ncbi_140731,ncbi_27293,ncbi_2170,ncbi_58476,ncbi_55124,ncbi_105375355,ncbi_5159,ncbi_445577,ncbi_1464,ncbi_4939,ncbi_5744,ncbi_6367,ncbi_8334,ncbi_8740,ncbi_9283,ncbi_

MiRNA	Target number	Target gene
		9507,ncbi_3702,ncbi_10991,ncbi_23237,ncbi_23371,ncbi_51351,ncbi_56649,ncbi_57462,ncbi_64856,ncbi_90993,ncbi_153478,ncbi_147138,ncbi_80274,ncbi_91947,ncbi_100996758,ncbi_4135,ncbi_2902,ncbi_5540
miR-193-y	51	ncbi_94,ncbi_1734,ncbi_10659,ncbi_54921,ncbi_120939,ncbi_6925,ncbi_55733,ncbi_4776,ncbi_4739,ncbi_5328,ncbi_285489,ncbi_7185,ncbi_5137,ncbi_8479,ncbi_729220,ncbi_8835,ncbi_2044,ncbi_256158,ncbi_57188,ncbi_340061,ncbi_105375355,ncbi_5271,ncbi_166336,ncbi_2920,ncbi_4939,ncbi_5029,ncbi_6585,ncbi_9037,ncbi_6578,ncbi_10893,ncbi_30811,ncbi_60676,ncbi_79442,ncbi_79628,ncbi_84443,ncbi_84962,ncbi_89870,ncbi_153478,ncbi_79987,ncbi_80274,ncbi_286204,ncbi_3017,ncbi_8764,ncbi_23452,ncbi_221468,ncbi_653145,ncbi_25903,ncbi_51435,ncbi_3479,ncbi_284021,ncbi_80008
miR-194-x	19	ncbi_388650,ncbi_151556,ncbi_285755,ncbi_23213,ncbi_81491,ncbi_100526664,ncbi_25850,ncbi_619189,ncbi_144321,ncbi_9745,ncbi_340351,ncbi_107984345,ncbi_9658,ncbi_25956,ncbi_57161,ncbi_80032,ncbi_136647,ncbi_91749,ncbi_84700
miR-195-x	34	ncbi_135,ncbi_785,ncbi_4987,ncbi_646851,ncbi_6752,ncbi_80852,ncbi_81491,ncbi_119,ncbi_220108,ncbi_100131539,ncbi_23349,ncbi_100506127,ncbi_147463,ncbi_56704,ncbi_102724488,ncbi_2979,ncbi_726,ncbi_23415,ncbi_9717,ncbi_9745,ncbi_23302,ncbi_55586,ncbi_56648,ncbi_60529,ncbi_285349,ncbi_339488,ncbi_57161,ncbi_10841,ncbi_84700,ncbi_90249,ncbi_100289279,ncbi_1950,ncbi_283710,ncbi_54039
miR-197-y	47	ncbi_2200,ncbi_3484,ncbi_3486,ncbi_5724,ncbi_342897,ncbi_84632,ncbi_90427,ncbi_284434,ncbi_6241,ncbi_5655,ncbi_768239,ncbi_8626,ncbi_6518,ncbi_5349,ncbi_4773,ncbi_1015,ncbi_4599,ncbi_647024,ncbi_26298,ncbi_10752,ncbi_124590,ncbi_4261,ncbi_999,ncbi_150372,ncbi_58476,ncbi_716,ncbi_138050,ncbi_1212,ncbi_3641,ncbi_5029,ncbi_3005,ncbi_30811,ncbi_23105,ncbi_26040,ncbi_55561,ncbi_64284,ncbi_84443,ncbi_84517,ncbi_153478,ncbi_338382,ncbi_53841,ncbi_339184,ncbi_9033,ncbi_107984590,ncbi_147138,ncbi_8740,ncbi_158584
miR-2116-y	42	ncbi_5317,ncbi_7066,ncbi_5054,ncbi_388595,ncbi_4939,ncbi_645121,ncbi_169270,ncbi_55733,ncbi_141,ncbi_91156,ncbi_285489,ncbi_7433,ncbi_8835,ncbi_2494,ncbi_9415,ncbi_389602,ncbi_140731,ncbi_10628,ncbi_163782,ncbi_6518,ncbi_2247,ncbi_1735,ncbi_138050,ncbi_445577,ncbi_3575,ncbi_5579,ncbi_6274,ncbi_5971,ncbi_27289,ncbi_9770,ncbi_51435,ncbi_8862,ncbi_10060,ncbi_79442,ncbi_3371,ncbi_55106,ncbi_23452,ncbi_256158,ncbi_9542,ncbi_653145,ncbi_3479,ncbi_57101
miR-2137-y	5	ncbi_84439,ncbi_5346,ncbi_728392,ncbi_939,ncbi_11076
miR-223-x	29	ncbi_1281,ncbi_2200,ncbi_24,ncbi_1141,ncbi_164395,ncbi_10659,ncbi_6263,ncbi_50940,ncbi_79098,ncbi_8626,ncbi_81493,ncbi_338382,ncbi_8942,ncbi_59350,ncbi_83690,ncbi_79788,ncbi_2247,ncbi_8740,ncbi_9507,ncbi_55742,ncbi_79442,ncbi_79983,ncbi_80274,ncbi_91947,ncbi_10274,ncbi_166336,ncbi_51435,ncbi_3479,ncbi_57188
miR-223-y	21	ncbi_24,ncbi_1141,ncbi_1734,ncbi_342897,ncbi_90427,ncbi_3172,ncbi_4222,ncbi_6925,ncbi_285966,ncbi_4773,ncbi_2562,ncbi_729220,ncbi_2247,ncbi_166336,ncbi_5579,ncbi_7805,ncbi_5239,ncbi_84962,ncbi_127707,ncbi_30811,ncbi_343990
miR-235-y	17	ncbi_2200,ncbi_624,ncbi_8942,ncbi_171024,ncbi_9214,ncbi_7185,ncbi_57188,ncbi_999,ncbi_10659,ncbi_158584,ncbi_2247,ncbi_5971,ncbi_10129,ncbi_84443,ncbi_91947,ncbi_91752,ncbi_729857
miR-2404-x	23	ncbi_2155,ncbi_2786,ncbi_8626,ncbi_9058,ncbi_4057,ncbi_10752,ncbi_57535,ncbi_4261,ncbi_57188,ncbi_55577,ncbi_348013,ncbi_1212,ncbi_5579,ncbi_7837,ncbi_79628,ncbi_114769,ncbi_3575,ncbi_338382,ncbi_23452,ncbi_2901,ncbi_343990,ncbi_8942,ncbi_3134
miR-2478-y	54	ncbi_7066,ncbi_6556,ncbi_624,ncbi_1734,ncbi_2563,ncbi_3352,ncbi_164395,ncbi_6536,ncbi_3172,ncbi_645121,ncbi_26471,ncbi_50940,ncbi_79098,ncbi_2786,ncbi_171024,ncbi_131578,ncbi_11309,ncbi_316,ncbi_4015,ncbi_8942,ncbi_8835,ncbi_51421,ncbi_4261,ncbi_6512,ncbi_55106,ncbi_256158,ncbi_140711,ncbi_50632,ncbi_6518,ncbi_105375355,ncbi_27129,ncbi_166336,ncbi_1464,ncbi_3725,ncbi_5579,ncbi_8740,ncbi_1463,ncbi_9507,ncbi_8843,ncbi_10107,ncbi_51286,ncbi_57462,ncbi_79628,ncbi_81606

MiRNA	Target number	Target gene
		,ncbi_81706,ncbi_153478,ncbi_9134,ncbi_127294,ncbi_80274,ncbi_221527,ncbi_348013,ncbi_81888,ncbi_90993,ncbi_5239
miR-2779-y	26	ncbi_6556,ncbi_624,ncbi_5345,ncbi_54921,ncbi_645121,ncbi_321,ncbi_131578,ncbi_149478,ncbi_4790,ncbi_53841,ncbi_768,ncbi_376267,ncbi_3860,ncbi_9283,ncbi_7837,ncbi_26040,ncbi_8862,ncbi_54625,ncbi_57530,ncbi_64856,ncbi_7473,ncbi_161753,ncbi_102724398,ncbi_25903,ncbi_146547,ncbi_6578
miR-2779-z	8	ncbi_1435,ncbi_5724,ncbi_4261,ncbi_150372,ncbi_1634,ncbi_9915,ncbi_79413,ncbi_105373347
miR-301-y	25	ncbi_5896,ncbi_646851,ncbi_6752,ncbi_23213,ncbi_29767,ncbi_119,ncbi_100526664,ncbi_196500,ncbi_126549,ncbi_84449,ncbi_100506127,ncbi_7700,ncbi_102724488,ncbi_9095,ncbi_9658,ncbi_9717,ncbi_56648,ncbi_60529,ncbi_91749,ncbi_79846,ncbi_785,ncbi_55840,ncbi_266722,ncbi_56605,ncbi_112268350
miR-31-y	4	ncbi_785,ncbi_23213,ncbi_23349,ncbi_57161
miR-310-y	16	ncbi_2200,ncbi_624,ncbi_8942,ncbi_171024,ncbi_8542,ncbi_9214,ncbi_57188,ncbi_999,ncbi_10659,ncbi_158584,ncbi_2247,ncbi_5971,ncbi_84443,ncbi_91947,ncbi_91752,ncbi_729857
miR-3123-y	43	ncbi_2099,ncbi_6469,ncbi_6556,ncbi_9938,ncbi_6536,ncbi_6241,ncbi_116372,ncbi_5655,ncbi_79919,ncbi_3479,ncbi_171024,ncbi_26040,ncbi_4773,ncbi_397,ncbi_8942,ncbi_26298,ncbi_64699,ncbi_51421,ncbi_55106,ncbi_8076,ncbi_2247,ncbi_9283,ncbi_9507,ncbi_9568,ncbi_11240,ncbi_30811,ncbi_51435,ncbi_55084,ncbi_55742,ncbi_56649,ncbi_60676,ncbi_3885,ncbi_10129,ncbi_79442,ncbi_79817,ncbi_80201,ncbi_7473,ncbi_254228,ncbi_158248,ncbi_5239,ncbi_59350,ncbi_25903,ncbi_343990
miR-3186-y	3	ncbi_4987,ncbi_11076,ncbi_9658
miR-3187-y	24	ncbi_6469,ncbi_624,ncbi_3352,ncbi_4222,ncbi_120939,ncbi_321,ncbi_338382,ncbi_79788,ncbi_166336,ncbi_1307,ncbi_1958,ncbi_2920,ncbi_5029,ncbi_9507,ncbi_6578,ncbi_10107,ncbi_25759,ncbi_27289,ncbi_55742,ncbi_114897,ncbi_171483,ncbi_57188,ncbi_55084,ncbi_25903
miR-338-y	37	ncbi_5896,ncbi_785,ncbi_286223,ncbi_388650,ncbi_646851,ncbi_118490,ncbi_9901,ncbi_84439,ncbi_2244,ncbi_119,ncbi_27111,ncbi_220108,ncbi_257019,ncbi_90527,ncbi_100506127,ncbi_113451,ncbi_65266,ncbi_10219,ncbi_164592,ncbi_84142,ncbi_7700,ncbi_9745,ncbi_6004,ncbi_8635,ncbi_2634,ncbi_1844,ncbi_9717,ncbi_53905,ncbi_56648,ncbi_57161,ncbi_80032,ncbi_128434,ncbi_136647,ncbi_285349,ncbi_80852,ncbi_134526,ncbi_1645
miR-340-x	22	ncbi_388650,ncbi_646851,ncbi_6752,ncbi_80852,ncbi_81491,ncbi_6565,ncbi_7358,ncbi_84561,ncbi_25850,ncbi_84679,ncbi_3790,ncbi_9331,ncbi_340351,ncbi_100129924,ncbi_9724,ncbi_136647,ncbi_143282,ncbi_285349,ncbi_84700,ncbi_55840,ncbi_84071,ncbi_266722
miR-3934-x	18	ncbi_5896,ncbi_2824,ncbi_283726,ncbi_118490,ncbi_6517,ncbi_119,ncbi_144321,ncbi_100506127,ncbi_388325,ncbi_84439,ncbi_7700,ncbi_23302,ncbi_9724,ncbi_9050,ncbi_136647,ncbi_92270,ncbi_1645,ncbi_107984859
miR-3940-y	22	ncbi_2155,ncbi_1141,ncbi_3172,ncbi_768239,ncbi_2786,ncbi_397,ncbi_146547,ncbi_50632,ncbi_84941,ncbi_100129484,ncbi_9507,ncbi_3005,ncbi_9568,ncbi_8971,ncbi_23452,ncbi_55561,ncbi_153478,ncbi_127707,ncbi_80274,ncbi_64856,ncbi_25759,ncbi_653145
miR-3960-y	8	ncbi_2902,ncbi_8542,ncbi_53841,ncbi_1436,ncbi_83882,ncbi_23237,ncbi_64856,ncbi_55765
miR-3963-x	41	ncbi_7066,ncbi_6556,ncbi_624,ncbi_1734,ncbi_3352,ncbi_164395,ncbi_6536,ncbi_645121,ncbi_50940,ncbi_79098,ncbi_2786,ncbi_171024,ncbi_131578,ncbi_316,ncbi_8942,ncbi_8835,ncbi_6512,ncbi_55106,ncbi_256158,ncbi_140711,ncbi_50632,ncbi_105375355,ncbi_27129,ncbi_166336,ncbi_3725,ncbi_5579,ncbi_1463,ncbi_9507,ncbi_8843,ncbi_10107,ncbi_57462,ncbi_79628,ncbi_81706,ncbi_153478,ncbi_9134,ncbi_127294,ncbi_80274,ncbi_221527,ncbi_81888,ncbi_90993,ncbi_5239
miR-3968-y	54	ncbi_7066,ncbi_6556,ncbi_624,ncbi_1734,ncbi_2563,ncbi_3352,ncbi_164395,ncbi_6536,ncbi_3172,ncbi_645121,ncbi_26471,ncbi_50940,ncbi_79098,ncbi_2786,ncbi_17102

MiRNA	Target number	Target gene
		4,ncbi_131578,ncbi_11309,ncbi_316,ncbi_4015,ncbi_8942,ncbi_8835,ncbi_51421,ncbi_4261,ncbi_6512,ncbi_55106,ncbi_256158,ncbi_140711,ncbi_50632,ncbi_6518,ncbi_105375355,ncbi_27129,ncbi_166336,ncbi_1464,ncbi_3725,ncbi_5579,ncbi_8740,ncbi_1463,ncbi_9507,ncbi_8843,ncbi_10107,ncbi_51286,ncbi_57462,ncbi_79628,ncbi_81606,ncbi_81706,ncbi_153478,ncbi_9134,ncbi_127294,ncbi_80274,ncbi_221527,ncbi_348013,ncbi_81888,ncbi_90993,ncbi_5239
miR-4286-y	41	ncbi_1439,ncbi_4359,ncbi_339761,ncbi_90427,ncbi_339184,ncbi_976,ncbi_55733,ncbi_2318,ncbi_171024,ncbi_647024,ncbi_79674,ncbi_8681,ncbi_57101,ncbi_53615,ncbi_4261,ncbi_4188,ncbi_140711,ncbi_376267,ncbi_1396,ncbi_3965,ncbi_27129,ncbi_342667,ncbi_3604,ncbi_3005,ncbi_10107,ncbi_27254,ncbi_30811,ncbi_9915,ncbi_8862,ncbi_55561,ncbi_57462,ncbi_64856,ncbi_84443,ncbi_84962,ncbi_3017,ncbi_221527,ncbi_128414,ncbi_653145,ncbi_1141,ncbi_64284,ncbi_105369535
miR-4286-z	58	ncbi_1439,ncbi_4359,ncbi_2902,ncbi_339761,ncbi_90427,ncbi_339184,ncbi_976,ncbi_54921,ncbi_55733,ncbi_149461,ncbi_2318,ncbi_131578,ncbi_4773,ncbi_5137,ncbi_8681,ncbi_8942,ncbi_2494,ncbi_57101,ncbi_53615,ncbi_4261,ncbi_140711,ncbi_376267,ncbi_1396,ncbi_150372,ncbi_50632,ncbi_3965,ncbi_348013,ncbi_27129,ncbi_342667,ncbi_3604,ncbi_6585,ncbi_8633,ncbi_4868,ncbi_9283,ncbi_9507,ncbi_3005,ncbi_10107,ncbi_11240,ncbi_27289,ncbi_30811,ncbi_9915,ncbi_23105,ncbi_8862,ncbi_55561,ncbi_57462,ncbi_64856,ncbi_79628,ncbi_84962,ncbi_80274,ncbi_3017,ncbi_221527,ncbi_128414,ncbi_653145,ncbi_80201,ncbi_1141,ncbi_64284,ncbi_105369535,ncbi_79895
miR-4425-y	11	ncbi_4143,ncbi_646851,ncbi_6517,ncbi_285268,ncbi_1907,ncbi_102724488,ncbi_9658,ncbi_57161,ncbi_285349,ncbi_90249,ncbi_100506127
miR-4443-x	35	ncbi_4359,ncbi_55733,ncbi_124976,ncbi_7161,ncbi_79674,ncbi_8942,ncbi_1520,ncbi_534,ncbi_729220,ncbi_53615,ncbi_376267,ncbi_79574,ncbi_58476,ncbi_27129,ncbi_5029,ncbi_6367,ncbi_4584,ncbi_7062,ncbi_7429,ncbi_23581,ncbi_26040,ncbi_55512,ncbi_56649,ncbi_60676,ncbi_5239,ncbi_79628,ncbi_127707,ncbi_80274,ncbi_56606,ncbi_23105,ncbi_25903,ncbi_55577,ncbi_153478,ncbi_3479,ncbi_81493
miR-4447-y	35	ncbi_275,ncbi_283726,ncbi_646851,ncbi_23779,ncbi_6517,ncbi_6752,ncbi_285268,ncbi_81491,ncbi_2018,ncbi_100526664,ncbi_643669,ncbi_196500,ncbi_100506127,ncbi_23107,ncbi_65266,ncbi_84439,ncbi_7700,ncbi_51626,ncbi_1645,ncbi_340351,ncbi_11076,ncbi_9717,ncbi_53905,ncbi_56648,ncbi_80032,ncbi_90249,ncbi_136647,ncbi_266722,ncbi_130888,ncbi_553158,ncbi_440829,ncbi_57578,ncbi_113451,ncbi_152098,ncbi_130733
miR-4483-y	28	ncbi_5896,ncbi_283726,ncbi_646851,ncbi_6517,ncbi_285268,ncbi_81491,ncbi_100526664,ncbi_100506127,ncbi_23349,ncbi_1645,ncbi_1950,ncbi_102724488,ncbi_119,ncbi_11076,ncbi_27242,ncbi_9717,ncbi_55586,ncbi_56648,ncbi_60529,ncbi_80032,ncbi_90249,ncbi_136647,ncbi_221336,ncbi_91749,ncbi_79846,ncbi_643669,ncbi_339541,ncbi_130733
miR-4485-y	9	ncbi_645121,ncbi_387763,ncbi_26298,ncbi_1396,ncbi_7837,ncbi_84814,ncbi_64170,ncbi_102724398,ncbi_342897
miR-451-x	9	ncbi_4739,ncbi_857,ncbi_26298,ncbi_2494,ncbi_11148,ncbi_166336,ncbi_9037,ncbi_60676,ncbi_51435
miR-4516-x	45	ncbi_5317,ncbi_24,ncbi_4846,ncbi_3479,ncbi_54921,ncbi_645121,ncbi_26471,ncbi_5655,ncbi_79098,ncbi_79919,ncbi_8626,ncbi_8291,ncbi_6932,ncbi_338382,ncbi_857,ncbi_5137,ncbi_1520,ncbi_26298,ncbi_57535,ncbi_56936,ncbi_124590,ncbi_79574,ncbi_105375355,ncbi_27129,ncbi_158584,ncbi_2247,ncbi_445577,ncbi_1634,ncbi_1805,ncbi_2570,ncbi_9507,ncbi_3005,ncbi_4584,ncbi_5971,ncbi_9915,ncbi_51351,ncbi_55084,ncbi_79817,ncbi_89870,ncbi_85236,ncbi_286204,ncbi_56606,ncbi_339184,ncbi_8942,ncbi_51435
miR-466-y	69	ncbi_2200,ncbi_5317,ncbi_3479,ncbi_3589,ncbi_2562,ncbi_2914,ncbi_339761,ncbi_389840,ncbi_90427,ncbi_339184,ncbi_440603,ncbi_145781,ncbi_3172,ncbi_5142,ncbi_26471,ncbi_50940,ncbi_5655,ncbi_79098,ncbi_768239,ncbi_2786,ncbi_8821,ncbi_8626,ncbi_4739,ncbi_5328,ncbi_647024,ncbi_8942,ncbi_834,ncbi_5570,ncbi_5271,ncbi_2

MiRNA	Target number	Target gene
		044,ncbi_4261,ncbi_171024,ncbi_395,ncbi_389602,ncbi_999,ncbi_2247,ncbi_166336,ncbi_1490,ncbi_3575,ncbi_3625,ncbi_3641,ncbi_5029,ncbi_5105,ncbi_8633,ncbi_8740,ncbi_2662,ncbi_9568,ncbi_4320,ncbi_10107,ncbi_2902,ncbi_23452,ncbi_51351,ncbi_57530,ncbi_64063,ncbi_10129,ncbi_79628,ncbi_79817,ncbi_81606,ncbi_84443,ncbi_153478,ncbi_254228,ncbi_286204,ncbi_91947,ncbi_6932,ncbi_54625,ncbi_57462,ncbi_25903,ncbi_343990,ncbi_51435
miR-5119-y	6	ncbi_6469,ncbi_387763,ncbi_2494,ncbi_30811,ncbi_55106,ncbi_2901
miR-542-y	29	ncbi_135,ncbi_646851,ncbi_92270,ncbi_55876,ncbi_80852,ncbi_285268,ncbi_84561,ncbi_100526664,ncbi_220108,ncbi_25850,ncbi_84679,ncbi_100506127,ncbi_23107,ncbi_1645,ncbi_340351,ncbi_6004,ncbi_8635,ncbi_9658,ncbi_51626,ncbi_56605,ncbi_57161,ncbi_80032,ncbi_136647,ncbi_91749,ncbi_144132,ncbi_196500,ncbi_266722,ncbi_84449,ncbi_785
miR-615-y	15	ncbi_94,ncbi_2914,ncbi_116372,ncbi_8479,ncbi_53615,ncbi_4261,ncbi_3669,ncbi_10659,ncbi_5579,ncbi_10158,ncbi_64063,ncbi_286204,ncbi_23452,ncbi_285489,ncbi_3134
miR-6240-x	31	ncbi_2099,ncbi_2155,ncbi_3479,ncbi_1141,ncbi_2914,ncbi_145781,ncbi_5271,ncbi_120939,ncbi_6925,ncbi_2786,ncbi_124976,ncbi_141,ncbi_285966,ncbi_9214,ncbi_5137,ncbi_8942,ncbi_834,ncbi_2044,ncbi_150372,ncbi_2247,ncbi_8456,ncbi_5579,ncbi_8436,ncbi_10107,ncbi_23105,ncbi_57530,ncbi_140893,ncbi_254228,ncbi_348013,ncbi_114769,ncbi_55106
miR-6412-y	1	ncbi_79628
miR-671-x	21	ncbi_2914,ncbi_5143,ncbi_342897,ncbi_2707,ncbi_285966,ncbi_4776,ncbi_999,ncbi_150372,ncbi_55124,ncbi_138050,ncbi_3965,ncbi_5105,ncbi_3037,ncbi_10991,ncbi_25759,ncbi_3122,ncbi_60676,ncbi_7473,ncbi_4261,ncbi_9542,ncbi_5672
miR-7-x	23	ncbi_785,ncbi_152098,ncbi_284194,ncbi_339210,ncbi_285268,ncbi_285755,ncbi_84439,ncbi_1638,ncbi_119,ncbi_283710,ncbi_100131539,ncbi_100506127,ncbi_9331,ncbi_6004,ncbi_10004,ncbi_84249,ncbi_136647,ncbi_138065,ncbi_91749,ncbi_220108,ncbi_1768,ncbi_266722,ncbi_374739
miR-709-y	74	ncbi_2200,ncbi_4261,ncbi_1589,ncbi_3479,ncbi_3589,ncbi_1141,ncbi_5345,ncbi_342897,ncbi_440603,ncbi_6241,ncbi_26471,ncbi_768239,ncbi_2786,ncbi_171024,ncbi_246,ncbi_55765,ncbi_387787,ncbi_11309,ncbi_7185,ncbi_8942,ncbi_1520,ncbi_57535,ncbi_2494,ncbi_53615,ncbi_2044,ncbi_124590,ncbi_8764,ncbi_140731,ncbi_9507,ncbi_6518,ncbi_79788,ncbi_1735,ncbi_445577,ncbi_283298,ncbi_166336,ncbi_3604,ncbi_202,ncbi_2570,ncbi_3014,ncbi_4148,ncbi_5029,ncbi_6367,ncbi_9037,ncbi_9283,ncbi_4584,ncbi_25759,ncbi_27254,ncbi_30811,ncbi_9770,ncbi_54625,ncbi_55742,ncbi_56649,ncbi_57462,ncbi_5239,ncbi_79628,ncbi_7473,ncbi_83729,ncbi_85236,ncbi_79987,ncbi_80274,ncbi_286204,ncbi_64856,ncbi_80737,ncbi_56606,ncbi_6932,ncbi_2263,ncbi_4773,ncbi_4599,ncbi_731220,ncbi_5745,ncbi_153478,ncbi_389602,ncbi_57101,ncbi_81493
miR-7550-x	25	ncbi_6556,ncbi_624,ncbi_5345,ncbi_645121,ncbi_321,ncbi_131578,ncbi_149478,ncbi_53841,ncbi_768,ncbi_376267,ncbi_3860,ncbi_9283,ncbi_7837,ncbi_26040,ncbi_8862,ncbi_54625,ncbi_57530,ncbi_64856,ncbi_7473,ncbi_161753,ncbi_102724398,ncbi_79173,ncbi_25903,ncbi_146547,ncbi_6578
miR-7669-y	64	ncbi_286,ncbi_1188,ncbi_2155,ncbi_5317,ncbi_6556,ncbi_3589,ncbi_3352,ncbi_342897,ncbi_164395,ncbi_51151,ncbi_6536,ncbi_1187,ncbi_120939,ncbi_149461,ncbi_171024,ncbi_246,ncbi_1015,ncbi_285489,ncbi_2521,ncbi_100134444,ncbi_8479,ncbi_4057,ncbi_8942,ncbi_1520,ncbi_57535,ncbi_2494,ncbi_51421,ncbi_53615,ncbi_1436,ncbi_101928841,ncbi_150372,ncbi_3965,ncbi_138050,ncbi_445577,ncbi_202,ncbi_6367,ncbi_8784,ncbi_2662,ncbi_9507,ncbi_10107,ncbi_10991,ncbi_23581,ncbi_27076,ncbi_27254,ncbi_9770,ncbi_9915,ncbi_23237,ncbi_51764,ncbi_79628,ncbi_81606,ncbi_84443,ncbi_153478,ncbi_80274,ncbi_145501,ncbi_80737,ncbi_55106,ncbi_2902,ncbi_4261,ncbi_2570,ncbi_340061,ncbi_9542,ncbi_57571,ncbi_1141,ncbi_56606
miR-7792-y	2	ncbi_445577,ncbi_56606

MiRNA	Target number	Target gene
miR-7977-x	18	ncbi_5143,ncbi_5345,ncbi_116372,ncbi_768239,ncbi_171024,ncbi_6932,ncbi_387763,ncbi_53615,ncbi_112399,ncbi_6518,ncbi_3005,ncbi_4320,ncbi_51286,ncbi_8862,ncbi_114897,ncbi_80274,ncbi_102724398,ncbi_107985729
miR-8112-y	41	ncbi_286,ncbi_2563,ncbi_2902,ncbi_2914,ncbi_5345,ncbi_90427,ncbi_26471,ncbi_768239,ncbi_2786,ncbi_88,ncbi_7161,ncbi_338382,ncbi_285489,ncbi_100134444,ncbi_10381,ncbi_8681,ncbi_7433,ncbi_3965,ncbi_100129484,ncbi_1805,ncbi_3014,ncbi_9568,ncbi_8971,ncbi_25759,ncbi_30811,ncbi_9915,ncbi_26040,ncbi_51330,ncbi_56241,ncbi_81606,ncbi_114897,ncbi_286204,ncbi_145501,ncbi_348013,ncbi_64856,ncbi_55512,ncbi_4773,ncbi_9542,ncbi_25903,ncbi_3134,ncbi_105369535
miR-8528-x	5	ncbi_5346,ncbi_728392,ncbi_939,ncbi_266743,ncbi_11076
miR-873-x	48	ncbi_2099,ncbi_3561,ncbi_624,ncbi_342897,ncbi_90427,ncbi_440603,ncbi_10659,ncbi_26471,ncbi_79098,ncbi_4776,ncbi_6866,ncbi_8942,ncbi_26298,ncbi_56936,ncbi_2044,ncbi_11148,ncbi_54587,ncbi_1087,ncbi_57188,ncbi_101928841,ncbi_8839,ncbi_6518,ncbi_342667,ncbi_445577,ncbi_166336,ncbi_4148,ncbi_5579,ncbi_5880,ncbi_8436,ncbi_9507,ncbi_23581,ncbi_51703,ncbi_60676,ncbi_64284,ncbi_79628,ncbi_84962,ncbi_127707,ncbi_79987,ncbi_254228,ncbi_128209,ncbi_348013,ncbi_4739,ncbi_8638,ncbi_55106,ncbi_4773,ncbi_1141,ncbi_3134,ncbi_284021
miR-8824-y	40	ncbi_24,ncbi_1734,ncbi_5143,ncbi_6531,ncbi_50940,ncbi_5655,ncbi_56649,ncbi_347454,ncbi_171024,ncbi_26040,ncbi_321,ncbi_11309,ncbi_440689,ncbi_285489,ncbi_57571,ncbi_8942,ncbi_26298,ncbi_2044,ncbi_389602,ncbi_27071,ncbi_376267,ncbi_5029,ncbi_5105,ncbi_5579,ncbi_6585,ncbi_8633,ncbi_8436,ncbi_23452,ncbi_9379,ncbi_51351,ncbi_10060,ncbi_84443,ncbi_254228,ncbi_4739,ncbi_153478,ncbi_8835,ncbi_64284,ncbi_51435,ncbi_105371921,ncbi_158584
miR-9226-y	21	ncbi_1188,ncbi_2200,ncbi_3589,ncbi_2914,ncbi_653145,ncbi_1187,ncbi_728113,ncbi_2521,ncbi_56936,ncbi_5271,ncbi_1087,ncbi_10659,ncbi_5652,ncbi_6585,ncbi_9915,ncbi_55742,ncbi_89870,ncbi_149461,ncbi_128209,ncbi_8638,ncbi_4135
miR-9277-y	57	ncbi_554,ncbi_2263,ncbi_3589,ncbi_2914,ncbi_5143,ncbi_5345,ncbi_339761,ncbi_145781,ncbi_10659,ncbi_79098,ncbi_79919,ncbi_7430,ncbi_5673,ncbi_79674,ncbi_7185,ncbi_8942,ncbi_834,ncbi_729220,ncbi_3773,ncbi_5672,ncbi_2044,ncbi_54587,ncbi_221468,ncbi_4261,ncbi_55224,ncbi_2247,ncbi_2570,ncbi_3641,ncbi_3725,ncbi_5029,ncbi_5105,ncbi_6367,ncbi_6585,ncbi_8740,ncbi_9037,ncbi_1463,ncbi_9507,ncbi_26040,ncbi_26049,ncbi_55561,ncbi_55742,ncbi_55512,ncbi_56241,ncbi_56649,ncbi_79628,ncbi_79817,ncbi_83547,ncbi_3017,ncbi_126868,ncbi_91947,ncbi_3134,ncbi_105373347,ncbi_653145,ncbi_1141,ncbi_729857,ncbi_51435,ncbi_105369535
miR-93-y	34	ncbi_1141,ncbi_1435,ncbi_653145,ncbi_8710,ncbi_120939,ncbi_728113,ncbi_51062,ncbi_171024,ncbi_285966,ncbi_26298,ncbi_834,ncbi_9415,ncbi_58189,ncbi_4261,ncbi_283229,ncbi_6518,ncbi_138050,ncbi_1212,ncbi_445577,ncbi_166336,ncbi_1463,ncbi_10107,ncbi_23371,ncbi_57462,ncbi_7473,ncbi_80274,ncbi_348013,ncbi_136306,ncbi_2766,ncbi_7433,ncbi_55084,ncbi_25903,ncbi_105369535,ncbi_158584
miR-939-x	72	ncbi_286,ncbi_1734,ncbi_2914,ncbi_5345,ncbi_164395,ncbi_643866,ncbi_4222,ncbi_26471,ncbi_5655,ncbi_171024,ncbi_6932,ncbi_4778,ncbi_284422,ncbi_643382,ncbi_9214,ncbi_2859,ncbi_10381,ncbi_8942,ncbi_63924,ncbi_30851,ncbi_57535,ncbi_5672,ncbi_54587,ncbi_4261,ncbi_6512,ncbi_84962,ncbi_8764,ncbi_340061,ncbi_79574,ncbi_58476,ncbi_55577,ncbi_27129,ncbi_342667,ncbi_128414,ncbi_1212,ncbi_445577,ncbi_283298,ncbi_250,ncbi_1464,ncbi_2901,ncbi_5029,ncbi_7134,ncbi_8740,ncbi_9283,ncbi_11131,ncbi_11240,ncbi_23581,ncbi_27076,ncbi_9770,ncbi_51702,ncbi_51286,ncbi_51330,ncbi_55107,ncbi_55742,ncbi_56241,ncbi_10060,ncbi_64856,ncbi_81606,ncbi_251,ncbi_84443,ncbi_153478,ncbi_91703,ncbi_254228,ncbi_100996758,ncbi_23452,ncbi_285489,ncbi_56606,ncbi_5540,ncbi_3134,ncbi_57101,ncbi_8835,ncbi_107984590
miR-96-x	19	ncbi_286223,ncbi_2824,ncbi_84439,ncbi_388650,ncbi_110116772,ncbi_5017,ncbi_10004,ncbi_11076,ncbi_55244,ncbi_128434,ncbi_138065,ncbi_105374013,ncbi_266722,ncbi_23302,ncbi_285755,ncbi_100289279,ncbi_113451,ncbi_1768,ncbi_55840

MiRNA	Target number	Target gene
miR-9993-y	13	ncbi_3172,ncbi_8542,ncbi_4261,ncbi_101928841,ncbi_79788,ncbi_250,ncbi_2570,ncbi_3625,ncbi_153478,ncbi_4599,ncbi_23105,ncbi_25903,ncbi_80008
novel-m0013-5p	29	ncbi_3479,ncbi_2562,ncbi_5143,ncbi_145781,ncbi_976,ncbi_3172,ncbi_8626,ncbi_51062,ncbi_171024,ncbi_5673,ncbi_6518,ncbi_25780,ncbi_387787,ncbi_4015,ncbi_8479,ncbi_1520,ncbi_51421,ncbi_5540,ncbi_100996758,ncbi_112399,ncbi_10659,ncbi_1634,ncbi_3371,ncbi_5268,ncbi_51764,ncbi_91947,ncbi_80737,ncbi_56606,ncbi_80008
novel-m0049-5p	48	ncbi_2155,ncbi_2200,ncbi_1439,ncbi_339184,ncbi_440603,ncbi_27122,ncbi_120939,ncbi_79098,ncbi_6925,ncbi_2786,ncbi_347454,ncbi_171024,ncbi_131578,ncbi_4739,ncbi_63901,ncbi_1015,ncbi_4599,ncbi_9058,ncbi_7185,ncbi_5137,ncbi_8479,ncbi_26298,ncbi_834,ncbi_2044,ncbi_2247,ncbi_1634,ncbi_3625,ncbi_1463,ncbi_10893,ncbi_9770,ncbi_23371,ncbi_10060,ncbi_60676,ncbi_64856,ncbi_10129,ncbi_79442,ncbi_79628,ncbi_153478,ncbi_254228,ncbi_4135,ncbi_56606,ncbi_23452,ncbi_2901,ncbi_25903,ncbi_343990,ncbi_729857,ncbi_3479,ncbi_57188
novel-m0050-5p	48	ncbi_2155,ncbi_2200,ncbi_1439,ncbi_339184,ncbi_440603,ncbi_27122,ncbi_120939,ncbi_79098,ncbi_6925,ncbi_2786,ncbi_347454,ncbi_171024,ncbi_131578,ncbi_4739,ncbi_63901,ncbi_1015,ncbi_4599,ncbi_9058,ncbi_7185,ncbi_5137,ncbi_8479,ncbi_26298,ncbi_834,ncbi_2044,ncbi_2247,ncbi_1634,ncbi_3625,ncbi_1463,ncbi_10893,ncbi_9770,ncbi_23371,ncbi_10060,ncbi_60676,ncbi_64856,ncbi_10129,ncbi_79442,ncbi_79628,ncbi_153478,ncbi_254228,ncbi_4135,ncbi_56606,ncbi_23452,ncbi_2901,ncbi_25903,ncbi_343990,ncbi_729857,ncbi_3479,ncbi_57188
novel-m0051-5p	48	ncbi_2155,ncbi_2200,ncbi_1439,ncbi_339184,ncbi_440603,ncbi_27122,ncbi_120939,ncbi_79098,ncbi_6925,ncbi_2786,ncbi_347454,ncbi_171024,ncbi_131578,ncbi_4739,ncbi_63901,ncbi_1015,ncbi_4599,ncbi_9058,ncbi_7185,ncbi_5137,ncbi_8479,ncbi_26298,ncbi_834,ncbi_2044,ncbi_2247,ncbi_1634,ncbi_3625,ncbi_1463,ncbi_10893,ncbi_9770,ncbi_23371,ncbi_10060,ncbi_60676,ncbi_64856,ncbi_10129,ncbi_79442,ncbi_79628,ncbi_153478,ncbi_254228,ncbi_4135,ncbi_56606,ncbi_23452,ncbi_2901,ncbi_25903,ncbi_343990,ncbi_729857,ncbi_3479,ncbi_57188
novel-m0052-5p	48	ncbi_2155,ncbi_2200,ncbi_1439,ncbi_339184,ncbi_440603,ncbi_27122,ncbi_120939,ncbi_79098,ncbi_6925,ncbi_2786,ncbi_347454,ncbi_171024,ncbi_131578,ncbi_4739,ncbi_63901,ncbi_1015,ncbi_4599,ncbi_9058,ncbi_7185,ncbi_5137,ncbi_8479,ncbi_26298,ncbi_834,ncbi_2044,ncbi_2247,ncbi_1634,ncbi_3625,ncbi_1463,ncbi_10893,ncbi_9770,ncbi_23371,ncbi_10060,ncbi_60676,ncbi_64856,ncbi_10129,ncbi_79442,ncbi_79628,ncbi_153478,ncbi_254228,ncbi_4135,ncbi_56606,ncbi_23452,ncbi_2901,ncbi_25903,ncbi_343990,ncbi_729857,ncbi_3479,ncbi_57188
novel-m0053-3p	10	ncbi_1308,ncbi_10659,ncbi_3172,ncbi_7161,ncbi_3242,ncbi_84699,ncbi_55742,ncbi_56241,ncbi_64856,ncbi_105369535
novel-m0067-3p	28	ncbi_4143,ncbi_18,ncbi_401265,ncbi_6752,ncbi_285268,ncbi_255809,ncbi_81491,ncbi_1950,ncbi_2244,ncbi_119,ncbi_349565,ncbi_196500,ncbi_257019,ncbi_144321,ncbi_56704,ncbi_57578,ncbi_1645,ncbi_340351,ncbi_8635,ncbi_27242,ncbi_56605,ncbi_85480,ncbi_136647,ncbi_144132,ncbi_9658,ncbi_100289279,ncbi_266722,ncbi_130733
novel-m0069-5p	29	ncbi_3479,ncbi_2562,ncbi_5143,ncbi_145781,ncbi_976,ncbi_3172,ncbi_8626,ncbi_51062,ncbi_171024,ncbi_5673,ncbi_6518,ncbi_25780,ncbi_387787,ncbi_4015,ncbi_8479,ncbi_1520,ncbi_51421,ncbi_5540,ncbi_100996758,ncbi_112399,ncbi_10659,ncbi_1634,ncbi_3371,ncbi_5268,ncbi_51764,ncbi_91947,ncbi_80737,ncbi_56606,ncbi_80008
novel-m0079-3p	32	ncbi_2155,ncbi_2200,ncbi_2562,ncbi_5143,ncbi_84632,ncbi_653145,ncbi_6925,ncbi_728113,ncbi_88,ncbi_79931,ncbi_171024,ncbi_6932,ncbi_1015,ncbi_857,ncbi_241,ncbi_145447,ncbi_140731,ncbi_376267,ncbi_163782,ncbi_202,ncbi_1634,ncbi_8633,ncbi_3037,ncbi_3702,ncbi_30811,ncbi_9770,ncbi_10060,ncbi_80201,ncbi_91752,ncbi_100996758,ncbi_5540,ncbi_25903
novel-m0081-5p	17	ncbi_785,ncbi_646851,ncbi_55186,ncbi_285755,ncbi_119,ncbi_25850,ncbi_84679,ncbi_84439,ncbi_100129924,ncbi_140564,ncbi_9717,ncbi_23563,ncbi_136647,ncbi_285349,ncbi_55840,ncbi_134526,ncbi_107985678
novel-m0084-5p	48	ncbi_2155,ncbi_2200,ncbi_1439,ncbi_339184,ncbi_440603,ncbi_27122,ncbi_120939,ncbi_79098,ncbi_6925,ncbi_2786,ncbi_347454,ncbi_171024,ncbi_131578,ncbi_4739,nc

MiRNA	Target number	Target gene
		bi_63901,ncbi_1015,ncbi_4599,ncbi_9058,ncbi_7185,ncbi_5137,ncbi_8479,ncbi_26298,ncbi_834,ncbi_2044,ncbi_2247,ncbi_1634,ncbi_3625,ncbi_1463,ncbi_10893,ncbi_9770,ncbi_23371,ncbi_10060,ncbi_60676,ncbi_64856,ncbi_10129,ncbi_79442,ncbi_79628,ncbi_153478,ncbi_254228,ncbi_4135,ncbi_56606,ncbi_23452,ncbi_2901,ncbi_25903,ncbi_343990,ncbi_729857,ncbi_3479,ncbi_57188
novel-m0086-5p	48	ncbi_2155,ncbi_2200,ncbi_1439,ncbi_339184,ncbi_440603,ncbi_27122,ncbi_120939,ncbi_79098,ncbi_6925,ncbi_2786,ncbi_347454,ncbi_171024,ncbi_131578,ncbi_4739,ncbi_63901,ncbi_1015,ncbi_4599,ncbi_9058,ncbi_7185,ncbi_5137,ncbi_8479,ncbi_26298,ncbi_834,ncbi_2044,ncbi_2247,ncbi_1634,ncbi_3625,ncbi_1463,ncbi_10893,ncbi_9770,ncbi_23371,ncbi_10060,ncbi_60676,ncbi_64856,ncbi_10129,ncbi_79442,ncbi_79628,ncbi_153478,ncbi_254228,ncbi_4135,ncbi_56606,ncbi_23452,ncbi_2901,ncbi_25903,ncbi_343990,ncbi_729857,ncbi_3479,ncbi_57188
novel-m0090-5p	33	ncbi_3479,ncbi_339761,ncbi_51151,ncbi_1261,ncbi_2786,ncbi_647024,ncbi_4790,ncbi_7185,ncbi_4261,ncbi_387787,ncbi_158584,ncbi_1805,ncbi_5029,ncbi_5105,ncbi_5579,ncbi_8519,ncbi_27063,ncbi_27289,ncbi_51351,ncbi_8862,ncbi_10129,ncbi_79442,ncbi_79628,ncbi_81606,ncbi_1435,ncbi_91752,ncbi_3017,ncbi_6932,ncbi_55084,ncbi_1141,ncbi_343990,ncbi_729857,ncbi_56606
novel-m0093-5p	48	ncbi_2155,ncbi_2200,ncbi_1439,ncbi_339184,ncbi_440603,ncbi_27122,ncbi_120939,ncbi_79098,ncbi_6925,ncbi_2786,ncbi_347454,ncbi_171024,ncbi_131578,ncbi_4739,ncbi_63901,ncbi_1015,ncbi_4599,ncbi_9058,ncbi_7185,ncbi_5137,ncbi_8479,ncbi_26298,ncbi_834,ncbi_2044,ncbi_2247,ncbi_1634,ncbi_3625,ncbi_1463,ncbi_10893,ncbi_9770,ncbi_23371,ncbi_10060,ncbi_60676,ncbi_64856,ncbi_10129,ncbi_79442,ncbi_79628,ncbi_153478,ncbi_254228,ncbi_4135,ncbi_56606,ncbi_23452,ncbi_2901,ncbi_25903,ncbi_343990,ncbi_729857,ncbi_3479,ncbi_57188
novel-m0095-5p	80	ncbi_94,ncbi_286,ncbi_3914,ncbi_4261,ncbi_1439,ncbi_1589,ncbi_6556,ncbi_3486,ncbi_1141,ncbi_2914,ncbi_339761,ncbi_51151,ncbi_27122,ncbi_6536,ncbi_220979,ncbi_5655,ncbi_79098,ncbi_6925,ncbi_7161,ncbi_171024,ncbi_55124,ncbi_4773,ncbi_4776,ncbi_284422,ncbi_81493,ncbi_338382,ncbi_5137,ncbi_401934,ncbi_84699,ncbi_53615,ncbi_9415,ncbi_93082,ncbi_395,ncbi_55224,ncbi_285489,ncbi_1396,ncbi_158248,ncbi_9507,ncbi_5159,ncbi_1735,ncbi_250,ncbi_1634,ncbi_5105,ncbi_5880,ncbi_8581,ncbi_8633,ncbi_9037,ncbi_1463,ncbi_9283,ncbi_3108,ncbi_10107,ncbi_10991,ncbi_11240,ncbi_25759,ncbi_9915,ncbi_9379,ncbi_51286,ncbi_55561,ncbi_55512,ncbi_79817,ncbi_81606,ncbi_251,ncbi_84814,ncbi_126868,ncbi_127707,ncbi_147138,ncbi_80274,ncbi_4739,ncbi_158584,ncbi_102724398,ncbi_56606,ncbi_221527,ncbi_2902,ncbi_5239,ncbi_4599,ncbi_9542,ncbi_4359,ncbi_343990,ncbi_51435,ncbi_80008
novel-m0111-5p	5	ncbi_10659,ncbi_7837,ncbi_64856,ncbi_102724398,ncbi_2099
novel-m0119-3p	31	ncbi_2562,ncbi_5724,ncbi_3172,ncbi_5142,ncbi_54921,ncbi_8626,ncbi_171024,ncbi_2521,ncbi_4015,ncbi_5137,ncbi_395,ncbi_376267,ncbi_999,ncbi_79788,ncbi_285966,ncbi_3604,ncbi_202,ncbi_3371,ncbi_6585,ncbi_7805,ncbi_30811,ncbi_26049,ncbi_51703,ncbi_55512,ncbi_10129,ncbi_79628,ncbi_79983,ncbi_122402,ncbi_4739,ncbi_105373347,ncbi_5672
novel-m0124-5p	11	ncbi_2902,ncbi_6536,ncbi_8542,ncbi_53841,ncbi_1436,ncbi_83882,ncbi_3014,ncbi_23237,ncbi_64856,ncbi_102724398,ncbi_55765
novel-m0126-5p	48	ncbi_2155,ncbi_2200,ncbi_1439,ncbi_339184,ncbi_440603,ncbi_27122,ncbi_120939,ncbi_79098,ncbi_6925,ncbi_2786,ncbi_347454,ncbi_171024,ncbi_131578,ncbi_4739,ncbi_63901,ncbi_1015,ncbi_4599,ncbi_9058,ncbi_7185,ncbi_5137,ncbi_8479,ncbi_26298,ncbi_834,ncbi_2044,ncbi_2247,ncbi_1634,ncbi_3625,ncbi_1463,ncbi_10893,ncbi_9770,ncbi_23371,ncbi_10060,ncbi_60676,ncbi_64856,ncbi_10129,ncbi_79442,ncbi_79628,ncbi_153478,ncbi_254228,ncbi_4135,ncbi_56606,ncbi_23452,ncbi_2901,ncbi_25903,ncbi_343990,ncbi_729857,ncbi_3479,ncbi_57188
novel-m0142-5p	48	ncbi_2155,ncbi_2200,ncbi_1439,ncbi_339184,ncbi_440603,ncbi_27122,ncbi_120939,ncbi_79098,ncbi_6925,ncbi_2786,ncbi_347454,ncbi_171024,ncbi_131578,ncbi_4739,ncbi_63901,ncbi_1015,ncbi_4599,ncbi_9058,ncbi_7185,ncbi_5137,ncbi_8479,ncbi_26298,ncbi_834,ncbi_2044,ncbi_2247,ncbi_1634,ncbi_3625,ncbi_1463,ncbi_10893,ncbi_97

MiRNA	Target number	Target gene
		70,ncbi_23371,ncbi_10060,ncbi_60676,ncbi_64856,ncbi_10129,ncbi_79442,ncbi_79628,ncbi_153478,ncbi_254228,ncbi_4135,ncbi_56606,ncbi_23452,ncbi_2901,ncbi_25903,ncbi_343990,ncbi_729857,ncbi_3479,ncbi_57188
novel-m0157-5p	14	ncbi_286223,ncbi_2824,ncbi_401265,ncbi_6517,ncbi_6752,ncbi_119,ncbi_23349,ncbi_84142,ncbi_100506127,ncbi_5577,ncbi_84249,ncbi_221336,ncbi_643669,ncbi_92270
novel-m0159-5p	14	ncbi_401265,ncbi_118490,ncbi_9901,ncbi_80852,ncbi_84679,ncbi_221336,ncbi_100506127,ncbi_102724488,ncbi_84249,ncbi_143282,ncbi_79846,ncbi_196500,ncbi_18,ncbi_84700
novel-m0169-5p	48	ncbi_2155,ncbi_2200,ncbi_1439,ncbi_339184,ncbi_440603,ncbi_27122,ncbi_120939,ncbi_79098,ncbi_6925,ncbi_2786,ncbi_347454,ncbi_171024,ncbi_131578,ncbi_4739,ncbi_63901,ncbi_1015,ncbi_4599,ncbi_9058,ncbi_7185,ncbi_5137,ncbi_8479,ncbi_26298,ncbi_834,ncbi_2044,ncbi_2247,ncbi_1634,ncbi_3625,ncbi_1463,ncbi_10893,ncbi_9770,ncbi_23371,ncbi_10060,ncbi_60676,ncbi_64856,ncbi_10129,ncbi_79442,ncbi_79628,ncbi_153478,ncbi_254228,ncbi_4135,ncbi_56606,ncbi_23452,ncbi_2901,ncbi_25903,ncbi_343990,ncbi_729857,ncbi_3479,ncbi_57188
novel-m0186-5p	33	ncbi_18,ncbi_92270,ncbi_151556,ncbi_6517,ncbi_55186,ncbi_23213,ncbi_57834,ncbi_6565,ncbi_2244,ncbi_84561,ncbi_220108,ncbi_201181,ncbi_100506127,ncbi_84449,ncbi_152189,ncbi_55062,ncbi_84439,ncbi_8635,ncbi_7025,ncbi_9658,ncbi_9717,ncbi_55150,ncbi_56605,ncbi_60529,ncbi_23563,ncbi_80032,ncbi_81558,ncbi_285349,ncbi_171425,ncbi_196500,ncbi_84700,ncbi_285755,ncbi_1768
novel-m0209-3p	34	ncbi_2200,ncbi_3172,ncbi_440603,ncbi_2786,ncbi_54866,ncbi_4776,ncbi_8605,ncbi_79674,ncbi_284297,ncbi_8942,ncbi_10752,ncbi_124590,ncbi_171024,ncbi_84962,ncbi_8764,ncbi_2901,ncbi_999,ncbi_150372,ncbi_1735,ncbi_3604,ncbi_5880,ncbi_10158,ncbi_30811,ncbi_9770,ncbi_54625,ncbi_10129,ncbi_127707,ncbi_254228,ncbi_284340,ncbi_80737,ncbi_136306,ncbi_55106,ncbi_23452,ncbi_57571
novel-m0212-5p	17	ncbi_5345,ncbi_4648,ncbi_768239,ncbi_4776,ncbi_100134444,ncbi_5159,ncbi_3575,ncbi_10991,ncbi_7837,ncbi_55742,ncbi_147138,ncbi_80274,ncbi_348013,ncbi_221468,ncbi_1307,ncbi_158584,ncbi_284434
novel-m0214-3p	29	ncbi_3479,ncbi_2562,ncbi_3352,ncbi_5655,ncbi_284021,ncbi_445577,ncbi_8821,ncbi_5672,ncbi_57101,ncbi_340061,ncbi_150372,ncbi_5271,ncbi_5159,ncbi_1490,ncbi_5579,ncbi_8740,ncbi_6578,ncbi_11240,ncbi_7462,ncbi_79628,ncbi_79817,ncbi_348013,ncbi_4600,ncbi_4599,ncbi_8835,ncbi_23105,ncbi_51435,ncbi_140711,ncbi_80008
novel-m0224-5p	24	ncbi_27293,ncbi_220979,ncbi_768239,ncbi_5673,ncbi_131578,ncbi_6518,ncbi_2263,ncbi_8942,ncbi_26298,ncbi_2494,ncbi_2044,ncbi_8076,ncbi_4600,ncbi_8740,ncbi_2662,ncbi_6578,ncbi_25759,ncbi_27289,ncbi_51703,ncbi_56241,ncbi_57530,ncbi_254228,ncbi_4773,ncbi_25903
novel-m0225-5p	24	ncbi_27293,ncbi_220979,ncbi_768239,ncbi_5673,ncbi_131578,ncbi_6518,ncbi_2263,ncbi_8942,ncbi_26298,ncbi_2494,ncbi_2044,ncbi_8076,ncbi_4600,ncbi_8740,ncbi_2662,ncbi_6578,ncbi_25759,ncbi_27289,ncbi_51703,ncbi_56241,ncbi_57530,ncbi_254228,ncbi_4773,ncbi_25903
novel-m0226-5p	24	ncbi_27293,ncbi_220979,ncbi_768239,ncbi_5673,ncbi_131578,ncbi_6518,ncbi_2263,ncbi_8942,ncbi_26298,ncbi_2494,ncbi_2044,ncbi_8076,ncbi_4600,ncbi_8740,ncbi_2662,ncbi_6578,ncbi_25759,ncbi_27289,ncbi_51703,ncbi_56241,ncbi_57530,ncbi_254228,ncbi_4773,ncbi_25903
novel-m0237-5p	24	ncbi_27293,ncbi_220979,ncbi_768239,ncbi_5673,ncbi_131578,ncbi_6518,ncbi_2263,ncbi_8942,ncbi_26298,ncbi_2494,ncbi_2044,ncbi_8076,ncbi_4600,ncbi_8740,ncbi_2662,ncbi_6578,ncbi_25759,ncbi_27289,ncbi_51703,ncbi_56241,ncbi_57530,ncbi_254228,ncbi_4773,ncbi_25903
novel-m0238-5p	24	ncbi_27293,ncbi_220979,ncbi_768239,ncbi_5673,ncbi_131578,ncbi_6518,ncbi_2263,ncbi_8942,ncbi_26298,ncbi_2494,ncbi_2044,ncbi_8076,ncbi_4600,ncbi_8740,ncbi_2662,ncbi_6578,ncbi_25759,ncbi_27289,ncbi_51703,ncbi_56241,ncbi_57530,ncbi_254228,ncbi_4773,ncbi_25903
novel-m0245-3p	57	ncbi_2155,ncbi_624,ncbi_3589,ncbi_2562,ncbi_2914,ncbi_5143,ncbi_342897,ncbi_339761,ncbi_5047,ncbi_140453,ncbi_8710,ncbi_26471,ncbi_120939,ncbi_768239,ncbi_2

MiRNA	Target number	Target gene
		825,ncbi_2318,ncbi_6932,ncbi_131578,ncbi_55765,ncbi_647024,ncbi_2521,ncbi_53841,ncbi_5137,ncbi_57535,ncbi_58189,ncbi_256158,ncbi_2099,ncbi_1212,ncbi_3604,ncbi_4148,ncbi_4600,ncbi_5744,ncbi_8519,ncbi_10148,ncbi_7062,ncbi_10870,ncbi_26040,ncbi_54625,ncbi_55084,ncbi_55742,ncbi_56901,ncbi_64856,ncbi_79817,ncbi_112464,ncbi_127294,ncbi_348013,ncbi_136306,ncbi_9542,ncbi_158248,ncbi_2902,ncbi_7433,ncbi_6536,ncbi_1141,ncbi_58484,ncbi_6578,ncbi_147138,ncbi_284021
novel-m0249-5p	47	ncbi_94,ncbi_5317,ncbi_1439,ncbi_3589,ncbi_1141,ncbi_5143,ncbi_339761,ncbi_389840,ncbi_10659,ncbi_6241,ncbi_645121,ncbi_2786,ncbi_55124,ncbi_563,ncbi_8479,ncbi_7433,ncbi_56936,ncbi_58189,ncbi_124590,ncbi_4261,ncbi_171024,ncbi_8764,ncbi_80008,ncbi_6518,ncbi_2247,ncbi_166336,ncbi_1307,ncbi_1490,ncbi_3371,ncbi_5652,ncbi_9037,ncbi_1463,ncbi_9283,ncbi_9507,ncbi_10107,ncbi_23581,ncbi_9379,ncbi_23105,ncbi_55561,ncbi_60676,ncbi_81706,ncbi_84814,ncbi_254228,ncbi_221527,ncbi_5239,ncbi_100507003,ncbi_2170
novel-m0275-5p	36	ncbi_2200,ncbi_90427,ncbi_284434,ncbi_5142,ncbi_645121,ncbi_5655,ncbi_1261,ncbi_2825,ncbi_124976,ncbi_131578,ncbi_4773,ncbi_81493,ncbi_2521,ncbi_1520,ncbi_10752,ncbi_59350,ncbi_8835,ncbi_2044,ncbi_171024,ncbi_395,ncbi_55577,ncbi_2247,ncbi_166336,ncbi_3604,ncbi_1634,ncbi_3702,ncbi_26049,ncbi_51351,ncbi_8862,ncbi_79817,ncbi_79983,ncbi_81706,ncbi_254228,ncbi_157855,ncbi_729857,ncbi_105369535
novel-m0291-5p	37	ncbi_3479,ncbi_1435,ncbi_339761,ncbi_51151,ncbi_5655,ncbi_1261,ncbi_2786,ncbi_171024,ncbi_647024,ncbi_4790,ncbi_7185,ncbi_64699,ncbi_4261,ncbi_387787,ncbi_158584,ncbi_1805,ncbi_5029,ncbi_5105,ncbi_5579,ncbi_7047,ncbi_8519,ncbi_27063,ncbi_27289,ncbi_51351,ncbi_8862,ncbi_10129,ncbi_79442,ncbi_79628,ncbi_81606,ncbi_153478,ncbi_128209,ncbi_91752,ncbi_3017,ncbi_55084,ncbi_343990,ncbi_729857,ncbi_56606
novel-m0292-5p	36	ncbi_2200,ncbi_90427,ncbi_284434,ncbi_5142,ncbi_645121,ncbi_5655,ncbi_1261,ncbi_2825,ncbi_124976,ncbi_131578,ncbi_4773,ncbi_81493,ncbi_2521,ncbi_1520,ncbi_10752,ncbi_59350,ncbi_8835,ncbi_2044,ncbi_171024,ncbi_395,ncbi_55577,ncbi_2247,ncbi_166336,ncbi_3604,ncbi_1634,ncbi_3702,ncbi_26049,ncbi_51351,ncbi_8862,ncbi_79817,ncbi_79983,ncbi_81706,ncbi_254228,ncbi_157855,ncbi_729857,ncbi_105369535
novel-m0297-5p	11	ncbi_151556,ncbi_1950,ncbi_84679,ncbi_100131539,ncbi_84071,ncbi_1645,ncbi_136647,ncbi_266722,ncbi_84700,ncbi_56605,ncbi_10219
novel-m0298-3p	26	ncbi_1734,ncbi_5143,ncbi_342897,ncbi_10659,ncbi_220979,ncbi_54921,ncbi_25780,ncbi_246,ncbi_55765,ncbi_8372,ncbi_171024,ncbi_395,ncbi_158248,ncbi_5029,ncbi_51351,ncbi_51435,ncbi_79628,ncbi_150372,ncbi_254228,ncbi_80274,ncbi_4135,ncbi_100134444,ncbi_166336,ncbi_7433,ncbi_57571,ncbi_153478
novel-m0328-3p	18	ncbi_4987,ncbi_119,ncbi_349565,ncbi_107080638,ncbi_100506127,ncbi_7700,ncbi_110116772,ncbi_1645,ncbi_197,ncbi_102724488,ncbi_5577,ncbi_2634,ncbi_11076,ncbi_27242,ncbi_57161,ncbi_136647,ncbi_56171,ncbi_90249
novel-m0345-5p	33	ncbi_18,ncbi_92270,ncbi_151556,ncbi_6517,ncbi_55186,ncbi_23213,ncbi_57834,ncbi_6565,ncbi_2244,ncbi_84561,ncbi_220108,ncbi_201181,ncbi_100506127,ncbi_84449,ncbi_152189,ncbi_55062,ncbi_84439,ncbi_8635,ncbi_7025,ncbi_9658,ncbi_9717,ncbi_55150,ncbi_56605,ncbi_60529,ncbi_23563,ncbi_80032,ncbi_81558,ncbi_285349,ncbi_171425,ncbi_196500,ncbi_84700,ncbi_285755,ncbi_1768
novel-m0351-5p	21	ncbi_2006,ncbi_164395,ncbi_729857,ncbi_171024,ncbi_1780,ncbi_246,ncbi_285489,ncbi_83690,ncbi_389602,ncbi_3669,ncbi_140731,ncbi_2170,ncbi_6585,ncbi_395,ncbi_9379,ncbi_114769,ncbi_2901,ncbi_105373347,ncbi_25903,ncbi_8942,ncbi_158584
novel-m0367-5p	26	ncbi_785,ncbi_4987,ncbi_6517,ncbi_284194,ncbi_7358,ncbi_100526664,ncbi_84679,ncbi_90527,ncbi_23107,ncbi_113451,ncbi_7700,ncbi_100129924,ncbi_2979,ncbi_726,ncbi_56605,ncbi_56648,ncbi_80032,ncbi_136647,ncbi_143282,ncbi_130888,ncbi_646851,ncbi_84449,ncbi_10841,ncbi_102724488,ncbi_339488,ncbi_112268350
novel-m0370-5p	48	ncbi_2155,ncbi_2200,ncbi_1439,ncbi_339184,ncbi_440603,ncbi_27122,ncbi_120939,ncbi_79098,ncbi_6925,ncbi_2786,ncbi_347454,ncbi_171024,ncbi_131578,ncbi_4739,nc

MiRNA	Target number	Target gene
		bi_63901,ncbi_1015,ncbi_4599,ncbi_9058,ncbi_7185,ncbi_5137,ncbi_8479,ncbi_26298,ncbi_834,ncbi_2044,ncbi_2247,ncbi_1634,ncbi_3625,ncbi_1463,ncbi_10893,ncbi_9770,ncbi_23371,ncbi_10060,ncbi_60676,ncbi_64856,ncbi_10129,ncbi_79442,ncbi_79628,ncbi_153478,ncbi_254228,ncbi_4135,ncbi_56606,ncbi_23452,ncbi_2901,ncbi_25903,ncbi_343990,ncbi_729857,ncbi_3479,ncbi_57188
novel-m0390-3p	2	ncbi_7296,ncbi_90527
novel-m0424-5p	7	ncbi_80852,ncbi_55186,ncbi_23213,ncbi_220108,ncbi_257019,ncbi_100129924,ncbi_105374013
novel-m0441-5p	24	ncbi_3576,ncbi_4846,ncbi_624,ncbi_124976,ncbi_285966,ncbi_8605,ncbi_57535,ncbi_221468,ncbi_4261,ncbi_80345,ncbi_6518,ncbi_84941,ncbi_2247,ncbi_7471,ncbi_10893,ncbi_2902,ncbi_9770,ncbi_80274,ncbi_128209,ncbi_348013,ncbi_4135,ncbi_256158,ncbi_105373347,ncbi_107985729
novel-m0452-5p	6	ncbi_286223,ncbi_196500,ncbi_7700,ncbi_143282,ncbi_440829,ncbi_220108
novel-m0476-3p	8	ncbi_4846,ncbi_53841,ncbi_285489,ncbi_3014,ncbi_7429,ncbi_11240,ncbi_25823,ncbi_55512
novel-m0485-5p	1	ncbi_728392
novel-m0487-3p	4	ncbi_2006,ncbi_2494,ncbi_101928841,ncbi_445577
novel-m0488-3p	4	ncbi_2006,ncbi_2494,ncbi_101928841,ncbi_445577
novel-m0494-3p	8	ncbi_4846,ncbi_53841,ncbi_285489,ncbi_3014,ncbi_7429,ncbi_11240,ncbi_25823,ncbi_55512
novel-m0506-3p	8	ncbi_4846,ncbi_53841,ncbi_285489,ncbi_3014,ncbi_7429,ncbi_11240,ncbi_25823,ncbi_55512
novel-m0516-5p	1	ncbi_728392
novel-m0517-3p	4	ncbi_2006,ncbi_2494,ncbi_101928841,ncbi_445577
novel-m0518-3p	4	ncbi_2006,ncbi_2494,ncbi_101928841,ncbi_445577
novel-m0519-3p	4	ncbi_2006,ncbi_2494,ncbi_101928841,ncbi_445577
novel-m0525-3p	8	ncbi_4846,ncbi_53841,ncbi_285489,ncbi_3014,ncbi_7429,ncbi_11240,ncbi_25823,ncbi_55512
novel-m0533-5p	1	ncbi_728392
novel-m0535-3p	4	ncbi_2006,ncbi_2494,ncbi_101928841,ncbi_445577
novel-m0536-3p	4	ncbi_2006,ncbi_2494,ncbi_101928841,ncbi_445577
novel-m0544-5p	9	ncbi_9901,ncbi_84439,ncbi_27111,ncbi_478,ncbi_90527,ncbi_100129924,ncbi_60529,ncbi_57161,ncbi_107987276
novel-m0558-5p	2	ncbi_119,ncbi_726
novel-m0583-5p	2	ncbi_119,ncbi_726
novel-m0609-5p	23	ncbi_5896,ncbi_785,ncbi_2824,ncbi_92270,ncbi_151556,ncbi_55186,ncbi_285755,ncbi_81491,ncbi_7358,ncbi_220108,ncbi_257019,ncbi_84449,ncbi_102724488,ncbi_9717,ncbi_57161,ncbi_80032,ncbi_85480,ncbi_136647,ncbi_144321,ncbi_266722,ncbi_105374013,ncbi_7700,ncbi_196500
novel-m0613-5p	48	ncbi_2155,ncbi_2200,ncbi_1439,ncbi_339184,ncbi_440603,ncbi_27122,ncbi_120939,ncbi_79098,ncbi_6925,ncbi_2786,ncbi_347454,ncbi_171024,ncbi_131578,ncbi_4739,ncbi_63901,ncbi_1015,ncbi_4599,ncbi_9058,ncbi_7185,ncbi_5137,ncbi_8479,ncbi_26298,ncbi_834,ncbi_2044,ncbi_2247,ncbi_1634,ncbi_3625,ncbi_1463,ncbi_10893,ncbi_9770,ncbi_23371,ncbi_10060,ncbi_60676,ncbi_64856,ncbi_10129,ncbi_79442,ncbi_79628,ncbi_153478,ncbi_254228,ncbi_4135,ncbi_56606,ncbi_23452,ncbi_2901,ncbi_25903,ncbi_343990,ncbi_729857,ncbi_3479,ncbi_57188
novel-m0614-5p	48	ncbi_2155,ncbi_2200,ncbi_1439,ncbi_339184,ncbi_440603,ncbi_27122,ncbi_120939,ncbi_79098,ncbi_6925,ncbi_2786,ncbi_347454,ncbi_171024,ncbi_131578,ncbi_4739,ncbi_63901,ncbi_1015,ncbi_4599,ncbi_9058,ncbi_7185,ncbi_5137,ncbi_8479,ncbi_26298,ncbi_834,ncbi_2044,ncbi_2247,ncbi_1634,ncbi_3625,ncbi_1463,ncbi_10893,ncbi_9770,ncbi_23371,ncbi_10060,ncbi_60676,ncbi_64856,ncbi_10129,ncbi_79442,ncbi_79628,ncbi_153478,ncbi_254228,ncbi_4135,ncbi_56606,ncbi_23452,ncbi_2901,ncbi_25903,ncbi_343990,ncbi_729857,ncbi_3479,ncbi_57188

MiRNA	Target number	Target gene
novel-m0624-3p	8	ncbi_4846,ncbi_53841,ncbi_285489,ncbi_3014,ncbi_7429,ncbi_11240,ncbi_25823,ncbi_55512
novel-m0632-5p	1	ncbi_728392
novel-m0633-3p	4	ncbi_2006,ncbi_2494,ncbi_101928841,ncbi_445577
novel-m0639-3p	8	ncbi_4846,ncbi_53841,ncbi_285489,ncbi_3014,ncbi_7429,ncbi_11240,ncbi_25823,ncbi_55512
novel-m0647-5p	1	ncbi_728392
novel-m0672-3p	8	ncbi_4846,ncbi_53841,ncbi_285489,ncbi_3014,ncbi_7429,ncbi_11240,ncbi_25823,ncbi_55512
novel-m0680-5p	1	ncbi_728392
novel-m0682-3p	4	ncbi_2006,ncbi_2494,ncbi_101928841,ncbi_445577
novel-m0683-3p	4	ncbi_2006,ncbi_2494,ncbi_101928841,ncbi_445577
novel-m0690-3p	8	ncbi_4846,ncbi_53841,ncbi_285489,ncbi_3014,ncbi_7429,ncbi_11240,ncbi_25823,ncbi_55512
novel-m0698-5p	1	ncbi_728392
novel-m0699-5p	48	ncbi_2155,ncbi_2200,ncbi_1439,ncbi_339184,ncbi_440603,ncbi_27122,ncbi_120939,ncbi_79098,ncbi_6925,ncbi_2786,ncbi_347454,ncbi_171024,ncbi_131578,ncbi_4739,ncbi_63901,ncbi_1015,ncbi_4599,ncbi_9058,ncbi_7185,ncbi_5137,ncbi_8479,ncbi_26298,ncbi_834,ncbi_2044,ncbi_2247,ncbi_1634,ncbi_3625,ncbi_1463,ncbi_10893,ncbi_9770,ncbi_23371,ncbi_10060,ncbi_60676,ncbi_64856,ncbi_10129,ncbi_79442,ncbi_79628,ncbi_153478,ncbi_254228,ncbi_4135,ncbi_56606,ncbi_23452,ncbi_2901,ncbi_25903,ncbi_343990,ncbi_729857,ncbi_3479,ncbi_57188
